# CDK7/12/13 inhibition targets an oscillating leukemia stem cell network and synergizes with venetoclax in acute myeloid leukemia

**DOI:** 10.15252/emmm.202114990

**Published:** 2022-03-07

**Authors:** Lixiazi He, Christian Arnold, Judith Thoma, Christian Rohde, Maksim Kholmatov, Swati Garg, Cheng‐Chih Hsiao, Linda Viol, Kaiqing Zhang, Rui Sun, Christina Schmidt, Maike Janssen, Tara MacRae, Karin Huber, Christian Thiede, Josée Hébert, Guy Sauvageau, Julia Spratte, Herbert Fluhr, Gabriela Aust, Carsten Müller‐Tidow, Christof Niehrs, Gislene Pereira, Jörg Hamann, Motomu Tanaka, Judith B Zaugg, Caroline Pabst

**Affiliations:** ^1^ Department of Medicine V, Hematology, Oncology and Rheumatology University Hospital Heidelberg Heidelberg Germany; ^2^ Molecular Medicine Partnership Unit (MMPU) University of Heidelberg and European Molecular Biology Laboratory (EMBL) Heidelberg Germany; ^3^ European Molecular Biology Laboratory (EMBL) Heidelberg Germany; ^4^ Physical Chemistry of Biosystems Institute of Physical Chemistry Heidelberg University Heidelberg Germany; ^5^ Department of Medical Oncology Dana Farber Cancer Institute Boston MA USA; ^6^ Department of Experimental Immunology Amsterdam Infection & Immunity Institute Amsterdam University Medical Centers Amsterdam Netherlands; ^7^ Centre for Organismal Studies (COS)/Centre for Cell and Molecular Biology (ZMBH) University of Heidelberg Heidelberg Germany; ^8^ German Cancer Research Centre (DKFZ) DKFZ‐ZMBH Alliance Heidelberg Germany; ^9^ Division of Molecular Embryology DKFZ‐ZMBH Alliance Heidelberg Germany; ^10^ Laboratory of Molecular Genetics of Stem Cells Institute for Research in Immunology and Cancer University of Montreal Montreal Quebec Canada; ^11^ Department of Internal Medicine I University Hospital of Dresden Carl Gustav Carus Dresden Germany; ^12^ The Quebec Leukemia Cell Bank and Division of Hematology‐Oncology Maisonneuve‐Rosemont Hospital Montréal Canada; ^13^ Department of Medicine Faculty of Medicine Université de Montréal Montréal Canada; ^14^ Division of Hematology‐Oncology Maisonneuve‐Rosemont Hospital Montreal Quebec Canada; ^15^ Department of Gynecology and Obstetrics University Hospital Heidelberg Heidelberg Germany; ^16^ Department of Surgery Research Laboratories Leipzig University Leipzig Germany; ^17^ Institute of Molecular Biology (IMB) Mainz Germany; ^18^ Center for Integrative Medicine and Physics Institute for Advanced Study Kyoto University Kyoto Japan

**Keywords:** AML, CDK7 inhibition, GPR56, leukemia stem cell, self‐renewal, Cancer, Signal Transduction

## Abstract

The heterogeneous response of acute myeloid leukemia (AML) to current anti‐leukemic therapies is only partially explained by mutational heterogeneity. We previously identified GPR56 as a surface marker associated with poor outcome across genetic groups, which characterizes two leukemia stem cell (LSC)‐enriched compartments with different self‐renewal capacities. How these compartments self‐renew remained unclear. Here, we show that GPR56^+^ LSC compartments are promoted in a complex network involving epithelial‐to‐mesenchymal transition (EMT) regulators besides Rho, Wnt, and Hedgehog (Hh) signaling. Unexpectedly, Wnt pathway inhibition increased the more immature, slowly cycling GPR56^+^CD34^+^ fraction and Hh/EMT gene expression, while Wnt activation caused opposite effects. Our data suggest that the crucial role of GPR56 lies in its ability to co‐activate these opposing signals, thus ensuring the constant supply of both LSC subsets. We show that CDK7 inhibitors suppress both LSC‐enriched subsets *in vivo* and synergize with the Bcl‐2 inhibitor venetoclax. Our data establish reciprocal transition between LSC compartments as a novel concept underlying the poor outcome in GPR56^high^ AML and propose combined CDK7 and Bcl‐2 inhibition as LSC‐directed therapy in this disease.

## Graphical abstract

RNA‐ and ATAC‐seq profiling combined with functional *in vitro* and *in vivo* studies unravel the multi‐faceted roles of GPR56, a surface marker associated with high leukemia stem cell (LSC) burden and poor prognosis in acute myeloid leukemia (AML).

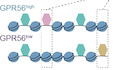

**The paper explained**

**Problem**

Therapy response is highly heterogeneous in acute myeloid leukemia (AML), which is only partially explained by genetic risk groups. The adhesion G protein coupled receptor GPR56/ADGRG1 was previously identified as surface marker associated with poor outcome across genetic groups and characterizes two leukemia stem cell (LSC)‐enriched compartments with different self‐renewal capacities. Whether GPR56 played a functional role in this process and how this might be targeted remained unclear.

**Results**

GPR56 is functionally required for the maintenance of human LSC‐enriched compartments by regulating Wnt, Hedgehog (Hh), and EMT‐associated genes in addition to RhoA signaling. While reporter assays and functional tests show that these pathways are all enhanced by GPR56, Wnt and Hh/TGFb pathways reciprocally inhibit each other, and their target genes are differentially expressed in the CD34^+^GPR56^+^ versus CD34^‐^GPR56^+^ LSC‐enriched fractions. Cell sorting and *in vivo* experiments provide evidence for reciprocal transition between the two compartments. In particular, combination of a Wnt inhibitor with a Hh agonist and TGFb is sufficient to regenerate the CD34^+^GPR56^+^ from the CD34^‐^GPR56^+^ compartment *in vitro*. The CDK7/12/13 inhibitor THZ1 and more specific CDK7 inhibitors synergize with the Bcl‐2 antagonist venetoclax to suppress both GPR56^+^ compartments in primary human AML cells *in vitro* and *in vivo*.

**Impact**

The study unravels a complex network around GPR56 in AML, thus raising it from a simple marker that enriches for LSCs to a functional stemness regulator with a pivotal role in preventing exhaustion of the LSC pool. The study puts forward a model of reciprocal transition between functionally distinct LSC compartments, rapidly and slowly cycling, which is aberrant in AML compared with the healthy hematopoietic system. This model provides an explanation on how LSCs escape current chemotherapies and suggests that synergistically acting CDK7 and Bcl‐2 inhibitors might represent a novel therapeutic approach to target the GPR56‐associated network in AML.

## Introduction

Acute myeloid leukemia (AML) is a hematologic malignancy affecting both young and elderly patients, for whom intensive therapies are often not an option (Döhner *et al*, 2015). Assessment of cytogenetic and molecular genetic aberrations has become the gold standard for risk stratification and for guiding therapeutic decisions for AML patients harboring targetable mutations (Döhner *et al*, 2017). Targeting mutated proteins by small molecules such as IDH1/2 or FLT3 inhibitors has considerably broadened the therapeutic repertoire in AML and improved survival (reviewed in refs. Kindler *et al*, 2010; Chaturvedi *et al*, 2013). However, when given as monotherapy, they usually delay leukemia progression rather than permanently eradicate the disease (Kindler *et al*, 2010). Moreover, there is large heterogeneity in patient outcome even within previously defined genetic groups (Cancer Genome Atlas Research Network *et al*, 2013) demonstrating the need to better understand how targetable and non‐targetable mutations together with the induced downstream pathways synergize to drive the disease. While RNA‐seq is powerful in detecting global gene expression changes in homogeneous populations, it may miss subtle changes in lowly abundant mRNAs such as transcription factors (TFs), especially in heterogeneous populations such as primary AML samples. Furthermore, expression levels of TFs are not necessarily indicative of their activity, which is often regulated posttranslationally (Filtz *et al*, 2014). We and others have shown that epigenetic analyses are often more powerful in detecting differences between heterogeneous primary samples, as they also reveal the epigenetic potential (cell fates) rather than only events that have already happened (cell states; Assi *et al*, 2019; Berest *et al*, 2019). Besides genetic subtyping, AML can be characterized by shared signaling pathways and aberrant immunophenotypes such as co‐expression of CD7, CD56 (Chang *et al*, 2004), or a CD34^low^GPR56^high^ profile, the latter of which we associated with co‐mutations in *NPM1*, *DNMT3A*, and *FLT3*‐ITD (Garg *et al*, 2019), and high LSC frequency (Pabst *et al*, 2016). In AML with an aberrant CD34^low^GPR56^high^ profile, both the CD34 positive and negative GPR56^+^ fractions contain LSCs. Besides establishing GPR56 as an LSC marker, we showed that high GPR56 expression is associated with poor prognosis in AML (Pabst *et al*, 2016). In line, GPR56 is part of a 17‐gene stemness signature associated with poor prognosis in AML (Ng *et al*, 2016). GPR56 belongs to the adhesion G‐protein coupled receptor (aGPCR) family characterized by a 7‐transmembrane (7TM) domain, flanked by an intracellular C‐terminus and a long extracellular N‐terminus, which contains the GPCR proteolytic site (GPS) within the GPCR autoproteolysis‐inducing (GAIN) domain (Chiang *et al*, 2011; Purcell & Hall, 2018). Splice variants have been described to possess overlapping but also non‐redundant functions, which might explain why different knock‐out strategies caused distinct phenotypes (Kim *et al*, 2010b; Rao *et al*, 2015; Li *et al*, 2020). The impact of GPR56 on Rho signaling and actin cytoskeleton regulation suggested a predominant role in adhesion as shown in the neuronal system (Iguchi *et al*, 2008) and certain solid tumors (Shashidhar *et al*, 2005). Despite the associations of GPR56 with poor prognosis in several different cancer entities (Shashidhar *et al*, 2005; Kausar *et al*, 2011) and endothelial‐to‐hematopoietic transition (Solaimani Kartalaei *et al*, 2015), it was unclear whether and how GPR56 was functionally involved in LSC activity in human AML and how its downstream pathways might be targeted.

Here, we explored chromatin accessibility and transcription factor activities through ATAC‐seq profiling of 35 primary human AML samples followed by RNA‐seq, functional assays, and preclinical models to dissect the role of GPR56 in the identified network. Our data suggest a model, in which GPR56 by co‐activating reciprocally inhibitory signals promotes oscillation of signaling pathways that drive either towards the CD34 positive or negative GPR56^+^ LSC compartment, which differ by LSC frequency and cycling characteristics (Pabst *et al*, 2016). Moreover, we identify CDK7/12/13 inhibitors as novel compound class that targets diverse routes in this network and synergizes with the Bcl‐2 inhibitor venetoclax to eradicate primary human AML cells.

## Results

### ATAC‐ and RNA‐seq profiling link GPR56 to increased TF activities associated with EMT, Wnt and Hh signaling

**Figure 1:**
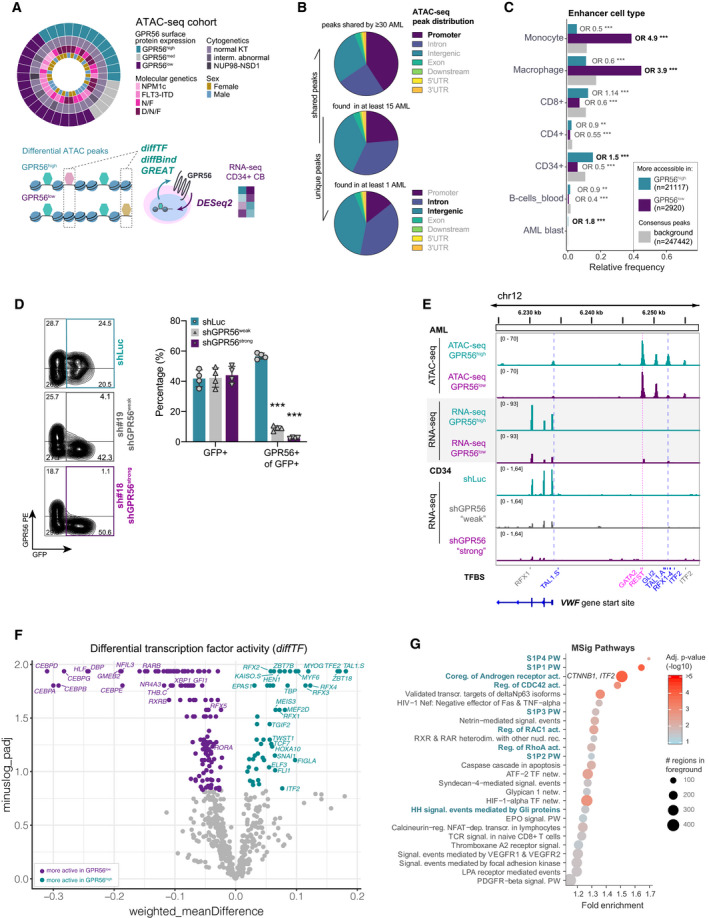
ATAC‐ and RNA‐seq profilings position GPR56 within a Rho/Wnt/Hh network Overview of the experimental and analytical setup. ATAC‐seq was performed on 35 AML samples comprising the mutational groups N: *NPM1* mutated, F: *FLT3*‐ITD, N/F: *NPM1*/*FLT3*‐ITD, D/N/F: *DNMT3A*/*NPM1*/*FLT3*‐ITD. Indicated colors represent GPR56 protein expression grouped into high (> 70% GPR56^+^ cells per sample, *n* = 13), medium (30%–70% GPR56^+^, *n* = 5), and low (< 30% GPR56^+^, *n* = 17), cytogenetic and molecular genetic characteristics, and gender. ATAC‐seq data were subjected to the computational pipelines *diffTF*, *diffBind*, and *GREAT*. RNA‐seq was performed on cord blood (CB) CD34^+^ cells after GPR56 knockdown and analyzed by DESeq2. The combined information was used to identify differential TF and signaling pathway activities up‐ and downstream of GPR56.Pie charts showing the distribution of ATAC‐seq peaks shared by most samples (top), by at least 15 samples (middle), and those present in at least one sample. Colors indicate the different genomic regions. Chromatin regions that are rather differentially accessible (bottom) are enriched for introns and intergenic regions, which often contain regulatory elements, while shared peaks are more often located in promoter regions (top).ATAC‐seq peaks more accessible in GPR56^low^ (violet) or GPR56^high^ (turquoise) against the background (gray) are enriched for enhancers associated with specific hematopoietic cell types. *P*‐values and odds ratios are given for a pairwise, two‐sided Fisher’s Exact test comparing each category (GPR56^low/high^) against the background. Enhancer annotations are taken from EnhancerAtlas 2.0. ***P < 0.0005, **P < 0.005, *P < 0.05.Knockdown efficiency of two shRNAs against *GPR56* (shGPR56^weak^ and shGPR56^strong^) versus shLuc as negative control measured on protein level by flow cytometry in CD34^+^ CB cells. Shown are representative FACS plots (left) and the percentage of GPR56^+^ cells of transduced GFP^+^ cells on day 5 (right panel). Biological replicates *N* = 4, unpaired *t*‐test, bars and error bars represent mean and SD. ***P < 0.0005, **P < 0.005, *P < 0.05.Integrative Genome Viewer (IGV) snapshot showing ATAC‐seq peaks along and upstream of the *VWF* gene in GPR56^high^ vs. ^low^samples (top, average peak size of 10 GPR56^high^ (turquoise) and 15 GPR56^low^ samples (violet)), RNA‐seq reads of the same location in AML samples with high (*n* = 9) versus low (*n* = 11) GPR56 expression (two middle tracks), and RNA‐seq reads of shLuc versus GPR56 knockdown CD34^+^ cells (3 bottom tracks, one of two replicates shown for each condition). Track height was group‐scaled. Dashed vertical lines indicate binding sites for the annotated TFs. TFBS: transcription factor‐binding site derived from the *HOCOMOCO* v10 database; TSS: transcription start site. TFs in blue bind to differentially accessible chromatin regions.Volcano plot of differential TF motif accessibility (activity) in GPR56^high^ (turquoise) vs. GPR56^low^ (violet) samples and their corresponding adjusted *P*‐values determined with *diffTF*. Highlighted are TFs whose RNA expression was also positively or negatively affected by GPR56 KD in the RNA‐seq dataset.Pathway enrichment analysis for peaks that are more accessible in GPR56^high^ AML. The GREAT algorithm was used to assign peaks to genes, the *MSig* database was used for pathway enrichment analysis (*Pathway Interaction Database*). Shown are all terms with adjusted *P*‐value < 0.05. PW: pathway, (Co‐)reg.: (Co‐) regulation, act.: activity, NR: nuclear receptor, transcr.: transcription(al), netw.: network, signal.: signaling.

We applied ATAC‐seq to 35 primary human AML samples, which represented the full range from low to very high protein expression levels of the LSC marker GPR56, to identify leukemia‐driving pathways in GPR56^high^ AML (Fig 1A, Dataset EV1). We identified 247,442 unique chromatin peaks, of which 24,026 were differentially accessible in GPR56^high^ versus (vs) GPR56^low^ AML (false discovery rate, FDR < 5%, see Fig 1A legend for grouping criteria). The shared peaks (32,406 peaks found in at least 30 samples) were enriched in promoters, while more sample‐specific peaks were enriched in intergenic/intronic regions (Fig 1B). As the latter often contain regulatory elements, we interrogated EnhancerAtlas 2.0 (Gao & Qian, 2020) and found enrichment for enhancers with known activity in CD34^+^, AML blasts, and CD8^+^ T‐cells among the GPR56^high^‐specific peaks, whereas enhancers associated with monocytes and macrophages were significantly enriched in the GPR56^low^ group (Fig 1C). These results support previous observations that AML with high GPR56 expression represents a more immature, poorly differentiated type of AML (Pabst *et al*, 2016).

To assess the role of GPR56 in normal hematopoietic stem and progenitor cells (HPSCs), we performed a knockdown (KD) of GPR56 in cord blood (CB) CD34^+^ cells using shRNAs against GRP56 or luciferase (shLuc) as negative control, followed by RNA‐seq (Fig 1D and E). The CB CD34^+^ cells are hereafter called shGPR56^weak^ and shGPR56^strong^ according to the shRNA’s different efficiency to suppress GPR56 surface expression on transduced GFP^+^ cells (Fig 1D, Appendix Fig S1A), which might be explained by the different shRNA target sequence localization (Appendix Fig S1B; Kim *et al*, 2010b). CB CD34^+^ cells were used as representative, non‐mutated CD34^+^GPR56^+^ model cell type (Pabst *et al*, 2016) to avoid AML sample‐specific results. GO term enrichment analysis of differentially expressed genes determined with *Deseq2* revealed “GPCR signaling”, “adhesion” and “migration”, in line with the known role of GPR56 but also pointed toward platelet associated processes and phospholipase activity (Appendix Fig S1C and D, Dataset EV2).

We further explored the ATAC‐seq data by applying our computational tool *diffTF,* which estimates differential TF activities on the basis that chromatin accessibility increases when TFs interact with chromatin at specific TF‐binding sites and thus contribute to the ATAC‐seq signal (Assi *et al*, 2019; Berest *et al*, 2019). In addition, we overlayed *diffTF* results with the RNA‐seq data to assess whether gene expression of differentially active TFs or their respective target genes was affected by GPR56 KD, which would suggest that they act downstream of GPR56. To illustrate how differential TF activities were identified in *diffTF*, we visualized one example region upstream of the *VWF* gene locus (Fig 1E) using the integrative genome viewer (IGV). Two differential peaks with stronger ATAC‐seq signal in GPR56^high^ vs. GPR56^low^ AML were identified upstream of and at the gene start site. These contained binding sites for TAL1, GLI2, RFX1‐4, and ITF2, while binding motifs for GATA2 and REST were similarly accessible in GPR56^high^ and GPR56^low^ AML (Fig 1E, upper row). Moreover, *VWF* mRNA expression was higher in GPR56^high^ vs GPR56^low^ AML (RNA‐seq from (Garg *et al*, 2019), Fig 1E, middle row). Finally, the shRNA‐seq dataset revealed a downregulation of *VWF* in CD34^+^ cells after GPR56 suppression with both shRNAs (Fig 1E, lower row). Together, these observations indicated that GPR56 functionally upregulates *WVF* expression, potentially upstream of RFX1‐4 and GLI2, which are known TFs in the HH pathway. When applying this approach on a genome‐wide level using *diffTF*, we identified 166 TFs with significantly different activities in the GPR56^high^ vs ^low^ group at an FDR of 10% (Fig 1F and Dataset EV3). These comprised several TFs related to Wnt and Hh pathways such as *TCF7* (Stemmer *et al*, 2008), *TFE2/TCF3* (Zhao *et al*, 2019), and *RFX1‐4* (Piasecki *et al*, 2010) in the GPR56^high^ group, while the GPR56^low^ group was characterized by higher CEBPA activity (ATAC‐seq), a TF essential for myeloid differentiation (Kandilci & Grosveld, 2009). The EMT‐associated TFs SNAIL1, TWIST1 (Nieto *et al*, 2016), and TGIF2 (Du *et al*, 2019) were also more active in GPR56^high^ samples (Fig 1F) but were not affected by GPR56 suppression (Dataset EV2) suggesting that they act upstream of GPR56. In support of this hypothesis, suppression of *SNAIL1* and *ITF2/TCF4* reduced *GPR56* mRNA in external RNA‐seq datasets (GSE70872, GSE38236, and Doostparast Torshizi *et al*, 2019). We subsequently confirmed suppression of *GPR56* upon *ITF2* knockdown in our own q‐RT‐PCR experiments (Appendix Fig S1E, Dataset EV4).

To gain more insight into the pathways more active in GPR56^high^ AML, we applied the tool ‘GREAT’ (McLean *et al*, 2010) to the ATAC‐seq data, which identified enrichment for Rho signaling, a well‐established GPR56 downstream pathway (Iguchi *et al*, 2008), but also pointed toward the Wnt genes *CTNNB1* (β‐catenin) and *ITF2/TCF4* (Kolligs *et al*, 2002) contained in “Coregulation of Androgen receptor activity”, as well as “Hh signaling events mediated by Gli proteins” (Chen *et al*, 2009; Fig 1G, Dataset EV5). We therefore screened the RNA‐seq dataset for *bona fide* Wnt and Hh genes (Dataset EV6) and found significant downregulation upon GPR56 suppression for Wnt pathway genes such as *DVL1, TNKS2, VEGFA, MYC, CCND1* (He *et al*, 1998; Shtutman *et al*, 1999; Corbit *et al*, 2005; Zhan *et al*, 2017), as well as *SMO*, the key Hh pathway effector (Corbit *et al*, 2005) (Appendix Fig S1F).

Together, these analyses pointed toward involvement of Wnt, Hh, and EMT regulators in GPR56^high^ AML in addition to Rho.

### GPR56 is required for *in vitro* and *in vivo* expansion of primary human AML cells

**Figure 2:**
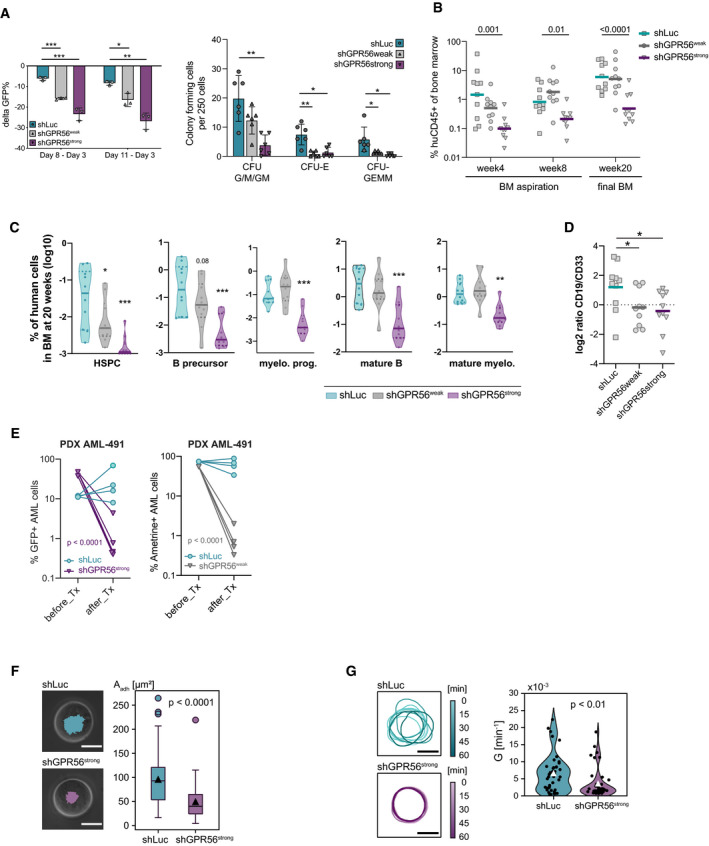
Functional role of GPR56 in healthy CD34^+^ and leukemic cells Functional role of GPR56 *in vitro*. *Left*: CB CD34^+^ cells were transduced with shGPR56^weak^ and shGPR56^strong^ versus shLuc as negative control. The loss of the GFP^+^ fraction on day 8 and day 11 compared with day 3 post transduction (delta GFP^+^) correlates with the level of GPR56 KD. Three replicate wells were monitored per condition, unpaired *t*‐test, bars and error bars represent mean and SD, ***P < 0.0005, **P < 0.005, *P < 0.05. *Right*: Colony‐forming cell assay. Pooled CB CD34^+^ cells were transduced with shRNAs against GPR56 or shLuc control and GFP^+^ cells were directly sorted into methylcellulose 72 h post transduction. Two hundred fifty GFP^+^ cells were plated per well, and colonies were counted 10 days post plating. CFC: colony forming cell, CFU: colony forming unit, G: granulocyte, M: macrophage, GM: granulocyte/macrophage, E: erythrocyte, GEMM: granulocyte, erythrocyte, megakaryocyte, macrophage. Shown is one of two independent assays. Six wells were plated per condition, unpaired *t*‐test, bars and error bars represent mean and SD. *P < 0.05, **P < 0.005.Functional role of GPR56 *in vivo*. CB CD34^+^ cells were transduced with the two shRNAs against GPR56 versus shLuc as negative control. Gene transfer was approximately 50% at experiment start. Equal numbers of cells were injected in 10 recipient NSG mice per condition, and engraftment of human CD45^+^ (huCD45) cells in the bone marrow was assessed at 4 and 8 weeks by bone marrow aspiration and at 20 weeks by total bone marrow harvest. Strong GPR56 KD (shGPR56^strong^) significantly reduced overall human CD45^+^ engraftment, while incomplete KD (shGPR56^weak^) showed a trend toward lower engraftment at 4 weeks, but no difference at later time points indicating that low GPR56 surface levels might be sufficient for LT‐HSC engraftment. Unpaired *t*‐test of log_10_‐transformed values, symbols represent individual mice, horizontal bars represent geometric means, BM: bone marrow.Violin plots showing geometric mean (horizontal line) and individual values (circles) of the engraftment levels of different hematopoietic cell types in the bone marrow of NSG mice 20 weeks after transplantation of CB CD34^+^ cells following overnight‐infection with shGPR56^strong^ (turqouise) and shGPR56^weak^ (gray) versus shLuc control (violet). Shown are percentages of indicated populations, which are also co‐positive for human CD45 and GFP, relative to all harvested bone marrow cells. HSPC: CD34^+^SSC^low^CD33^−^CD19^−^, B precursor: CD34^+^CD19^+^CD33^−^, myeloid progenitor (myelo. prog.): CD34^+^CD33^+^CD19^−^, mature B cells: CD19^+^CD34^−^, mature myeloid cells: CD33^+^CD19^−^CD34^−^. Multiple unpaired *t*‐tests of log‐transformed values, *P*‐values were Benjamini and Hochberg corrected (*P*_adj_), *P_adj_ < 0.05, **P_adj_ < 0.005, ***P_adj_ < 0.0005. See Appendix Fig S2A for gating strategy.Log_2_‐fold changes (log_2_FC) of the CD19^+^ (B lymphoid) versus CD33^+^ (myeloid) cell ratios within the human CD45^+^GFP^+^ fractions at final analysis 20 weeks after transplantation. Both shRNAs change the lymphoid/myeloid ratio in favor of myeloid cells. Unpaired *t*‐test of log_2_FC, symbols represent individual mice, horizontal bars represent average log_2_FC, *P < 0.05.GPR56 KD results in highly reduced engraftment of AML‐491 in mice. *Left*: Cells from two independent infections with shGPR56^strong^ were injected in 4 NRGS mice. Shown are fractions of GFP^+^ cells before and 10 weeks after transplantation (Tx). Unpaired *t*‐tests of log(%GFP^+^) before versus after transplantation. *Right:* weak GPR56 KD results in highly reduced engraftment of AML‐491 in NSGW41 mice. Cells were injected in 4 recipient NSGW41 mice. Shown are fractions of positively transduced Ametrine (AM)^+^ cells before and 32 weeks after Tx. Unpaired *t*‐tests of log(%AM^+^) before versus after transplantation. No difference in overall leukemic engraftment (including non‐transduced cells) was observed excluding technical issues with injections (Appendix Fig S2C).Adhesion of control and GPR56 KD K562 cells on fibronectin‐functionalized substrates. *Left:* Overlay of phase contrast and microinterferometry images of control (above) and GPR56 KD cells (below). The area of tight adhesion extracted by microinterferometry is highlighted in turquoise and violet, respectively. *Right*: Comparison of adhesion areas extracted by microinterferometry, reduction of adhesion area by factor 1.9 in GPR56 KD cells (*P* = 1.0 × 10^−10^, two‐sided Mann‐Whitney test, box plots showing medians, quartiles, and outliers according to the Tukey method). Technical replicates *N*_control_ = 71, Technical replicates *N*_KD_ = 104, scale bar 10 µm.Active deformation of control and GPR56 KD cells. *Left*: Cell periphery of control (top) and GPR56 KD (bottom) K562 cells tracked over 60 min. *Right*: Comparison of deformation power of control and GPR KD cells, showing a reduction of deformation power by factor 1.8 (*P* = 0.005, two‐sided Mann‐Whitney test, white triangles of the violin plot represent mean). Technical replicates *N*_control_ = 40, Technical replicates *N*_KD_ = 40, scale bar 10 µm.

To further characterize the functional role of GPR56, we determined the effects of GPR56 KD on human CD34^+^ CB and AML cells. Both shRNAs significantly impaired CD34^+^ cell proliferation and colony formation capacity (Fig 2A, Dataset EV7). For the *in vivo* experiment, we used unsorted cells with comparable gene transfer in the three conditions prior to injection (40–50% GFP^+^, as shown in Fig 1D). Strong GPR56 suppression (shGPR56^strong^) significantly hampered short‐ (ST) and long‐term (LT) HSC engraftment in NSG mice (Fig 2B, Dataset EV7). Analysis of hematopoietic subpopulations at 20 weeks revealed that shGPR56^strong^ had depleted the HSPC compartment in most mice and significantly affected the B lymphoid lineage stronger than the myeloid lineage (Fig 2C, Appendix Fig S2A, Dataset EV7), resulting in an altered CD19^+^/CD33^+^ ratio (Fig 2D). ShGPR56^weak^ only showed a trend towards lower engraftment at four weeks and had no disadvantage in overall human CD45^+^GFP^+^ engraftment at later time points. However, it also significantly reduced the rare HSPC compartment (CD34^+^CD33^−^CD19^−^SSC^low^) (Fig 2C) and significantly reduced the CD19^+^/CD33^+^ ratio similarly to shGPR56^strong^ (Fig 2D). These results suggested that high expression levels of GPR56 are functionally important for the maintenance of the more primitive human HS(P)C compartments, while low levels are sufficient to maintain committed progenitors and their progeny *in vivo*.

To assess the effect of GPR56 suppression on leukemic cells, we transduced eight AML cell lines and observed that four of the five lines most sensitive to GPR56 suppression harbored mutations in either *NPM1* (OCI‐AML3), *DNMT3A* (OCI‐AML2, OCI‐AML3), *FLT3*‐ITD (MV4‐11), or had a *MECOM*/*EVI1* overexpression (HNT34), which represent genetic groups that we had previously connected with high GPR56 expression (Pabst *et al*, 2016) (Appendix Fig S2B, Dataset EV8). To determine whether GPR56 suppression affected the leukemia initiating capacity, a hallmark of LSCs (Lapidot *et al*, 1994), we transplanted patient‐derived xenograft cells in immunocompromised mice after short overnight lentiviral transduction to avoid effects of GPR56 suppression before injection. We observed that both strong and weak GPR56 suppression significantly hampered leukemic engraftment in mice, while overall human CD45^+^ levels including non‐transduced cells were similarly high in all mice and thus excluded technical issues during transplantation (Fig 2E, Appendix Fig S2C, Dataset EV9).

Given the established connection between GPR56 and Rho signaling, we determined the effects of GPR56 suppression on adhesive and migratory properties of leukemic cells by using microinterferometry. These assays were established by us before and proven to reflect the adhesion properties of hematopoietic stem and leukemic cells (Burk *et al*, 2015). To obtain a sufficient number of transduced cells with comparable viability, we selected the K562 cell line for this assay, as these cells were less affected by GPR56 KD within the first days post transduction compared to other cell types (Appendix Fig S2B). This assay revealed a significant reduction of the tight adhesion area on fibronectin‐coated glass slides after GPR56 suppression (Fig 2F). Moreover, a high‐throughput assay utilizing pressure waves induced by pico‐second laser pulse (Yoshikawa *et al*, 2011; Burk *et al*, 2015) revealed a significant reduction of the critical pressure for detachment in GPR56 KD vs. control conditions (Appendix Fig S2D). In addition, we estimated the active deformation of cells by tracing the cell periphery over time (Burk *et al*, 2015; Lamas‐Murua *et al*, 2018), which was significantly reduced in GPR56 KD vs control cells (Fig 2G). In summary, these studies provided strong evidence for a functional role of GPR56 in healthy and leukemic HSPCs and confirmed a major impact of GPR56 on the adhesion and deformation capacity of human leukemic cells.

### GPR56 enhances Wnt and Hedgehog pathways

**Figure 3:**
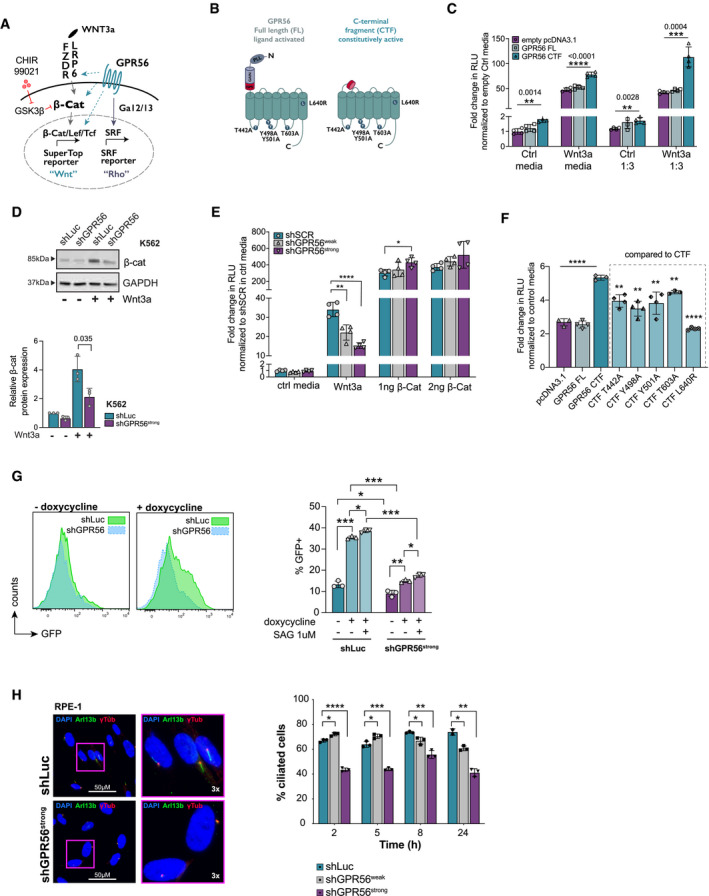
GPR56 modulates Wnt and Hh signaling pathways Cartoon illustrating at which levels GPR56 may enhance canonical Wnt signaling (turquoise dashed arrows): at the level of ligand‐receptor/co‐receptor interaction, β‐catenin, downstream of β‐catenin, indirectly via activation of Rho/SRF through Gɑ12/13. CHIR99021 is a GSK3 inhibitor that prevents degradation of β‐catenin thus enhancing Wnt signaling.Cartoon illustrating GPR56 full length (FL, *left*) and the C‐terminal fragment (CTF, *right*). Amino acid changes introduced in the intracellular loops are indicated by circles and letters. GAIN: GPCR autoproteolysis‐inducing domain, GPS: GPCR proteolytic site, PLL: Pentraxin/Laminin/neurexin/sex‐hormone‐binding‐globulin‐Like (Salzman *et al*, 2016).SuperTop reporter assay showing fold change of relative luminescence units (RLU) in Wnt3a conditioned culture media normalized to empty pcDNA3.1^+^ in the respective control (Ctrl) media after transfection of HEK293T cells with empty vector (violet), GPR56 FL (light turquoise), and GPR56 CTF (turquoise). Shown are means, SD, and individual values of four technical replicates performed in either complete media or 1:3 diluted Wnt3a conditioned media. Unpaired *t*‐test, ****P < 0.0001, ***P < 0.0005, **P < 0.005.β‐catenin protein expression in K562 cells infected with shGPR56^strong^ or shLuc negative control in presence and absence of Wnt3a (representative Western Blot (above) and quantification after normalization to GAPDH (below)). Shown is fold‐change compared with shLuc in control media, bars and error bars represent mean and SD of three biological replicates, unpaired *t*‐test.SuperTop reporter assay showing fold‐change of RLU in presence of Wnt3a normalized to a scrambled shRNA (shSCR) in control media after transfection with shSCR, shRNAs against GPR56 with or without additional transfection with 1 ng or 2 ng of β‐catenin plasmid. Overexpression of β‐catenin fully rescues the SuperTop signal reduction caused by shRNAs against GPR56. Unpaired *t*‐test, bars, and error bars represent mean and SD of four biological replicates, ****P < 0.0001, ***P < 0.0005, **P < 0.005, *P < 0.05.SuperTop reporter assay indicating fold change in RLU normalized to control media after transfection of HEK293T cells with empty vector, GPR56‐FL, or 5 GPR56‐CTF mutants. Unpaired *t*‐test, bars, and error bars represent mean and SD of four biological replicates, ****P < 0.0001, **P < 0.005.
*Left*: SMO‐GFP reporter RPE cell line or RPE‐1 wild‐type cells were infected with shGPR56^strong^ or shLuc control followed by administration of doxycycline or the SMO agonist SAG as indicated. GPR56 KD significantly reduces the baseline and doxycycline‐induced SMO‐GFP signal. *Right*: representative FACS histogram plots showing GFP intensity in shLuc versus shGPR56^strong^ RPE cells with and without doxycycline. Unpaired *t*‐test, bars, and error bars represent mean and SD of three biological replicates, ***P < 0.0005, **P < 0.005, *P < 0.05.
*Left*: Representative immunofluorescence (IF) images showing primary cilium formation in RPE cells upon starvation after infection with shLuc (above) or shGPR56^strong^ (below). The non‐motile primary cilium is visualized using antibodies against γ‐tubulin, which stains the basal body, and the ciliary membrane marker ARL13B. DAPI indicates the nucleus. Images on the right show selected cells at 3× magnification. Brightness was increased in all images to enhance visibility of cilia. *Right*: Percentage of RPE‐1 cells with primary cilium in shLuc vs. shGPR56^strong^ RPE‐1 cells at different time points after the start of serum starvation. Unpaired *t*‐test, bars, and error bars represent mean and SD of three biological replicate wells. ****P < 0.0001, ***P < 0.0005, **P < 0.005, *P < 0.05.

As our ATAC‐ and RNA‐seq data pointed towards a role of GPR56 in Wnt and Hh pathways, we next sought to investigate these interactions mechanistically. To test whether and how GPR56 may enter the Wnt pathway (Fig 3A illustrating potential scenarios), we generated full length (FL) and constitutively active C terminal fragment (CTF) versions of GPR56 (Kishore *et al*, 2016) and used a standard Wnt luciferase reporter assay (“SuperTop”, modified Topflash) as readout (Fig 3B, Appendix Fig S3A). Adhesion GPCRs undergo autoproteolytic cleavage at the GPCR proteolytic site (GPS), after which the N‐terminus stays non‐covalently connected with the CTF. Conformational changes through ligand binding or mechanical stimuli can expose the most N‐terminal part of the CTF, also referred to as Stachel‐peptide, which may then act as a tethered agonist to induce signaling (Liebscher *et al*, 2014). CTF versions mimicking this process are therefore widely used to provide a ligand‐independent constitutive signal that can be assessed in reporter assays. Overexpression of GPR56‐CTF significantly enhanced the baseline and Wnt3a‐induced SuperTop signal (Fig 3C). We did not observe an increased Wnt reporter signal with MAL/MKL1, a strong Rho activating positive control (Miralles *et al*, 2003), ruling out that the GPR56‐induced enhancement of Wnt signaling occurs via its activating effect on RhoA (Appendix Fig S3B). Conversely, Wnt3a‐conditioned media did not enhance the signal in a Serum Response Factor Response Element (SRF) luciferase reporter (Miralles *et al*, 2003) used to detect RhoA signaling (Appendix Fig S3C). Furthermore, we observed significant reduction of the Wnt3a‐induced cytosolic accumulation of β‐catenin by GPR56 KD (Fig 3D) suggesting that GPR56 acted upstream of β‐catenin. In support of this, transfection of HEK293T cells with a β‐catenin expression plasmid led to a complete rescue of the shGPR56‐mediated reduction in the Wnt3a‐induced signal (Fig 3E). In line, there was no signal reduction by shRNAs against GPR56 upon usage of the GSK3 inhibitor CHIR99021 to induce the Wnt reporter signal (Appendix Fig S3D). Since our results suggested that GPR56 acted upstream of β‐catenin, we assessed the effect of GPR56 on the essential Wnt co‐receptor LRP6. We observed that the Wnt3a‐induced increase in LRP6 protein was significantly suppressed by GPR56 KD (Appendix Fig S3E). In addition, GPR56 CTF was unable to generate a Wnt signal in an LRP6^−/−^ knockout (KO) HEK293 cell line (Appendix Fig S3F) indicating that the effect of GPR56 on Wnt signaling occurred on the receptor level. Finally, we introduced several missense mutations in the intracellular loops of GPR56 causing amino acid changes in potential phosphorylation sites and included also one mutation in the 7^th^ transmembrane (TM) domain (L640R), which was reported to cause bilateral frontoparietal polymicrogyria (Piao *et al*, 2004) (Appendix Fig S3A). All mutants reduced the signal in both, SRF and Wnt reporter assays compared with non‐mutated CTF (Fig 3F, Appendix Fig S3G), suggesting that these positions have conserved roles in GPR56 receptor activation in Wnt and SRF (RhoA) signaling. Together, these results showed that GPR56 promoted Wnt signaling on the co‐receptor level.

To characterize the role of GPR56 in the Hh pathway, we utilized the retinal pigment epithelial cell line RPE‐1, since the Hh pathway proteins have been localized to the primary cilium, which has best been studied and visualized in this cell line (Corbit *et al*, 2005; May‐Simera *et al*, 2018). In addition to the RPE‐1 wild type (wt) cells, we used a well‐established doxycycline inducible SMO‐GFP reporter RPE‐1 cell line (Joo *et al*, 2013); (May‐Simera *et al*, 2018), as visualization of endogenous SMO protein is hampered by the very low expression levels. First, we tested whether GPR56 suppression also downregulated *SMO* and β‐catenin in the RPE‐1 cell line and confirmed that the effects of GPR56 KD on Wnt and Hh pathways were conserved in this cell type (Appendix Fig S3H and I). Administration of doxycycline to the RPE‐1 SMO‐GFP cells significantly increased the 10% baseline SMO‐GFP expression, which was further enhanced by the SMO agonist SAG (Fig 3G). GPR56 suppression, in contrast, significantly decreased baseline and doxycycline‐stimulated SMO‐GFP signals. Furthermore, GPR56 suppression caused a significant reduction in the fraction of ciliated RPE‐1 cells (Fig 3H). Measurement of cilium length over time revealed significantly longer cilia early after serum‐starvation with both shRNAs (Appendix Fig S3J), which is a typical phenomenon in ciliopathies (Kim *et al*, 2010a). When combining basal body with ɑ‐tubulin staining, GPR56 suppression strongly altered the cell shape and the ɑ‐tubulin architecture from a horizontally aligned to a rather radially organized network surrounding the γ‐tubulin mark (Appendix Fig S3K). Interestingly, in a public RNA‐seq dataset (GSE147727, Dataset EV10), *SMO* suppression significantly reduced *GPR56* mRNA levels, which might hint at positive feedback between GPR56 and Hh pathway. In summary, these mechanistic studies confirmed the ATAC‐/RNA‐seq‐driven hypotheses that GPR56 plays a functional role in Wnt and Hh signaling.

### TGFb, HH, and Wnt pathway activity determine the balance between the GPR56^+^CD34^+^ and GPR56^+^CD34^−^ LSC compartments

**Figure 4:**
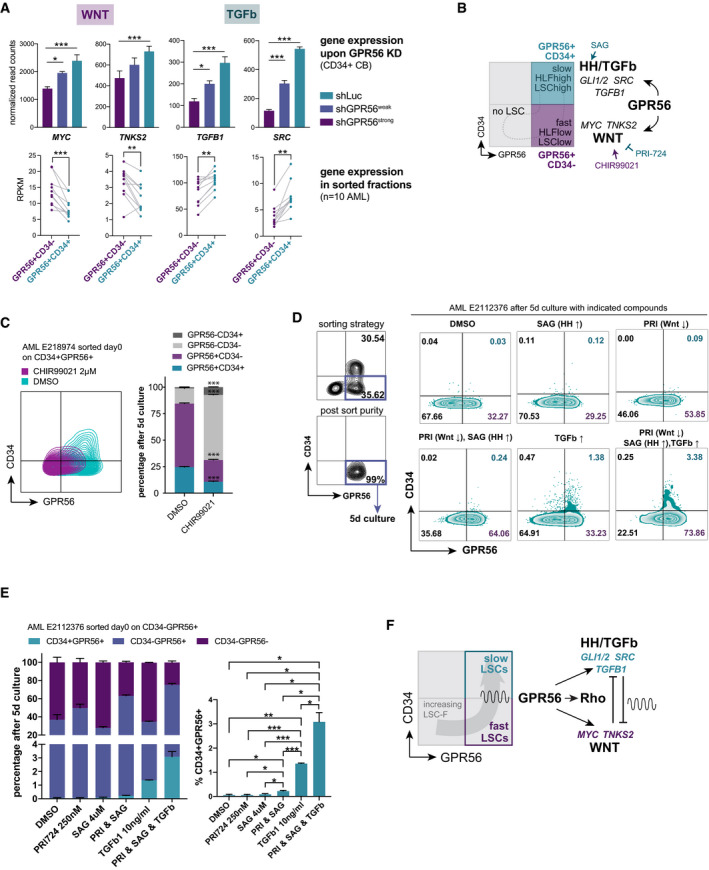
TGFb, HH, and Wnt pathway activities determine the balance between the GPR56^+^CD34^+^ and GPR56^+^CD34^−^ LSC compartments RNA‐seq after GPR56 KD in CD34^+^ CB cells reveals that GPR56 enhances gene expression displayed as reads per kilobase per million mapped reads (RPKM) of *MYC, TNKS2, TGFB1,* and *SRC* (top). Previously published RNA‐seq of ten sorted CD34^+^GPR56^+^ versus CD34^−^GPR56^+^ fractions (Garg *et al*, 2019) shows a divergent expression pattern: downregulation of Wnt targets *MYC* and *TNKS*, but upregulation of *TGFB1* and *SRC* in the CD34^+^GPR56^+^ fraction. Unpaired *t*‐test, bars, and error bars represent mean and SD of three biological replicates, ***P < 0.0005, **P < 0.005, *P < 0.05.Cartoon visualizing that GPR56 enhances genes and pathways differentially active in the CD34^+^GPR56^+^ fraction, which is characterized by slow cell cycle progression, high LSC frequency, and high expression of the stemness gene *HLF* versus the CD34^−^GPR56^+^ cells, which are more differentiated (lower LSC frequency), cycle faster, and have little *HLF* expression. Arrows and blocked arrows indicate activation or inhibition by the indicated small molecules, respectively, which were used in subsequent experiments.Contour FACS plots (left) and summary bar graph (right) showing CD34 and GPR56 expression after 5‐day culture of purified CD34^+^GPR56^+^ cells from AML sample E218974 with CHIR99021 or vehicle DMSO, Unpaired *t*‐test, bars, and error bars represent mean and SD of three technical replicates, ***P < 0.0005.
*Left*: FACS profile and sorting strategy for the CD34^‐^GPR56^+^ fraction (top), and post‐sort purity (bottom) of AML sample E2112376 on the day of thawing. *Right*: CD34 and GPR56 expression measured by flow cytometry 5 days post exposure of purified CD34^‐^GPR56^+^cells to the indicated molecules or their combinations. PRI: PRI‐724, arrows indicate whether the pathway is inhibited (↓) or activated (↑) by the compound.
*Left*: Bar graph showing the distribution of CD34 and GPR56 fractions at the end of the 5‐day culture of purified CD34^−^GPR56^+^ cells from AML E2112376 with indicated compounds. *Right*: statistical analysis shown only for the CD34^+^GPR56^+^ fraction. See Dataset EV11 for full statistical analysis. PRI: PRI‐724, Unpaired *t*‐test, bars, and error bars represent mean and SD of three individual treatments, ***P < 0.0005, **P < 0.005, *P < 0.05.Cartoon visualizing the proposed mechanism by which both GPR56^+^ LSC‐enriched compartments are maintained: GPR56 enhances pathways, which reciprocally inhibit each other and are differentially active in the two fractions. This should result in a constant transition between the compartments and thus prevent exhaustion of the two populations.

Wnt, Hh, and EMT regulators had been associated with self‐renewal in AML before (Dierks *et al*, 2008; Wang *et al*, 2010; Carmichael *et al*, 2020). To better understand why GPR56 enhances all three processes in parallel, we compared the GPR56 knockdown RNA‐seq results with our previously published gene expression study comparing ten sorted CD34^+^GPR56^+^ and CD34^‐^GPR56^+^ LSC compartments, which we showed before contain slowly versus more rapidly cycling LSCs, respectively (Pabst *et al*, 2016; Garg *et al*, 2019). This combined analysis revealed that key drivers of the three networks, which are all enhanced by GPR56, have divergent expression patterns in the two LSC compartments: While *TGFB1, SRC,* and the Hh targets *GLI1/GLI2* are overexpressed in the CD34^+^GPR56^+^ fraction, the Wnt targets *MYC* and *TNKS2* are more highly expressed in the CD34^−^GPR56^+^ compartment (Fig 4A, Appendix Fig S4C, summarized in Fig 4B). To gain insight into the mechanism that maintains the differential expression patterns in the two GPR56^+^ compartments, we used small molecule inhibitors and agonists to modulate the pathways in primary bulk and sorted AML cells. We found that Wnt/β‐catenin/CBP inhibition by the molecules PRI‐724 or iCRT3 increased the phenotypic CD34^+^GPR56^+^ LSC fraction in the PDX sample AML‐661 (Appendix Fig S4B).

Conversely, the Wnt agonist CHIR99021 or exposure to Wnt3a conditioned media caused a significant loss of this compartment (Appendix Fig S4B and C). In line, PRI‐724 increased *SRC*, *TGFB1*, and *SNAI1* mRNA expression in AML‐661 (Appendix Fig S4D).

In further support of our hypothesis that Wnt antagonizes EMT associated genes in AML, we found that *TGFB1*, *TWIST1*, and *SNAIL1* were exclusively suppressed by the Wnt/AhR agonist 6‐BIO, but not by the pure AhR agonist MeBIO, indicating that the suppression was predominantly caused by the Wnt agonist activity of 6‐BIO and not by AhR activation (Appendix Fig S4E).

To test whether modulation of the different pathways caused a real reciprocal shift between the compartments rather than enrichment of one over the other fraction, we performed several sorting experiments. When sorted CD34^+^GPR56^+^ cells from AML E218974 were stimulated with CHIR99021 for 5 days, the CD34^+^GPR56^+^ fraction was significantly reduced compared with the differentiation that naturally occurs in standard culture conditions (Fig 4C). Next, we sorted CD34^+^GPR56^+^ and CD34^‐^GPR56^+^ cells from two other primary AML samples and cultured the cells with Wnt antagonist PRI‐724, Hh agonist SAG, and TGFβ. The combination of all three molecules was most efficient in maintaining GPR56 expression during the 5‐day culture and even generated CD34^+^GPR56^+^ from the sorted CD34^‐^GPR56^+^ cells (Fig 4E, Appendix Fig 4F and G, Dataset EV11).

Together, these results suggest a scenario, in which the Wnt‐enhancing activity of GPR56 in the CD34^+^GPR56^+^ compartment supports the transition to the more differentiated and more rapidly cycling *MYC* ^high^ *TGFB1* ^low^ GPR56^+^CD34^‐^ compartment. In turn, suppression of Wnt activity and activation of SMO/Hh‐ and EMT‐associated genes replenish the slowly proliferative *MYC* ^low^ *TGFB1* ^high^ CD34^+^ fraction (scenario outlined in Fig 4F). This model provides an explanation why specific Wnt and Hh inhibitors are inefficient to permanently eradicate AML (Jiang *et al*, 2018; Cortes *et al*, 2019). Instead, it suggests that small molecules have to act more upstream, e.g., on the level of GPR56, to eradicate LSCs.

### The CDK7/12/13 inhibitor THZ1 suppresses both GPR56^+^ LSC compartments *in vitro* and *in vivo*

**Figure 5:**
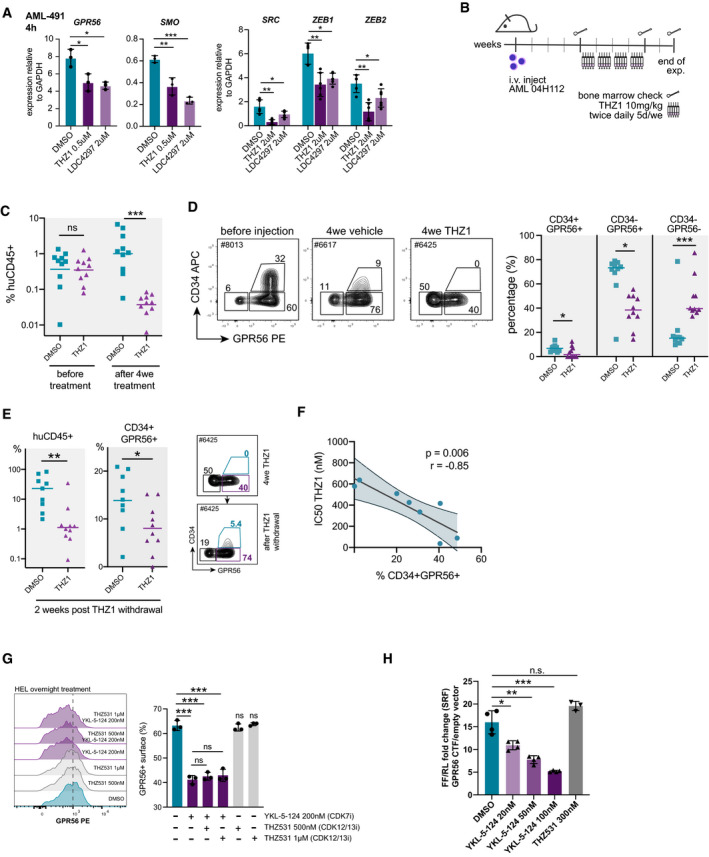
CDK7/12/13 inhibitors suppress the GPR56^+^ LSC compartment *in vitro* and *in vivo* Gene expression of *GPR56*, *SMO (left),* and TGFb targets *SRC, ZEB1,* and *ZEB2 (right)* normalized to GAPDH in AML‐491 cells determined by q‐RT‐PCR 4 h after treatment with THZ1 0.5 µM or LDC4297 2 µM. Unpaired *t*‐test. Mean, SD, and individual values from three individual treatments. ***P < 0.0005, **P < 0.005, *P < 0.05.Setup of *in vivo* drug treatment. NSG mice were injected with 10^5^ AML 04H112 cells. Four weeks post injection, bone marrow (BM) was analyzed for human leukemic engraftment by BM aspiration. Treatment with either THZ1 or vehicle was started in the following week as indicated. BM was analyzed again after the end of the 4‐week treatment period.Overall percentage of human (huCD45^+^) leukemic cells in mice before and at the end of the 4‐week treatment period. Individual mice and mean engraftment are shown. Unpaired *t*‐test. ***P < 0.0005. *N* = 10 mice for each group.
*Left*: Representative FACS plots showing the typical CD34^low^GPR56^high^ profile of sample 04H112 before injection and after the 4‐week treatment with THZ1. *Right*: both LSC compartments, the CD34^‐^GPR56^+^ and the CD34^+^GPR56^+^ fractions were significantly reduced *in vivo* in the THZ1 treatment group. Individual mice and mean engraftment are shown. Unpaired *t*‐test. ***P < 0.0005, **P < 0.005, *P < 0.05. *N* = 10 mice for each group.
*Left*: Overall percentage of human (huCD45^+^) leukemic cells and the fraction of CD34^+^GPR56^+^ among human cells (mean, individual mice) two weeks after drug withdrawal. *Right*: FACS plot visualizing that the CD34^+^GPR56^+^ is re‐established upon drug removal. Unpaired *t*‐test, ***P < 0.0005, **P < 0.005, *P < 0.05.Correlation plot showing an anti‐correlation between the percentage of CD34^+^GPR56^+^ cells in AML samples and the corresponding IC50s for THZ1 determined in the respective samples. Pearson correlation. Each dot represents one sample. See Dataset EV12 for sample characteristics.FACS histogram plot (left) and summary bar graph from three replicate wells (right) showing reduction of GPR56 surface expression on the GPR56‐positive AML cell line HEL only in conditions that contain the more specific CDK7 inhibitor YKL‐5‐124, but not those that contain only the specific CDK12/13 inhibitor THZ531. Unpaired *t*‐test, bars, and error bars represent mean and SD of three individual treatments, ***P < 0.0005, **P < 0.005, *P < 0.05.SRF reporter assay showing dose‐dependent suppression of the GPR56‐CTF‐induced SRF signal by the CDK7 inhibitor YKL‐5‐124, but not by the CDK12/13 inhibitor THZ531. Four technical replicates. One of the four individual experiments. Unpaired *t*‐test, bars and error bars represent mean and SD, ***P < 0.0005, **P < 0.005, *P < 0.05.

The search for small molecules that might act upstream of the GPR56‐associated network led us to the CDK7/12/13 inhibitor THZ1, as it had been shown to overcome resistance to SMO antagonists in medulloblastoma by acting upstream and independent of SMO (Liu *et al*, 2019). We therefore tested its activity in primary AML cells and found that it transcriptionally repressed *GPR56*, as well as Hh, Wnt, and EMT‐associated genes (Appendix Fig S5A–C). The CDK7 inhibitor (CDK7i) LDC4297 caused similar, but weaker effects, and had higher half‐inhibitory concentrations (IC50) in different AML cell lines and primary AML cells (Appendix Fig S5D). We therefore used THZ1 for *in vivo* treatment of a GPR56^high^ human PDX AML sample (04H112) and found that the molecule significantly suppressed leukemia development during the four‐week treatment period (Fig 5B and C). Immunophenotyping of the human cells engrafted in NSG mice revealed a significant reduction of both GPR56^+^ LSC‐enriched compartments (Fig 5D). These effects were still visible after two‐week drug withdrawal (Fig 5E). However, the CD34^+^GPR56^+^ compartment was replenished in some mice in which this compartment was completely suppressed before drug withdrawal, suggesting that the CD34^+^GPR56^+^ fraction was specifically vulnerable to THZ1. In support of this, there was an anti‐correlation between the CD34^+^GPR56^+^ percentage in eight normal karyotype AML specimens and corresponding half‐inhibitory concentrations (IC50) for THZ1, suggesting that THZ1 affected pathways active in this compartment more than pathways in other fractions (Fig 5F). To dissect whether the activity of THZ1 against the GPR56^+^ compartment was caused by CDK7 or CDK12/13 inhibition, we treated the GPR56^+^ AML cell line HEL (Fig 5G, Appendix Fig 5E) and healthy CB CD34^+^ cells (Appendix Fig 5F) with specific CDK7i (YKL‐5‐124) and CDK12/13i (THZ531) (Kwiatkowski *et al*, 2014; Zeng *et al*, 2018; Olson *et al*, 2019). These experiments revealed that only CDK7i, but not CDK12/13i, suppressed GPR56 in these cell types. Further characterization of the compounds revealed that only CDK7i (THZ1, YKL‐5‐124), but not CDK12/13i (THZ531), suppressed the GPR56 CTF‐induced SRF signal in the luciferase reporter assay (Fig 5H, Appendix Fig 5G), while only THZ1 and THZ531, but not YKL‐5‐124, suppressed the baseline and GPR56‐enhanced Wnt reporter signals (Appendix Fig 5H).

These *in vivo* and *in vitro* results suggested that additional compounds are required to more efficiently eradicate both LSC compartments.

### CDK7 inhibition synergizes with the Bcl‐2 inhibitor venetoclax *in vitro* and *in vivo*

**Figure 6:**
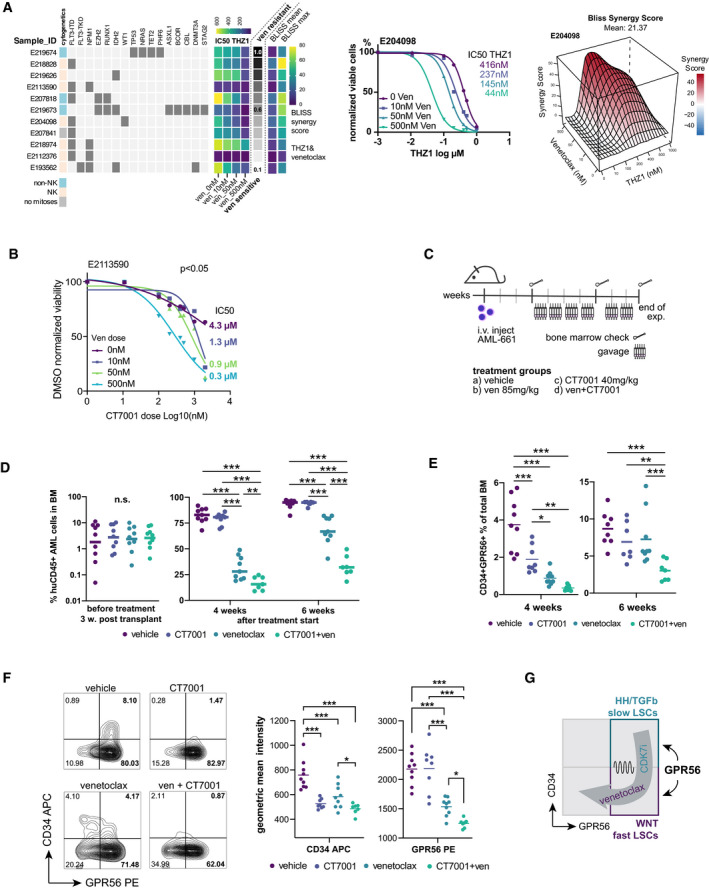
Synergism of CDK7 inhibitors with venetoclax *Left:* Eleven primary AML specimens with different cytogenetic and molecular genetic aberrations (see Dataset EV12 for details) were treated with THZ1 or YKL‐5‐124 alone or in combination with venetoclax. IC50 values in presence of increasing doses of venetoclax are visualized for THZ1. The gray‐scale bar indicates the fraction of viable cells in presence of a high dose of venetoclax (500 nM) compared with DMSO to provide an approximation whether a sample was rather sensitive or resistant against venetoclax alone. Note that the addition of venetoclax reduces the IC50 in all samples regardless of their baseline resistance against venetoclax alone. The last four columns provide the average and maximum BLISS scores reached by a combination of THZ1 and venetoclax (*N* = 11) *Middle*: representative dose‐response curves of THZ1 in presence of increasing doses of venetoclax for one primary AML specimen with intermediate venetoclax sensitivity. *Right:* representative 3D‐synergy plot showing the BLISS synergy scores at indicated concentrations of THZ1 and venetoclax for AML E204098.Dose‐response curve for the CDK7i CT7001 in presence of increasing doses of venetoclax for primary AML E2113590. The indicated doses on the right represent the IC50 dose.Schematic visualizing the setup for the combinatory *in vivo* treatment. AML‐661 cells were injected in NSG mice. Bones indicate the timepoints of bone marrow aspirations to monitor engraftment of human leukemic cells in mice before treatment start and during treatment.Percentage of human CD45^+^ leukemic cells three weeks post injection, i.e. before treatment start (left), and 4 and 6 weeks after treatment start (right). Dots represent individual mice, horizontal lines represent means. Unpaired *t*‐test, ***P < 0.0005, **P < 0.005.Percentage of human CD34^+^GPR56^+^ cells in the mouse bone marrow at 4 and 6 weeks post treatment start with the indicated compounds. Dots represent individual mice, horizontal lines represent means. Unpaired *t*‐test, ***P < 0.0005, **P < 0.005, *P < 0.05.
*Left*: Representative FACS plots showing CD34 and GPR56 expression on AML cells after 4‐week treatment with the indicated compounds. *Right*: Statistical analysis of the geometric mean intensity of CD34 APC (left) and GPR56 PE (right) in the four treatment groups. Horizontal lines represent means. Unpaired *t*‐test, ***P < 0.0005, **P < 0.005, *P < 0.05.Cartoon visualizing how CDK7i and venetoclax synergize to suppress both GPR56^+^ compartments in AML.

As previous studies had already revealed a synergistic effect of THZ1 with venetoclax in other cancers (Cayrol *et al*, 2017), we tested the synergistic effect of THZ1 and the more specific CDK7i YKL‐5‐124 alone and in combination with 10 nM, 50 nM, and 500 nM of venetoclax in eleven primary AML samples (Fig 6A, Dataset EV12, Appendix Fig 6A). These included samples of different genetic groups, as well as patients refractory to salvage chemotherapies such as 5‐azacytidin with venetoclax, or quizartinib with high‐dose cytarabine and mitoxantrone. Samples not reaching a half‐maximum cell reduction at 500 nM venetoclax were considered venetoclax‐resistant. Normalized IC50s for THZ1 were significantly lower with increasing concentrations of venetoclax (Fig 6A, Dataset EV12). As synergism between drugs is not well reflected by individual IC50s, we used the R package ‘synergyfinder’ (He *et al*, 2018) to calculate different synergy scores including BLISS and ZIP scores (Bliss, 1939; Yadav *et al*, 2015; Fig 6A; Dataset EV12). There was in general strong synergism with venetoclax, however, BLISS scores for THZ1 were higher compared with the specific CDK7i YKL‐5‐124 in most samples. This might be because of the strong MCL‐1 suppressive activity of THZ1, which is also observed with CDK12/13i, but which is weaker or absent with CDK7i and not recapitulated by GPR56 KD (Appendix Fig 6B–D).

Because of the pleiotropic effects of THZ1 including very strong MCL1 suppression, more specific CDK7i have been developed for clinical trials. To assess synergism of CDK7i and venetoclax *in vivo,* we therefore selected the more specific CDK7i CT7001, an orally active drug, which has entered clinical trials for solid cancers (NCT03363893), after confirming synergism with venetoclax *in vitro* in two different primary AML samples (Fig 6B, Appendix Fig 6E). The combinatory treatment of CT7001 and venetoclax significantly reduced AML expansion more than venetoclax alone (Fig 6C and D). The low efficacy of CT7001 as monotherapy might be because of the low dosage applied by gavage to reduce side effects (40 mg/kg instead of 100 mg/kg as reported elsewhere (Clark *et al*, 2017)). While both drugs as monotherapy significantly reduced the CD34^+^GPR56^+^ compartment within total mouse bone marrow when assessed at the end of the 4‐week treatment, only the combination therapy was able to significantly suppress the CD34^+^GPR56^+^ compartment by the end of the experiment (Fig 6E). FACS profiles assessed at four weeks confirmed that CT7001 affected the CD34^+^GPR56^+^ more than the CD34^‐^GPR56^+^ fraction similar to what we had observed with THZ1 (Fig 5D), while venetoclax significantly suppressed overall GPR56^+^ expression (Fig 6F).

In summary, our data suggest that combined CDK7i/venetoclax treatment might represent an efficient strategy to suppress both GPR56^+^ LSC compartments in AML (Fig 6G).

## Discussion

The rationale for applying ATAC‐seq profiling to primary human AML samples was the aim to detect subtle changes in lowly abundant TF activities that might not be detectable by RNA‐seq to identify the network underlying the very poor outcome of AML with high GPR56 expression (Pabst *et al*, 2016; Garg *et al*, 2019) including TFs upstream of GPR56. Our combined ATAC‐/RNA‐seq approach pointed towards EMT regulators, Wnt, Hh, and Rho signaling. Although these pathways have been linked to self‐renewal in AML before, targeting them with small molecule inhibitors has not been proven sufficient to permanently eradicate the disease neither in pre‐clinical models (Fukushima *et al*, 2016; Jiang *et al*, 2018), nor in the clinical setting (e.g. FDA approved Hh inhibitor glasdegib; Savona *et al*, 2018; Norsworthy *et al*, 2019). The inability of these inhibitors to induce durable complete remissions raises the question why targeting *bona fide* self‐renewal pathways seems to rather have transient effects on LSCs, but fails to eliminate them. Here, we propose a model that provides an explanation on how LSCs might escape pathway‐specific LSC‐directed therapies while attributing GPR56 a pivotal role in this process.

We first dissected in individual functional assays including luciferase reporter assays, IF imaging, and xenotransplantation that GPR56 is functionally important for engraftment of human AML cells in immunocompromised mice and activates not only RhoA signaling, as described before (Piao *et al*, 2004; Singer *et al*, 2013; Kishore *et al*, 2016), but also regulates Wnt and Hh signaling, and EMT‐associated gene expression. In particular, our mechanistic studies revealed that GPR56 enhances Wnt signaling in dependence of the co‐receptor LRP6. Another example of an aGPCR that modulates Wnt signaling at the co‐receptor level is GPR124, which facilitates binding of Wnt7 to Frizzled and thereby promotes Wnt ligand‐specific effects (Eubelen *et al*, 2018). Moreover, we identified *ITF2/TCF4*, a basic helix‐loop‐helix TF, which is induced by Wnt/β‐catenin to promote cancer growth (Kolligs *et al*, 2002), as upstream regulator of GPR56, suggesting positive feedback between GPR56 and Wnt. A similar loop was found for SMO/Hh in a way that GPR56 KD suppressed SMO mRNA and RFX2 target genes, while the GPR56 promoter in turn was shown before to be bound by several *RFX* TFs (Bae *et al*, 2014). Moreover, the EMT regulator *SNAI1* was identified upstream of GPR56, while GPR56 itself positively regulated *TGFB1,* the key driver of EMT, and *SRC*, which is required for TGFB1 induced EMT (Galliher & Schiemann, 2006).

Despite these positive feedback loops, we found that the Wnt pathway seems to play a crucial antagonizing role in this network, raising the question why GPR56 concomitantly enhances pathways that inhibit each other. The principle of reciprocal inhibition is known from the neuronal system as the basis of continuous neuronal oscillation (Friesen, 1994), and antagonism of Wnt and Hh has also been observed in neurogenesis (Joksimovic *et al*, 2009). Integration of our novel functional data presented here with our previous gene expression studies (Garg *et al*, 2019) and sorting experiments (Pabst *et al*, 2016) finally suggests a scenario, in which GPR56 ensures the constant supply of LSC‐enriched compartments with different biological features: the CD34^+^GPR56^+^ fraction characterized by high TGFb/Hh activity, low WNT activity, and slow cell cycle progression (Hh/TGFb^on^WNT^off^), and the more rapidly expanding, CD34*^‐^* GPR56^+^ compartment characterized by lower LSC frequency, low Hh/TGFb activity, and high WNT activity (Hh/TGFb^off^WNT^on^). Evidence for reciprocal transition between the two LSC compartments exists: we previously showed that CD34^+^GPR56^+^ cells expanded more slowly than CD34^‐^GPR56^+^ cells and were capable of generating all other fractions *in vitro* and *in vivo* (Pabst *et al*, 2016). Here, we provide evidence that the CD34^+^GPR56^+^ compartment can be regenerated by CD34^‐^ cells from sorted *in vitro* cells and also in mice, in which the CD34^+^GPR56^+^ compartment was completely depleted during THZ1 treatment, further corroborating that the two GPR56^+^ LSC compartments can replenish each other. In line with this scenario, GPR56‐regulated genes comprised *CCNE1*, which has been described to enable quiescent cells to re‐enter cell cycle (Campaner *et al*, 2013).

Reciprocal transition between slowly and rapidly cycling GPR56^+^ LSCs would also explain how GPR56^high^ AML escapes standard chemotherapy treatment. Moreover, the interactions of multiple signaling pathways suggest that inhibiting one of these pathways individually, e.g. as tried with Hh inhibitors will be insufficient to fully block the GPR56‐associated network. Our data suggest that combined CDK7/12/13 inhibitors such as THZ1 might be required to overcome these issues. As observed in other cancers (Cayrol *et al*, 2017), THZ1 synergized with the Bcl‐2 inhibitor venetoclax most likely in part by suppressing MCL‐1. However, we also observed synergism of venetoclax with more specific CDK7i *in vitro* and *in vivo*, which had no or only a weak effect on MCL‐1 protein levels suggesting that the synergism between CDK7i and venetoclax was mediated also by other mechanisms than MCL‐1 suppression. The precise molecular processes underlying synergism of CDK7i and venetoclax will have to be addressed in future studies. Importantly, synergism of THZ1 and the more specific CDK7i YKL‐5‐124 occurred also in samples, which were highly resistant to venetoclax alone, and also in FLT3‐ITD samples from patients, who were refractory to FLT3‐inhibitors, and might therefore offer additional benefits compared to currently available venetoclax ‐based combination regimens. Together, these results propose combinatorial treatment of venetoclax with CDK7i as promising therapeutic approach to suppress the GPR56^+^ associated network in AML.

## Materials and Methods

### Patient and cord blood samples

Patient samples and cord blood units were collected after obtaining written informed consent in accordance with the Declaration of Helsinki and the Department of Health and Human Services Belmont Report. Cryopreserved AML patient samples were provided by the Medical Department V, Heidelberg University Hospital, Germany, the Department of Internal Medicine I, University Hospital of Dresden Carl Gustav Carus, Germany, the Department of Internal Medicine III, Ludwig‐Maximilians‐University, Munich, Germany, and the Leukemia Cell Bank of Quebec, Maisonneuve‐Rosemont Hospital, Montreal, Canada. Samples were provided according to ethically approved protocols by several biobanks. The project was approved by the Research Ethics Board of the Medical Faculty of Heidelberg University. Cord blood units were collected after obtaining written informed consent at the CHU Sainte‐Justine, Montreal, Canada, and the Department of Obstetrics at University Hospital Heidelberg, Germany. Patient‐derived xenograft AML‐491 and AML‐661 cells were generated as described before (Vick *et al*, 2015) and were kindly provided by I. Jeremias and B. Vick.

### *In vivo* experiments

NOD. Cg‐Prkdc^scid^Il2rg^tm1^Wjl (NSG) and NOD. Rag1‐; γcnull‐SGM3 (NRGS) mice were purchased from Jackson Laboratories. NOD. Cg‐*Kit^W‐41J^ Prkdc^scid^ Il2rg^tm1Wjl^*/WaskJ (NSGW41) mice were kindly provided by Dr. Claudia Waskow (Cosgun *et al*, 2014). Female or male mice aged 6–20 weeks were used in our study. They were bred and housed in the specific pathogen‐free animal facility at the German Cancer Research Center (DKFZ), Heidelberg. All animal experiments were approved and performed in accordance with the regulatory guidelines of the official committee (Regierungspräsidium Karlsruhe). For xenotransplantation assays, cells were injected in the mice via the tail vein after sublethal irradiation (1.75 Gy for NSG, 2 × 2.5 Gy for NRGS). Engraftment levels were analyzed at indicated time points by bone marrow aspiration, and total marrow was analyzed at final sacrifice. For CB CD34^+^ experiments, cells were injected after overnight transduction, and gene transfer was determined by flow cytometry in the remaining cultured cells 48 h later. For Fig 2E, 5 × 10^5^ and 1 × 10^5^ transduced AML‐491 cells were injected in NRGS and NSGW41 mice, respectively, after overnight transduction. Gene transfer was determined by flow cytometry 72 h post transduction. For the THZ1 *in vivo* treatment, 1 × 10^5^ patient‐derived xenograft (PDX‐04H112) AML cells were suspended in 200 μl phosphate buffer solution and injected intravenously (i.v.) in NSG mice. Successful engraftment levels were confirmed at week 4 post injection. Intraperitoneal (i.p.) treatment was started subsequently with either vehicle (1% DMSO + 20% PEG300 + 30% corn oil in water) or THZ1 (10 mg/kg, twice daily, Monday to Friday) for 4 weeks. For the CT7001 and venetoclax *in vivo* treatment, 1 × 10^5^ patient‐derived xenograft PDX AML‐661 cells were injected intravenously (i.v.) in sublethally irradiated NSG mice. At 3 weeks post injection, mice were randomly sorted into 4 treatment groups: vehicle (1% DMSO + 20% PEG300 + 30% corn oil in water), 85 mg/kg venetoclax, 40 mg/kg CT7001, or a combination of 85 mg/kg venetoclax and 40 mg/kg CT7001. Mice were dosed orally, once daily, 5 days a week, for 4 weeks. Bone marrow was analyzed by aspiration from one femur in the following week, and treatment was restarted for another two weeks, after which bone marrow was analyzed again.

### RNA‐and ATAC‐seq analyses

#### ATAC‐seq library preparation details

We followed the Omni‐ATAC method outlined previously (Buenrostro et al, 2015) for sample preparation. The library was optimized for enrichment for 100–1,000 bp fragments using SPRI beads based size‐selection, and the quality of the purified DNA library was analyzed on a Bioanalyzer (2100 Expert software, Agilent Technologies) using High Sensitivity DNA Chips (Agilent Technologies Inc #5067‐4626). The appropriate concentration of sample was determined using the Qubit Fluorometer (Molecular Probes). Ten 40 nM samples were pooled and run on a NextSeq 500/550 High Output Kit (Illumina, Inc. San Diego, CA #20024907) and the NextSeq 500 Illumina Sequencer to obtain paired end reads of 75 bp.

#### ATAC‐seq data processing

The processing of ATAC‐seq data has been described in detail in a previous study by (Berest *et al*, 2019). Briefly, we used an in‐house Snakemake (Köster & Rahmann, 2012) pipeline that starts with raw fastq files and integrates multiple steps for quality control (*FastQC*), adaptor trimming (*Trimmomatic* (Bolger *et al*, 2014)), alignment (*Bowtie2*; Langmead & Salzberg, 2012), as well as general and ATAC‐Seq specific post‐alignment filtering and processing steps (Berest *et al*, 2019). Noteworthy, the filtering steps include: (i) removing mitochondrial reads and reads from non‐assembled contigs or alternative haplotypes, (ii) filtering reads with a mapping quality below 10, (iii) marking and removing duplicate reads with *MarkDuplicates* from the Picard toolset, (iv) adjusting read start sites as described previously (Buenrostro *et al*, 2013); and (v) removing reads with insertions or deletions using *samtools*. Finally, peak calling is done with MACS2 (parameters: ‐q 0.01 ‐g hg19 ‐‐nomodel ‐‐keep‐dup all) followed by filtering using *bedtools subtract* against the publicly available blacklist regions. Last, for quality control, we also obtained various summary statistics and additional files and plots (e.g., coverage files for visualization, transcription start site enrichment, sample‐specific fragment length distributions, library complexity measures, PCA, sample correlations).

#### Differential peak analysis and consensus peak generation

To identify differentially bound peaks between GPR56^high^ and GPR56^low^ samples, we used the *DiffBind* Bioconductor R package (Ross‐Innes *et al*, 2012) with the mutational status as a blocking factor. Consensus peaks were generated with the function *dba.peakset* using the parameter *minOverlap* to define the number of samples within which a peak should be present based on all 35 samples, therefore, also including the GPR56^middle^ ones that were not used further subsequently except for consensus peak generation. We used different values for *minOverlap* and generated a consensus peakset out of the individual peak files as generated by the ATAC‐seq pipeline as outlined above for values of 1, 10, 15, and 30. The consensus peakset based on minOverlap = 10 consists of 109,803 (used as default for all subsequent analyses), for minOverlap = 1 247,442 (used as background for the *GREAT* analysis), for minOverlap = 15 87,410 and for minOverlap = 30 32,406 peaks.

#### ATAC‐seq PCA

We performed a principal‐component analysis (PCA) based on the top 500 variable peaks from the consensus peaks (based on the variance using *rowVars*) based on the variance‐stabilized data (see below for details). To summarize and quantify the contribution of each covariate to the overall variability, we then ran a linear regression for each covariate and each of the first 5 PCs and extracted the adjusted *R*‐squared value.

#### Enrichment analysis of differential ATAC‐seq peaks

For annotating the differentially bound peaks, we used multiple methods. First, we used three different consensus peaksets (based on minOverlap = 1, 10, and 30) as well as the differentially bound peaks only and overlapped them with known annotation categories (e.g., promoter, downstream, intragenic, UTR, etc) with the *annotatePeak* function of the *ChIPseeker* R/Bioconductor package using the default parameters. For overlaps with the enhancer annotation, we used the human data from the EnhancerAtlas 2.0 (http://www.enhanceratlas.org/; Gao & Qian, 2020).

Last, we also used *GREAT* (McLean *et al*, 2010) v3 via the *rGREAT* Bioconductor package with the consensus peakset based on minOverlap = 1 (see above) as background and as foreground all differentially bound peaks (FDR < 0.05) with either a positive or a negative fold‐change.

#### Differential TF activity analysis

We ran *diffTF* (Berest *et al*, 2019) in permutation mode with 1,000 permutations for 640 TFs based on *in silico* predicted TFBS (using *PWMScan*, see (Berest *et al*, 2019) for full details) using TF‐binding models from the *HOCOMOCO* v10 database (Kulakovskiy *et al*, 2013) with the consensus peakset for minOverlap = 10 as described above and the same design as for *DiffBind* (i.e., including the mutational status as covariate).

#### GPR56 KD RNA‐seq experiment

Frozen cord blood (CB) MNC from two female donors were thawed, CD34^+^ cells were isolated by magnetic bead separation as described above, pooled, and transduced with lentiviral particles at multiplicity of infection (MOI) 30 after 48 h pre‐stimulation in culture media at a density of 150,000 cells per well in a 12‐well plate, 1 ml volume, 2 individual infections per condition. Cells were washed on the next day and sorted for GFP positivity 72 h post infection. Cells were resuspended in Trizol for RNA isolation directly after sorting, and RNA was isolated according to the manufacturer's instructions followed by an additional purification step on RNeasy Mini columns. High RNA quality was confirmed by nanodrop and bioanalyzer (RIN between 9.8 and 10). Ovation human FFPE RNA‐Seq Library Systems (Nugen) was used for library preparation. Libraries were prepared using random and poly(T) priming, and unstranded, rRNA‐depleted cDNA libraries were sequenced on an Illumina NextSeq platform.

#### GPR56 KD RNA‐seq data processing

We first performed initial quality control before and after adaptor trimming (*cutadapt*, *‐m 30*) (Martin, 2011) with *FastQC*. We then aligned the samples to hg38 using *STAR* (Dobin *et al*, 2013) with the parameters *‐‐alignSJoverhangMin 8 ‐‐alignSJDBoverhangMin 1 ‐‐alignMatesGapMax 1000000 ‐‐alignIntronMin 20 ‐‐alignIntronMax 1000000 ‐‐outFilterType BySJout ‐‐outFilterMultimapNmax 20 ‐‐outFilterMismatchNmax 999 ‐‐outFilterMismatchNoverReadLmax 0.04*. For gene annotation, we used the Gencode (Harrow *et al*., 2012) v28 annotation. We then quantified gene counts using *featureCounts* from the *Subread* (Liao *et al*, 2014) package with the parameters *‐p ‐B ‐C ‐Q 10 ‐O ‐s 2 ‐t exon ‐g gene_id*. For differential expression, we employed *DESeq2* (Love *et al*, 2014) with the design formula “condition,” and size factor normalization to compare both the weak and the strong KD versus control. Finally, we computed and subsequently used the shrunken log_2_ fold‐changes via *lfcShrink* from *DESeq2* (Love *et al*, 2014) with the *apeglm* (Zhu *et al*, 2019) method.

#### Visualization of ATAC‐seq and RNA‐seq data

For all heatmaps as well as the PCA, we variance‐stabilized the shown data using the *vst* function from *DESeq2*.

### Cell culture

Frozen cryotubes were briefly thawed in a 37°C water bath and resuspended in warm thawing media containing Iscove´s modified Dulbecco´s medium (IMDM) (Thermo Scientific #21980065) supplemented with 20% Fetal bovine serum (FBS) (Sigma #F7524) and 100 µg/ml DNase I (Sigma #DN25). Primary AML cells were cultured in IMDM supplemented with 15% BIT (bovine serum albumin, insulin, transferrin, Stem Cell Technologies #09500), SCF 100 ng/ml (Shenandoah #100‐04), FLT3L 50 ng/ml (Shenandoah #100‐21), IL‐3 20 ng/ml (Shenandoah #100‐80), G‐CSF 20 ng/ml (Shenandoah #100‐72), β‐mercaptoethanol (10^−4^ M), Gentamicin (50 µg/ml), and Ciprofloxacin (10 µg/ml). Fresh cord blood samples were subjected to mononuclear cell (CB‐MNCs) isolation by Ficoll Hypaque (Thermo # GE17‐1440‐02) density gradient. CD34^+^ cells were isolated from CB using MACS microbeads (Miltenyi #130‐100‐453) for 1–2 rounds and checked for CD34^+^ purity by FACS. CD34^+^ cells were cultured in IMDM supplemented with 20% BIT (Stem Cell Technologies #09500), SCF 100 ng/ml (Miltenyi #130‐096‐695), FLT3L 100 ng/ml (Miltenyi #130‐096‐479), TPO 50 ng/ml (Miltenyi #130‐095‐752), β‐mercaptoethanol 10^−4^ M (Gibco #21985023), Gentamicin 50 µg/ml (Thermo #15750060), and UM171 35 nM (Stem cell technologies #72912). For large‐scale cultures, cells were cultured in T25 flasks (TPP #90026), 6‐well plates (Sarstedt #833.920.500), 12‐well plates (Sarstedt #833.921.300) at a density of 3–5 × 10^5^ per ml. Wnt3a and control conditioned media were generated as described (Willert *et al*, 2003).

### Cell lines

MV4‐11 cells (#ACC 102), OCI‐AML2 (#ACC 99), OCI‐AML3 (#ACC 582), HL‐60 (#ACC 3), Kasumi1 (#ACC 220), K562 (#ACC 10), KG1a (#ACC 421), UCSD‐AML1 (#ACC 691), HEL (#ACC 11), and HNT34 (#ACC 600) were purchased from Leibniz Institute DSMZ‐German Collection of Microorganisms and Cell Cultures, Braunschweig, Germany and cultured according to the company´s recommendations. hTERT‐immortalized retinal pigment epithelial cells (RPE‐1, ATCC CRL‐4000) were maintained in DMEM, 10% FBS prior to serum starvation. RPE‐1 cells carrying murine SMO‐eGFP were generated as described (Viol *et al*, 2020).

### Reporter assays and reporter cell lines

#### Wnt and SRF reporter assays

WT or LRP6 KO (LRP6^−/−^) (Berger *et al*, 2017) HEK293T cells were cultured in DMEM supplemented with 10% FBS. For the SuperTop Wnt reporter assay, cells were plated in 96‐well plates in 3–4 replicates and transfected with 50 ng SuperTop reporter plasmid and 2.5 ng pTK‐Renilla control (Promega) as previously described (Cruciat *et al*, 2010) together with 50 ng of plasmid DNA carrying either shRNA against GPR56 or cDNA of GPR56 FL, GPR56 CTF, or GPR56 CTF mutants (pcDNA3.1) as indicated using TurboFect transfection reagent (Thermo #R0532) following the supplier’s protocol. Cells were stimulated with Wnt3a‐conditioned media overnight one day post transfection when indicated in figures. For detection of Rho pathway activation, the previously described 3DA. Luc SRF reporter system was used together with MAL/MKL1 (Megakaryocytic Acute Leukemia) expression plasmid (Miralles *et al*, 2003) as positive control and pTK‐Renilla (Promega) for normalization. For bioluminescence detection, which was performed 48‐h post transfection, cells were lysed in a 96‐well plate format using 1× passive lysis buffer provided in Dual‐Luciferase Reporter Assay System (Promega #E1910), and 25 μl of lysates were transferred into white 96‐well plates (Thermo #9502887). Firefly and Renilla luminescence were measured in a Tecan Microplate reader (SPARK). To normalize for transfection efficiency, Firefly luciferase activity was divided by Renilla luciferase activity to obtain Relative Luminescence Units (RLU).

#### SMO‐GFP RPE‐1 cell line

Doxycycline‐inducible Tet3G‐Smo‐EGFP RPE cells were generated as described (Viol *et al*, 2020). For GFP detection, 1 × 10^4^ SMO‐GFP RPE cells were plated in a 48‐well plate format followed by lentiviral transduction of GPR56 shRNAs and shLuc on the second day. Seventy‐two hours post‐infection, cells were treated overnight with 5 ng/ml doxycycline with or without 1 μM Smoothened Agonist (SAG) HCl (Selleckchem #S7779). On the next day, cells were trypsinized and analyzed by flow cytometry for GFP expression or by q‐RT‐PCR for gene expression changes.

### Lentivirus production and transduction

The production of high‐titer lentiviral particles was carried out following previously described protocols (Garg *et al*, 2019). In brief, TurboFect transfection reagent (Thermo #R0532) was used to transiently package 7.5 μg lentiviral vector with 15 μg psPAX2 packaging plasmid and 4.5 µg VSV‐G plasmid. psPAX2 was a gift from Didier Trono (Addgene plasmid #12260; http://n2t.net/addgene:12260; RRID:Addgene_12260). HEK293T cells were transfected, cultured in DMEM supplemented with 3% heat‐inactivated FBS, and lentiviral supernatant was harvested 48 and 72 h post transfection. Ultracentrifugation was performed through a 20% sucrose cushion to concentrate viral supernatants using a Sorvall WX Ultra 100 ultracentrifuge for 2 h at 4°C and 29,000 rpm. The high‐titer lentiviral particles were reconstituted in Opti‐MEM and stored at −80°C until use. For transduction of shRNAs, cells were first incubated with protamine sulfate for 30 min, followed by adding lentiviral particles directly into culture media for 12–16 h. Forty‐eight to 72 h post infection, gene transfer was checked using flow cytometry.

### Colony assays

After lentiviral transduction, CB CD34^+^ cells were sorted into methylcellulose (Methocult, Stem Cell Technologies #4035) supplemented with EPO 3 IU/ml (Cedarlane #102‐04‐2000 IU) and gentamicin 50 µg/ml (Thermo #15750060) 72 h post infection and plated at 2 ml per well in 12‐well plates (Sarstedt #833.921.300). Colonies were analyzed 10–14 days after plating using an inverted microscope and 10× and 20× magnification.

### Flow cytometry and cell sorting

Cells were stained using the following antibodies: CD45‐Pacific blue (Biolegend #304029), GPR56‐PE (Biolegend #358204), CD11b‐PECy5 (Biolegend #301308), CD34‐APC (BD biosciences #555824), CD14‐APC‐Cy7 (Biolegend #325620), CD45RA‐PE (BD bioscience # 555489), CD11b‐PECy5 (Biolegend #301308), CD38‐PECy7 (BD biosciences #560677), *in vivo* engraftment levels were analyzed with anti‐human CD45‐APC (BD biosciences #555485), CD33‐PECy5 (BD biosciences #551377) and CD19‐PECy7 (BD biosciences #557835). Cell sorting was performed on BD FACS Aria II. Data were acquired on a BD LSRII or BD Celesta flow cytometer equipped with a High throughput sampler (HTS) device and analyzed using BD FACS Diva 4.0 and Flowjo X (Treestar Inc.) software.

### Quantitative real‐time polymerase chain reaction (q‐RT‐PCR)

Total RNA was extracted using Trizol reagent (Invitrogen/Life Technologies) according to the manufacturer's instructions. cDNA was obtained from total RNA by reverse transcription using M‐MLV RT (Thermo #28025103) in 25 μl reactions. Subsequently, q‐RT‐PCR was performed on a Biorad q‐RT‐PCR machine (CFX96 Touch Real Time PCR detection system). *GAPDH* was used as endogenous control. The q‐RT‐PCR primers used in the study are listed in Appendix Table S1.

### Immunofluorescence and microscopy

The antibodies used were mouse anti‐γ‐tubulin (Sigma #T6557), rabbit polyclonal anti‐Arl13b (Proteintech #17711‐1AP), rabbit polyclonal anti α‐tubulin (MBL #PM054). For cilia analysis, RPE cells were grown on coverslips coated with fibronectin, followed by lentiviral transduction of shRNAs 24 h post plating. Forty‐eight hours after infection, cells were cultured in serum‐free DMEM culture media for 24 h to stimulate cilia growth. Cells were then washed and fixed in 4% paraformaldehyde at 4°C followed by iced methanol for 5 min. After blocking in 0.1% Triton X100/PBS supplied with 1% BSA for 1 h, fixed cells were incubated with indicated primary antibodies (1:200) at 4°C overnight. After washing, cells were incubated with either mouse or rabbit Alexa fluor 488, Alexa fluor 594, and Alexa fluor 647 secondary antibodies (1:500, Thermo). DNA was stained with DAPI. Ciliated cell samples were observed using Axio Observer Z1 fluorescence motorized microscope (Zeiss) equipped with 40× NA 1.4 Plan‐Apochromat oil immersion objective. Raw images were processed in ImageJ.

### Compound testing and calculation of synergism

The CDK12/13 inhibitor THZ531 (Selleckchem #S6595), the CDK7/12/13 inhibitor THZ1 (Selleckchem #S7549), and the CDK7 inhibitors YKL‐5‐124 (Selleckchem #S8863) and LDC4297 (Selleckchem #S7992) were dissolved in DMSO at 50 mM stock concentration and diluted in media so that final DMSO concentration was 0.1% in all conditions. SAG (Selleckchem #S7779) was dissolved in water at 20 mM stock concentration. PRI‐724 (#S8968), CHIR‐99021 (#S2924), iCRT3 (#S8647), and venetoclax (#8048) were purchased from Selleckchem and dissolved in DMSO. For *in vivo* THZ1 treatment, THZ1(#HY‐80013) was purchased from MedChemExpress and dissolved in DMSO at 300 mg/ml stock concentration. For the *in vivo* combinatorial treatment, CT7001 (Chemietek #CT‐CT7001) and venetoclax (Hycultec #HY‐15531) were dissolved in vehicle solution (1% DMSO + 20% PEG300 + 30% corn oil in water).

For IC50 calculation and synergism calculation, cells were plated in 384‐well plates and incubated with the combinations of different drugs for 3–5 days as indicated in figures. Each treatment condition was performed in at least 3 replicates. Cell viability was measured based on propidium iodide (PI) staining using a BD LSRII or BD Celesta flow cytometer equipped with a High throughput sampler (HTS) device. The resulting dose‐response matrix data and Bliss scores were analyzed with the synergyfinder R package (Yadav *et al*, 2015; He *et al*, 2018). IC50s were calculated with GraphPad Prism v09.

### Western blot

For isolation of total lysates, cells were pelleted after 1× PBS wash and lysed in RIPA lysis buffer (Thermo #89900) supplied with protease inhibitor cocktail (Sigma #11836170001). Lysates were collected in new pre‐chilled tubes and protein concentration was measured using Bradford reagent (Bio‐Rad #500‐025) and BSA standards (Bio‐Rad #500‐027). Equal amount of protein was mixed with 4× gel loading dye (Thermo #NP007) and analyzed by pre‐casted gel (Thermo #NP0036) and Western blotting. Final western blots were detected on a GE Healthcare Life Sciences, Amersham Imager 600, followed by analyzing band intensities with ImageJ (U.S. National Institutes of Health, Bethesda, MD, USA).

Antibodies used for western blotting were as follows: anti‐LRP6 (#S2560) primary antibody was purchased from Cell Signaling Technology, antibody against anti‐GAPDH (#GTX627408) was from GeneTex, anti‐Vinculin (#sc‐736914) primary antibody was from Santa Cruz, antibody against β‐catenin (#610153) was from BD Biosciences, anti‐MCL‐1 (#ab32087), anti‐BCL‐2 (#ab692) primary antibodies were from Abcam.

### Cloning procedures

Small hairpin (sh)‐oligos against GPR56, Luciferase, or scrambled were cloned into the plko.1 system (Sigma), in which the puromycin resistance cassette had been replaced by eGFP (pLKO‐U6‐shOligo‐hPGK‐eGFP) or Ametrine (pLKO‐U6‐shOligo‐hPGK‐Ametrine), the shRNA oligos used are listed in Appendix Table S2.

GPR56 full‐length cDNA was obtained from OriGene Technologies (NM_005682.4) and cloned into pcDNA3.1 and the previously described pCCL‐c‐MNDU3‐eGFP backbone (Garg *et al*, 2019). The primers used for generating GPR56 CTF and mutant constructs are listed in Appendix Table S3.

### Statistical analyses

For the RNA‐ and ATAC‐seq analyses, statistical tests are described in each of the relevant analyses and p‐values are given in text or Figures. Individual assays were analyzed with Graph Pad Prism 8 by unpaired *t*‐tests to compare two conditions if not otherwise stated in Figure legends. When multiple groups were compared, *P*‐values were Benjamini Hochberg corrected. Asterisks indicate the following *P*‐value levels if not otherwise specified in the text: *P < 0.05, **P < 0.005, ***P < 0.0005, ****P < 0.0001.

## Author contributions

**Caroline Pabst:** Conceptualization; Formal analysis; Supervision; Funding acquisition; Visualization; Writing—original draft; Writing—review and editing. **Lixiazi He:** Data curation; Formal analysis; Validation; Visualization; Writing—original draft; Writing – review and editing. **Christian Arnold:** Data curation; Formal analysis; Methodology. **Judith Thoma:** Formal analysis; Writing – review and editing. **Christian Rohde:** Software; Formal analysis; Methodology. **Maksim Kholmatov:** Formal analysis. **Swati Garg:** Data curation; Formal analysis; Validation. **Cheng‐Chih Hsiao:** Data curation; Formal analysis. **Linda Viol:** Data curation; Formal analysis; Visualization; Writing – original draft. **Kaiqing Zhang:** Data curation; Formal analysis. **Rui Sun:** Formal analysis. **Christina Schmidt:** Formal analysis; Visualization. **Maike Janssen:** Software; Formal analysis; Writing – original draft. **Tara MacRae:** Data curation; Formal analysis. **Karin Huber:** Data curation; Writing – original draft. **Christian Thiede:** Resources. **Josée Hébert:** Resources. **Guy Sauvageau:** Resources; Writing – original draft. **Julia Spratte:** Resources. **Herbert Fluhr:** Resources; Supervision. **Gabriela Aust:** Resources; Writing – original draft. Carsten Müller‐Tidow: Resources; Writing – original draft. Christof Niehrs: Resources; Writing – original draft. **Gislene Pereira:** Resources; Supervision. **Jörg Hamann:** —Resources; Supervision. **Motomu Tanaka:** Supervision; Writing – original draft; Writing – review and editing. **Judith B Zaugg:** Supervision; Writing – original draft.

In addition to the CRediT author contributions listed above, the contributions in detail are:

LH performed experiments, analyzed data, generated figures, and wrote the manuscript. CA performed computational analyses, generated figures, and wrote the manuscript. LH and CA contributed equally to this work. JT performed dynamic phenotyping experiments and analyses, MK analyzed data and generated figures, SG contributed to *in vivo* experiments, C‐CH generated plasmids, LV performed IF imaging and provided reagents, KZ and RS performed Wnt reporter assays, CS and MJ performed synergism experiments and analyses, TM performed and analyzed *in vivo* experiments, CR performed computational analyses, KH helped with *in vivo* experiments and edited the manuscript, CT, JH, and GS provided primary AML samples and edited the manuscript, JS and HF generated CB units and edited the manuscript, GA supported SRF reporter assays, CM‐T edited the manuscript, CN provided reporter assays and supported Wnt experiments, GP provided expertise and reagents for primary cilium detection and analyses, JH provided plasmids and expertise on structure‐function analyses, MT supervised and analyzed dynamic phenotyping experiments and edited the manuscript, JBZ supervised all computational analyses, wrote the manuscript, and co‐supervised the project, CP directed the project, performed analyses, and wrote the manuscript.

## Disclosure and competing interests statement

The authors declare that they have no conflict of interest.

## Supporting information



Appendix

Dataset EV1

Dataset EV2

Dataset EV3

Dataset EV4

Dataset EV5

Dataset EV6

Dataset EV7

Dataset EV8

Dataset EV9

Dataset EV10

Dataset EV11

Dataset EV12

## Data Availability

Sequencing data generated for this study are accessible through GEO database (https://www.ncbi.nlm.nih.gov/geo/browse/) with the accession numbers: GSE150175 (http://www.ncbi.nlm.nih.gov/geo/query/acc.cgi?acc=GSE150175) for the GPR56 KD RNA‐seq data, GSE150868 (http://www.ncbi.nlm.nih.gov/geo/query/acc.cgi?acc=GSE150868) for the ATAC‐seq data. GPR56 expression following KD of TCF4 via shRNA in neuronal progenitors derived from iPSC was derived from Dataset EV4 of PMID31535015 (Doostparast Torshizi *et al*, 2019). For the integration with our human data, we created a mapping between the Ensembl IDs from human and mouse using biomaRt (Durinck *et al*, 2009). Public datasets used in this study were downloaded from GEO and comprise GSE147727 (http://www.ncbi.nlm.nih.gov/geo/query/acc.cgi?acc=GSE147727), GSE38236 (http://www.ncbi.nlm.nih.gov/geo/query/acc.cgi?acc=GSE38236), GSE70872 (http://www.ncbi.nlm.nih.gov/geo/query/acc.cgi?acc=GSE70872), and GSE48843 (http://www.ncbi.nlm.nih.gov/geo/query/acc.cgi?acc=GSE48843). We used the raw data whenever available (GSE111669; http://www.ncbi.nlm.nih.gov/geo/query/acc.cgi?acc=GSE111669) and processed them through DESeq2, in analogy to what has been described above. If no raw data were available or the data were also analyzed with DESeq2 (all other), we used the processed data (i.e., log_2_ fold‐changes and adjusted *P*‐values).
