# Supplementary material for: CDK7/12/13 inhibition targets an oscillating leukemia stem cell network and synergizes with venetoclax in acute myeloid leukemia
Source: EMBO Mol Med. 2022 Mar 7;14(4):e14990. doi: 10.15252/emmm.202114990 (PMC8988201; doi:10.15252/emmm.202114990)
Supplement: Supplementary file 1 — Appendix [file EMMM-14-e14990-s011.pdf]

CDK7/12/13 inhibition targets an oscillating leukemia stem cell network and synergizes with venetoclax in acute myeloid leukemia

Lixiazi He[1,2], Christian Arnold[2,3], Judith Thoma[4], Christian Rohde[1,2], Maksim Kholmatov[2,3], Swati Garg[1,2,5], Cheng-Chih Hsiao[6], Linda Viol[7,8], Kaiqing Zhang[9], Rui Sun[9], Christina Schmidt[1], Maike Janssen[1], Tara MacRae[10], Karin Huber[1], Christian Thiede[11], Josée Hébert[12,13,14], Guy Sauvageau[10,13,14], Julia Spratte[15], Herbert Fluhr[15], Gabriela Aust[16], Carsten Müller-Tidow[1,2], Christof Niehrs[9,17], Gislene Pereira[7,8], Jörg Hamann[6], Motomu Tanaka[4,18], Judith B. Zaugg[2,3], and Caroline Pabst[1,2+]

[1]Department of Medicine V, Hematology, Oncology and Rheumatology, University Hospital Heidelberg, Heidelberg, Germany;

[2]Molecular Medicine Partnership Unit (MMPU), University of Heidelberg and European Molecular Biology Laboratory (EMBL), Heidelberg, Germany;

[3]European Molecular Biology Laboratory (EMBL), Heidelberg, Germany;

[4]Physical Chemistry of Biosystems, Institute of Physical Chemistry, Heidelberg University, Heidelberg, Germany

[5]Department of Medical Oncology, Dana Farber Cancer Institute, Boston, MA, USA

[6]Department of Experimental Immunology, Amsterdam Infection & Immunity Institute, Amsterdam University Medical Centers, Amsterdam, Amsterdam, Netherlands;

[7]Centre for Organismal Studies (COS) / Centre for Cell and Molecular Biology (ZMBH), University of Heidelberg, Heidelberg, Germany;

[8]German Cancer Research Centre (DKFZ), DKFZ-ZMBH Alliance, Heidelberg, Germany;

[9]Division of Molecular Embryology, DKFZ-ZMBH Alliance, Heidelberg, Germany

[10]Laboratory of molecular genetics of stem cells, Institute for Research in Immunology and Cancer, University of Montreal, Montreal, Quebec, Canada;

[11]Department of Internal Medicine I, University Hospital of Dresden Carl Gustav Carus, Dresden, Germany

[12]The Quebec Leukemia Cell Bank and Division of Hematology-Oncology, Maisonneuve-Rosemont Hospital, Montréal, Canada;

[13]Department of Medicine, Faculty of Medicine, Université de Montréal, Montréal, Canada;

[14]Division of Hematology-Oncology, Maisonneuve-Rosemont Hospital, Montreal, Quebec, Canada;

[15]Department of Gynecology and Obstetrics, University Hospital Heidelberg, Heidelberg, Germany;

[16]Department of Surgery, Research Laboratories, Leipzig University, Leipzig, Germany;

[17]Institute of Molecular Biology (IMB), Mainz, Germany

[18]Center for Integrative Medicine and Physics, Institute for Advanced Study, Kyoto University, Kyoto, Japan

+Corresponding author: Caroline Pabst, University Hospital Heidelberg, Medical Department V, Hematology, Oncology, Rheumatology, Im Neuenheimer Feld 410, 69120 Heidelberg, Phone: +49-(0)6221-56-34121, Fax: +49-(0)6221-56-5419, Email: Caroline.Pabst@med.uni-heidelberg.de

Appendix

Table of content:
Appendix Figure S1-S6
Appendix Table S1-S3

Appendix Figure S1. GPR56 is associated with a Rho/Wnt/Hh network.
Appendix Figure S2. GPR56 is required for the maintenance of HSC and AML expansion.
Appendix Figure S3. GPR56 regulates Wnt and Hh pathways.
Appendix Figure S4. Reciprocal shift within GPR56+ LSC compartments is regulated by TGFb, Wnt and HH activities.
Appendix Figure S5. CDK7 inhibitors attenuate the GPR56 associated network.
Appendix Figure S6. CDK7 inhibition synergizes with venetoclax.

Appendix Table S1. List of shRNA oligos used in the study
Appendix Table S2. q-RT-PCR primers used in the study
Appendix Table S3. oligos used for generating overexpression constructs

Appendix Figure S1

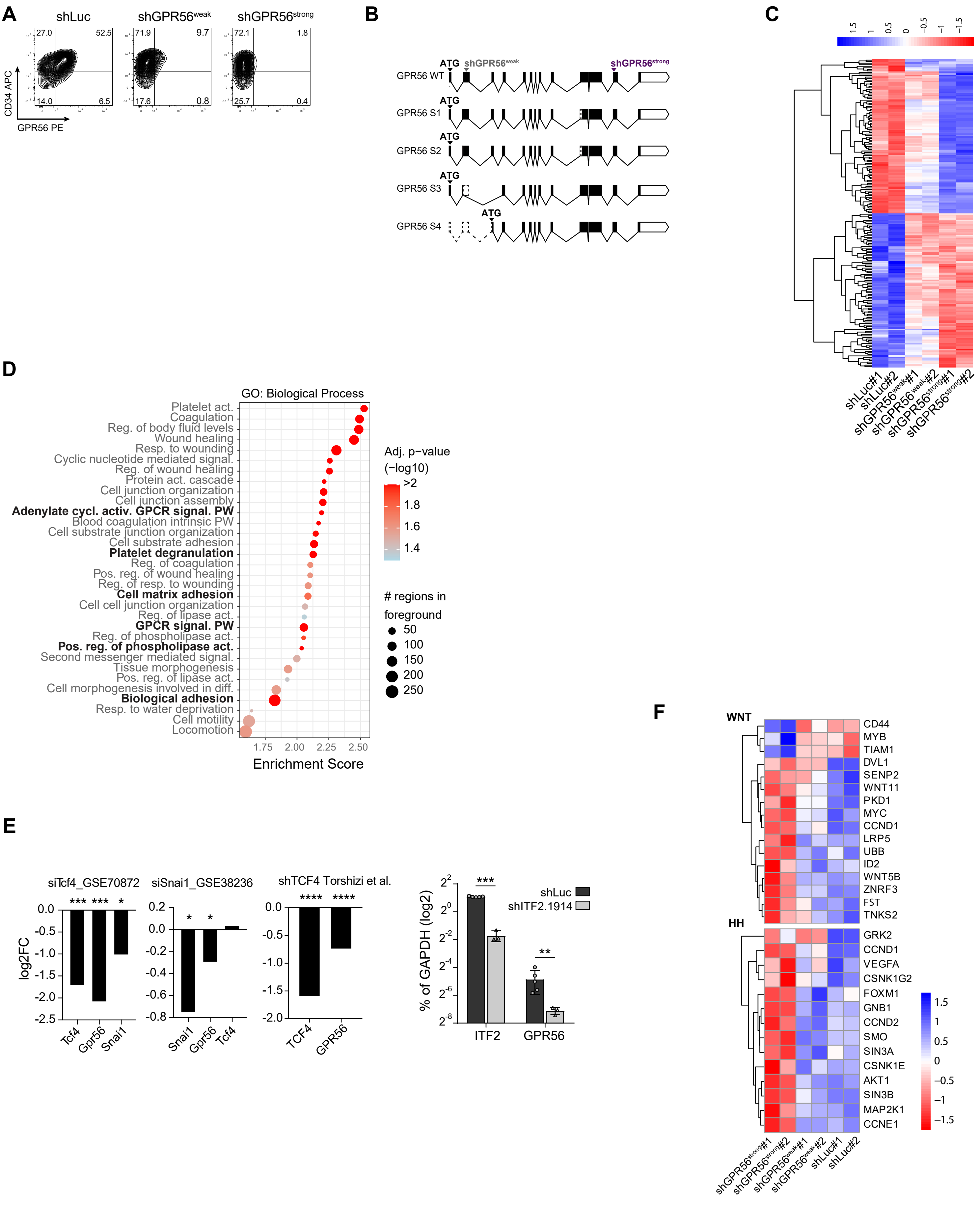

Appendix Fig S1. GPR56 is associated with a Rho/Wnt/Hh network.

A. FACS contour plots showing the loss of GPR56 protein expression in CD34 cells after GPR56 suppression via shRNAs.
B. Genomic scheme showing human GPR56 transcripts. The arrowheads indicate the translation start sites and positions of the two shRNAs, weak and strong, against GPR56. The black boxes represent the exons which are transcribed. The scheme was generated according to data from Uniprot and Ensembl database using wormweb software (http://wormweb.org/exonintron).
C. Heatmap of the top 200 genes (top 100 in either direction of log2FC of shGPR56^{strong} vs. shLuc) with an FDR < 0.1. Shown are the variance-stabilized values (using the *vst* function from the DESeq2 R package) that have been centered and scaled in row direction for visualization purposes.
D. GO term enrichment analysis of genes differentially expressed after GPR56 KD. Act.: activity, reg.: regulation, resp.: response, act.: activating, cycl: cyclase, signal.: signaling, PW: pathway, pos.: positive, diff.: differentiation.
E. Log2FC of Snai1, Tcf4, and Gpr56 gene expression after siRNA- mediated KD of Itf2/Tcf4 in mouse neuronal stem cells (GSE70872, *left*), of *Snail1* in mouse primary myoblasts (GSE38236, *middle*), and of *ITF2/TCF4* via shRNA in hiPSC-derived neural progenitor cells (Doostparast Torshizi *et al*, 2019). See **Dataset EV4** for values extracted from public datasets.
F. Heatmap showing log2-fold changes (shGPR56 vs. shLuc) of significantly differentially expressed genes (FDR<0.1) associated with Wnt (above) or Hh (below) pathways based on the literature (**Dataset EV6**).

Appendix Figure S2

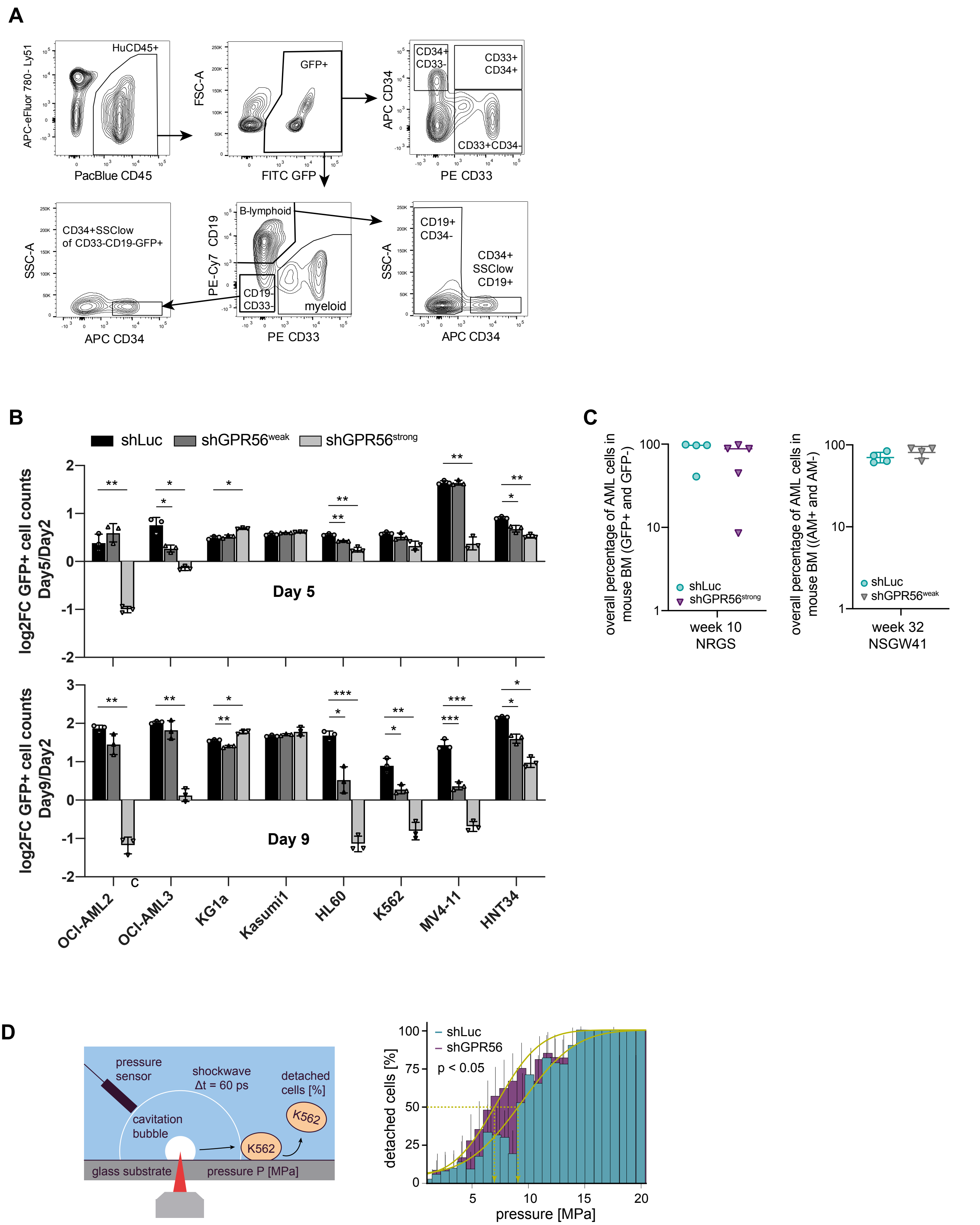

Appendix Fig S2. GPR56 is required for the maintenance of HSC and AML expansion.

A. Gating strategy for the detection of human cells in mouse bone marrow. Cells were gated on human CD45+ followed by gating on GFP+. Subsequently, the fraction of CD19+ B lymphatic and CD33+ myeloid cells and double negative cells were determined. CD33 and CD34 were used to distinguish myeloid committed progenitors (CD34+CD33+) and more mature myeloid cells (CD33+CD34-). CD34+ gating was also used to distinguish pre-B precursors (CD34+CD19+CD33-) and immature CD34+SSClowCD33-CD19- hematopoietic precursors comprising stem and progenitor cells.
B. KD of GPR56 in 8 leukemia cell lines with shGPR56^{weak} and shGPR56^{strong} versus shLuc control. Loss of the GFP+ fraction on day 5 (top) and day 9 (bottom) compared with day 2 indicates a proliferative disadvantage of GPR56 KD cells in the indicated cell lines. Three replicate wells were monitored by HTS-FACS per condition. Multiple t-tests, ns: not significant, * $p<0.05$, ** $p<0.005$, *** $p<0.0005$.
C. No difference in overall human leukemic engraftment in immunocompromised mice (including non-transduced cells) was observed excluding technical issues with injections.
D. Quantification of mechanical strength of cell adhesion. Left: Schematic illustration of quantification of cell adhesion strength using laser pulse induced pressure waves. Right: Fraction of detached cells plotted versus induced pressure for control (turquoise) and GPR56 KD cells (violet), distributions fitted with error functions (yellow) to determine the critical pressure for detachment. Reduction of adhesion strength by factor 1.2 in GPR56 KD cells ($p < 0.05$, Wald method for binomial distributions). N (control) = 2037, n (KD) = 2928.

Appendix Figure S3

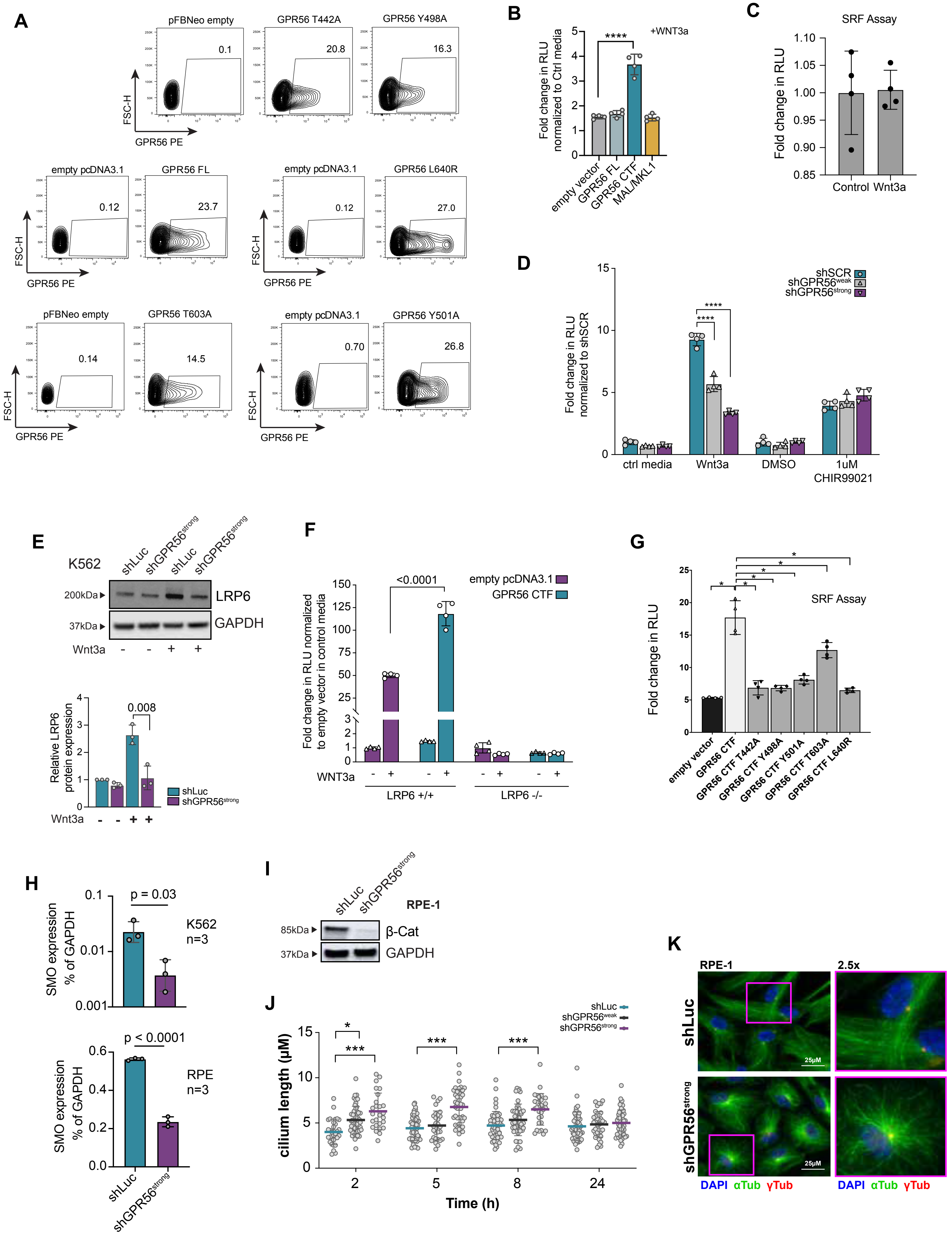

Appendix Fig S3. GPR56 regulates Wnt and Hh pathways.

A. FACS plots confirming GPR56 surface expression in HEK293T cells after transfection with GPR56 FL mutants in either pFBNeo or pcDNA3.1^{+} backbone. The same mutations were introduced in the GPR56-CTF version.
B. SuperTop reporter assay showing fold-change of RLU in presence of Wnt3a normalized to ctrl media after transfection of HEK293T cells with empty vector, GPR56 FL, GPR56 CTF, or the Rho/SRF activator MAL/MKL1 (Megakaryocytic Acute Leukemia).
C. SRF reporter assay indicating fold change in RLU after addition of Wnt3a conditioned media. Note that there is no difference in the signal caused by Wnt3a conditioned media. Means, stdev, four replicates are shown.
D. SuperTop reporter assay showing fold-change of RLU normalized to shSCR after addition of Wnt3a compared to control media (left two sets) or after addition of 1µM CHIR99021 compared to DMSO (right two sets). Note that shRNAs against GPR56 are unable to reduce the reporter signal induced by the GSK3 inhibitor CHIR99021.
E. β-catenin protein expression in K562 cells infected with shGPR56^{strong} or shLuc negative control in presence and absence of Wnt3a (representative Western Blot (above) and quantification after normalization to GAPDH (below). Shown is fold-change compared to shLuc in control media, bars and error bars represent mean and standard deviation of three biological replicates, unpaired t-test).
F. SuperTop reporter assay showing fold change in RLU in Wnt3a conditioned media (+) versus control media (-) after transfection with empty vector (violet) or GPR56 CTF (turquoise) in HEK293T wt or LRP6-/- KO cells normalized to the respective empty vector controls in control media. GPR56 CTF does not promote a SuperTop signal in the absence of LRP6. Unpaired t-test, bars and error bars represent mean and standard deviation of four biological replicates, **** $p<0.0001$.
G. SRF reporter assay indicating fold change in RLU after transfection of HEK293T cells with empty vector, GPR56-FL, or five GPR56-CTF mutants.
H. Relative *SMO* gene expression (percentage of *GAPDH*) in K562 (above) and RPE-1 (below) cells in shLuc versus shGPR56^{strong} cells determined by q-RT-PCR. Mean and stdev of three independent infections (biological replicates).
I. Western Blot showing β-catenin protein expression in RPE-1 cells after infection with shLuc or shGPR56^{strong} in two batches of RPE-1 cells.
J. Cilium length (mean, stdev, and individual values) measured in 26-50 cells per condition at indicated time points following the start of serum starvation.
K. Representative IF images showing DAPI, α- and γ-tubulin staining in RPE-1 cells infected with shLuc (above) or shGPR56^{strong} (below) in color mode (left, middle) or as black-and-white image (right, α-tubulin). Note the disturbed alignment of α-tubulin filaments upon GPR56 KD.

Appendix Figure S4

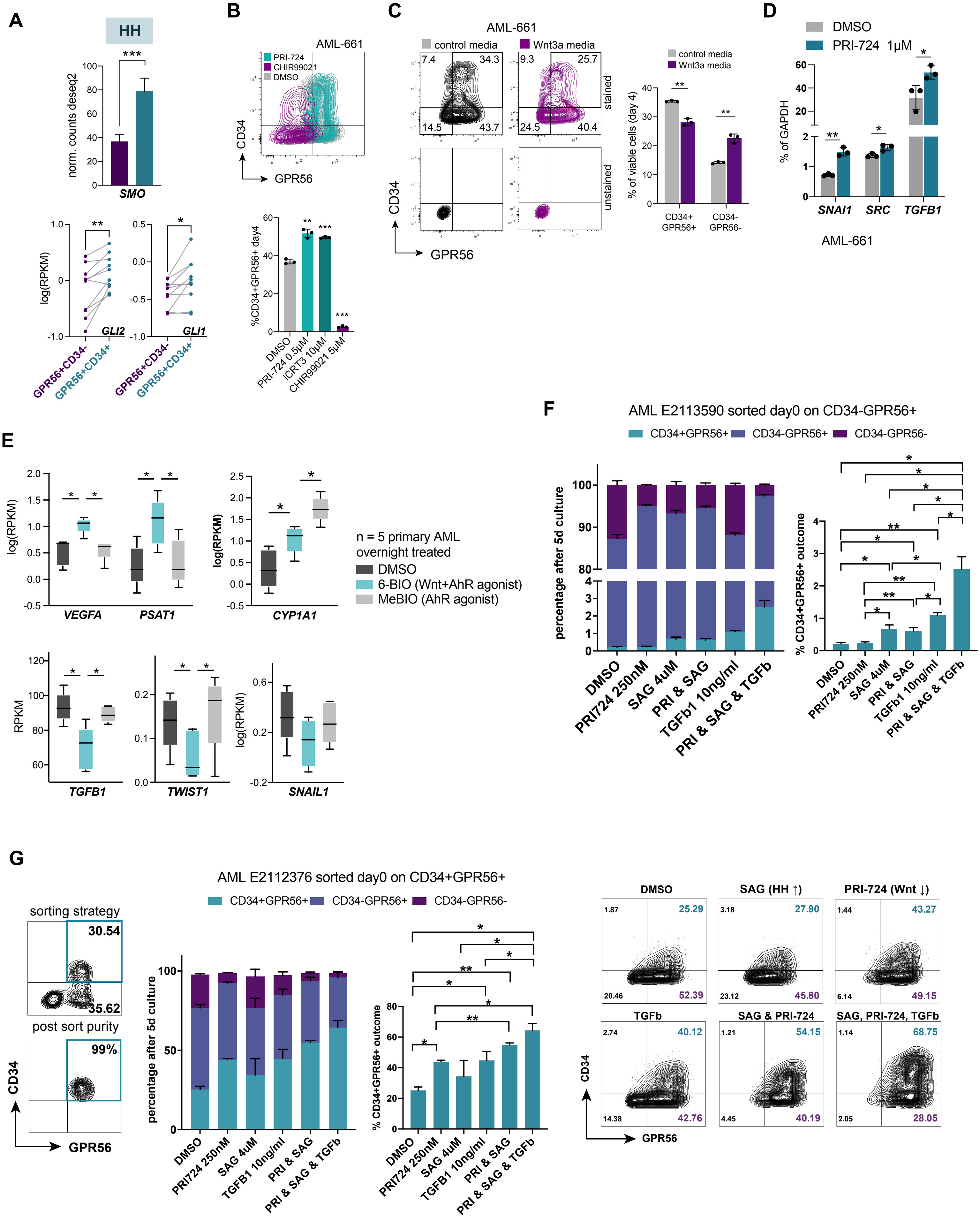

Appendix Fig S4. Reciprocal shift within GPR56+ LSC compartments is regulated by TGFb, Wnt and HH activities.

A. RNA-seq of $CD34^+$ cells showed suppressed *SMO* expression upon GPR56 knockdown. At the same time, expression of Hh targets *GLI1* and *GLI2* is higher in the $CD34^+GPR56^+$ versus the $CD34^-GPR56^+$ compartment suggesting higher Hh activity in the $CD34^+$ fraction. For D-F: * p<0.05, ** p<0.005, *** p<0.0005, non-adjusted p-values, see **Dataset EV2**.
B. *Left*: Contour plot showing changes of the $CD34^+GPR56^+$ fraction during treatment of PDX AML-661 with either the Wnt inhibitor PRI-724 (turquoise) or the Wnt agonist CHIR99021 (violet), respectively. *Right*: percentage of $CD34^+GPR56^+$ cells in AML-661 after 4-days treatment with PRI-724, iCRT3, CHIR99021 or DMSO. Summary of three replicates, unpaired t-test.
C. *Left*: FACS contour plots of AML-661 showing a decrease in the $CD34^+GPR56^+$ and an increase in the double negative fraction during treatment with Wnt3a conditioned media compared to control media. Unstained control plots show that there was no difference in baseline autofluorescence in the conditions. *Right*: summary of 3 replicate cultures, unpaired t-test.
D. Q-RT-PCR showing an increase in EMT gene expression (*SNAI1*, *SRC*, *TGFB1)* normalized to *GAPDH* after overnight treatment of PDX AML-661 with the Wnt inhibitor PRI-724. n=3 biological replicates.
E. *Top*: RNA-seq data revealing exclusive induction of Wnt targets *VEGFA* and *PSAT1* by the combined Wnt/AhR-agonist 6-BIO, while the more specific AhR-agonist MeBIO has no effect on expression of these two genes. Induction of AhR target gene *CYP1A1* in contrast is stronger with MeBIO compared to 6-BIO (GSE48843). *Bottom*: exclusive downregulation of *TGFB1*, *TWIST1*, and *SNAI1* by 6-BIO compared with MeBIO suggests that the effect was mostly due to the Wnt and not AhR activating activity of 6-BIO. Unpaired t-test, * p<0.05.
F. Bar graph showing the distribution of CD34 and GPR56 fractions at the end of the 5-day culture of purified $CD34^-GPR56^+$ cells from AML E2113590 with indicated compounds.
G. *Left*: FACS profile and sorting strategy for the $CD34^+GPR56^+$ fraction (top), and post-sort purity (bottom) of AML sample E2112376 on the day of thawing. *Middle*: Bar graph showing the distribution of CD34 and GPR56 fractions at the end of the 5-day culture of purified $CD34^-GPR56^+$ cells from AML E2112376 with indicated compounds. *Right*: representative FACS plots showing CD34 and GPR56 expression of purified $CD34^+GPR56^+$ cells exposed to the indicated molecules or their combinations for 5 days. Arrows indicate whether the pathway is inhibited (↓) or activated (↑) by the compound.

Appendix Figure S5

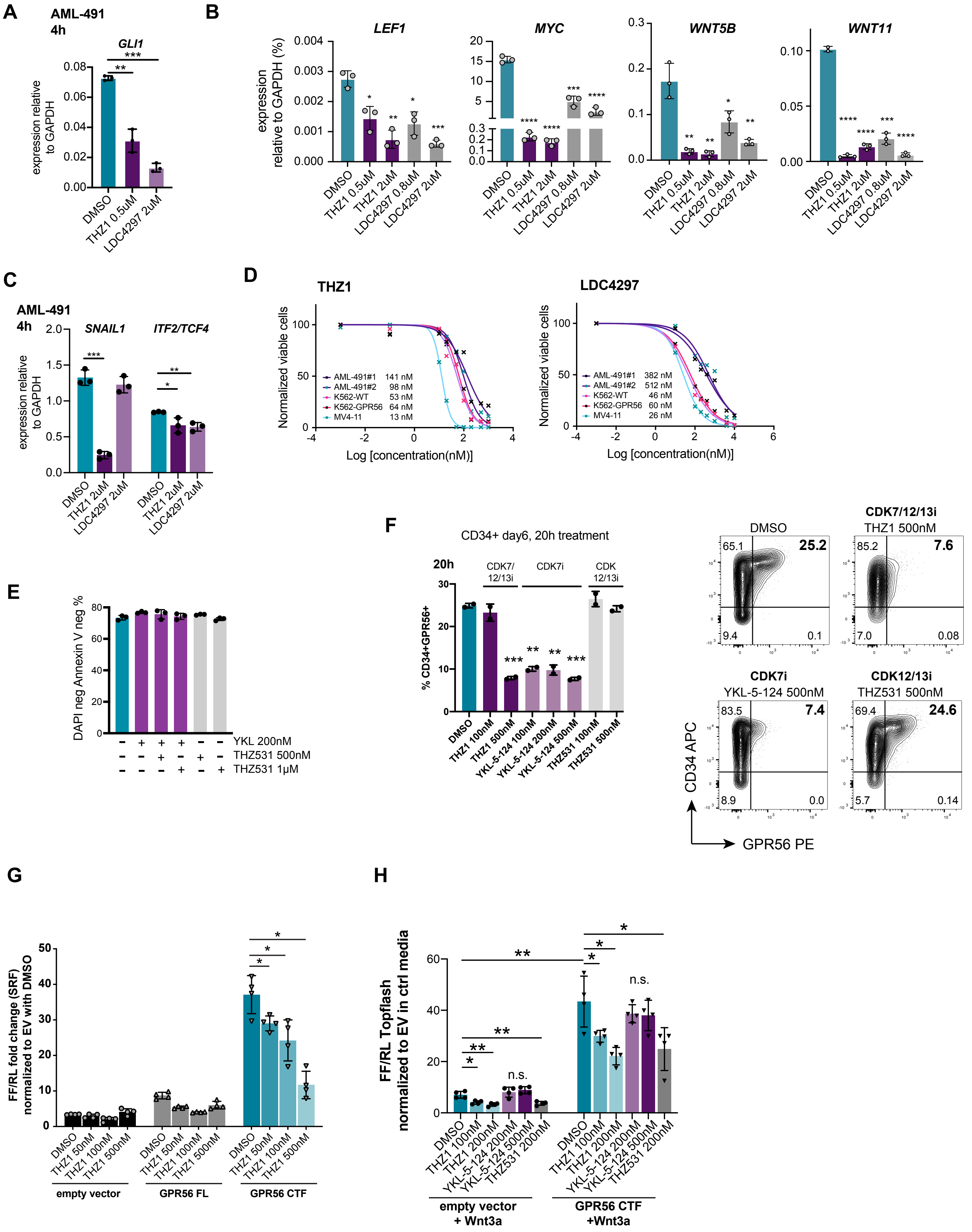

Appendix Fig S5. CDK7 inhibitors attenuate the GPR56 associated network.

A. Gene expression determined by q-RT-PCR for *GLI1* in PDX AML-491 after 4-hour treatment with THZ1 0.5µM or LDC4297 2µM. n=3 biological replicates.
B. Gene expression determined by q-RT-PCR for *LEF1*, *MYC*, *WNT5B*, and *WNT11* in PDX AML-491 after treatment with different doses of THZ1 or LDC4297. n=3 biological replicates.
C. *SNAIL1* and *ITF2* mRNA expression normalized to GAPDH in AML-491 cells determined by q-RT-PCR 4 hours after treatment with THZ1 0.5µM or LDC4297 2µM. Mean, stdev, and individual values from three to six culture wells are shown.
D. Dose curves for THZ1 (left) and LDC4297 (right) in two individual batches of the PDX AML-491 (#1 and #2), K562 WT, K562 cells overexpressing GPR56 (K562-GPR56), and MV4-11 cells. IC50 (nM) are indicated in the figure. Shown is one of two independent experiments. Three technical replicates per dose per experiment. x indicates means. Cell counts were normalized to the numbers of viable cells in vehicle DMSO.
E. Viability of HEL cells determined by DAPI and Annexin V staining after treatment with indicated drugs and doses. Note that the compounds had no major effect on viability.
F. Summary bar graph (left) and FACS plots (right) showing that only compounds with CDK7 inhibitory effect (THZ1 and YKL-5-124), but not the CDK12/13 inhibitor THZ531 reduce GPR56 on CB CD34+ cells.
G. SRF reporter assay revealing a dose-dependent suppression of the GPR56-CTF induced SRF signal by THZ1. Four technical replicates. One of four individual experiments.
H. SuperTop Wnt reporter assay showing that only THZ1 and THZ531, but not the CDK7 inhibitor YKL-5-124, which lacks CDK12/13 inhibitory activity, suppress the baseline (Wnt3a) and GPR56 CTF enhanced Wnt3 reporter signal. Four technical replicates. One of two individual experiments.

Appendix Figure S6

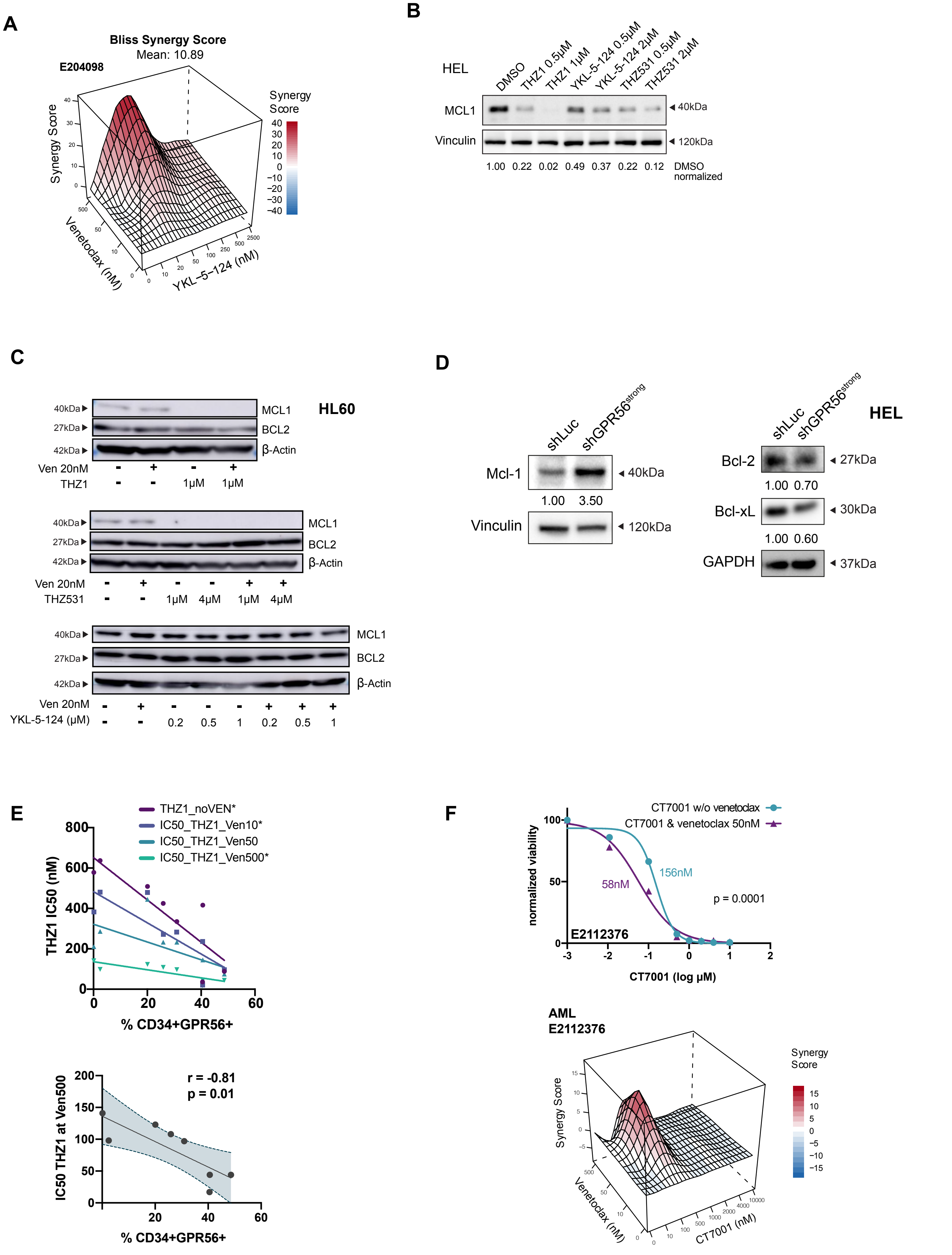

Appendix Fig S6. CDK7 inhibition synergizes with venetoclax.

A. 3D-map showing synergy scores (BLISS) between VEN and YKL-5-124 in the primary AML sample E204098.
B. Western Blot showing MCL-1 protein expression after overnight treatment with THZ1, YKL-5-124, or THZ531 in HEL cells at indicated doses. Vinculin protein expression was used as reference. One of two individual experiments.
C. Western Blot showing protein expression of Mcl-1, Bcl-2, and β-Actin in HL60 cells after treatment with VEN, THZ1, THZ531, or YKL-5-124 at indicated combinations and concentrations.
D. Western Blot showing protein expression of Mcl-1, Bcl-2, Bcl-xl in GPR56 depleted HEL cells. One of two individual experiments.
E. *Above:* Pearson correlation between IC50s for THZ1 and the percentage of CD34+GPR56+ cells in 8 primary human AML samples alone and with increasing concentrations of VEN. Asterisks indicate significant correlation. *Below:* Correlation plot between THZ1 IC50s and CD34+GPR56+ percentages in 8 primary human AML samples including standard deviation shown for THZ1 in presence of 500nM VEN (Ven500). Ven10: VEN at 10nM, Ven50: VEN at 50nM, Ven500: VEN at 500nM. Symbols represent in dividual samples.
F. *Above:* IC50 values in presence of increasing doses of VEN are visualized for CT7001 in AML primary sample E2112376. Unpaired t-test. *Below:* BLISS scores at each combination dose of CT7001 with VEN are shown for PDX AML sample E2112376.

Appendix Table S1. q-RT-PCR primers used in the study

**Oligonucleotides**	**5' --- 3'**
GAPDH forward	AGCCACATCGCTCAGACAC
GAPDH reverse	GCCCAATACGACCAAATCC
GPR56 forward	TATGTCCCTGGCTACCTACTC
GPR56 reverse	GTCCTATGCACAGCCAAGAT
SMO *forward*	TGCTCATCGTGGGAGGCTACTT
SMO reverse	ATCTTGCTGGCAGCCTTCTCAC
LRP6 forward	CAGTTGGAGTGGTGCTGAAAGG
LRP6 reverse	CCATCCAAAGCAGCCCGTTCAA
WNT11 forward	CTGTGAAGGACTCGGAACTCGT
WNT11 reverse	AGCTGTCGCTTCCGTTGGATGT
SNAI1 forward	TGCCCTCAAGATGCACATCCGA
SNAI1 reverse	GGGACAGGAGAAGGGCTTCTC
GLI1 forward	TGGAGGTCTGCGTGGTAGA
GLI1 reverse	TTGAACATGGCGTCTCAGG
CCNE1 forward	TGTGTCCTGGATGTTGACTGCC
CCNE1 reverse	CTCTATGTCGCACCACTGATACC
MYC forward	TGAAAGGCTCTCCTTGCAGC
MYC reverse	GCTGGTAGAAGTTCTCCTCC
LEF1 forward	CTACCCATCCTCACTGTCAGTC
LEF1 reverse	GGATGTTCCTGTTTGACCTGAGG
WNT5B forward	GTGCAGAGACCCGAGATGTT
WNT5B reverse	CAGGCTACGTCTGCCATCTT
ITF2/TCF4 forward	GCCTCTTCACAGTAGTGCCATG
ITF2/TCF4 reverse	GCTGGTTTGGAGGAAGGATAGC
TGFB1 forward	GCCTTTCCTGCTTCTCATGG
TGFB1 reverse	TCCTTGCGGAAGTCAATGTAC
SRC forward	CTGCTTTGGCGAGGTGTGGATG
SRC reverse	CCACAGCATACAACTGCACCAG

Appendix Table S2. List of shRNA oligos used in the study

Oligonucleotides	5' --- 3'
sh18 (shGPR56^{strong}) sense	CCGGCCTGGTGTTTCTGTTCAACATCTCGAGATGTTGAACAGAAACACCAGGTTTTTG
sh18 (shGPR56^{strong}) anti-sense	AATTCAAAAACCTGGTGTTTCTGTTCAACATCTCGAGATGTTGAACAGAAACACCAGG
sh19 (shGPR56^{weak}) sense	CCGGGACTTCTTGCTGAGTGACAAACTCGAGTTTGTCACTCAGCAAGAAGTCTTTTTG
sh19 (shGPR56^{weak}) anti-sense	AATTCAAAAAGACTTCTTGCTGAGTGACAAACTCGAGTTTGTCACTCAGCAAGAAGTC
shLuc sense	CCGGCGCTGAGTACTTCGAAATGTCCTCGAGGACATTTCGAAGTACTCAGCGTTTTTG
shLuc anti-sense	AATTCAAAAACGCTGAGTACTTCGAAATGTCCTCGAGGACATTTCGAAGTACTCAGCG
shScrambled sense	CCGGGCGCGATAGCGCTAATAATTTCTCGAGAAATTATTAGCGCTATCGCGCTTTTTG
shScrambled anti-sense	AATTCAAAAAGCGCGATAGCGCTAATAATTTCTCGAGAAATTATTAGCGCTATCGCGC

Appendix Table S3. oligos used for generating GPR56 overexpression constructs

Oligonucleotides	5' --- 3'
GPR56 CTF forward	ATGAATTCGCCACCATGACCTACTTTGCAGTGCTGATGGTCT
GPR56 CTF reverse	CTAAGCGGCCGCGATGCGGCTGGACGAGGTGCT
GPR56 CTF T442A forward	AAACCTCGGGACTACGCCATCAAGGTGCACATG
GPR56 CTF T442A reverse	CATGTGCACCTTGATGGCGTAGTCCCGAGGTTT
GPR56 CTF Y498A forward	TGGATGGGCCTCGAGGGGGCAAACCTCTACCGACTCGTGGTG
GPR56 CTF Y498A reverse	CACCACGAGTCGGTAGAGGTTTGCCCCCTCGAGGCCCATCCA
GPR56 CTF Y501A forward	CTCGAGGGGTACAACCTCGCACGACTCGTGGTGGAGGTC
GPR56 CTF Y501A reverse	GACCTCCACCACGAGTCGTGCGAGGTTGTACCCCTCGAG
GPR56 CTF T442A forward	AAACCTCGGGACTACGCCATCAAGGTGCACATG
GPR56 CTF T442A reverse	CATGTGCACCTTGATGGCGTAGTCCCGAGGTTT
GPR56 CTF R565W forward	ATGTGCTGGATCTGGGACTCCCTGGTC
GPR56 CTF R565W reverse	GACCAGGGAGTCCCAGATCCAGCACAT
GPR56 CTF L640R forward	CAGCTTGTCGTCCGCTACCTTTTCAGC
GPR56 CTF L640R reverse	GCTGAAAAGGTAGCGGACGACAAGCTG
GPR56 CTF T603A forward	CTGCGCCCCCACgCCCAAAAGTGGTCA
GPR56 CTF T603A reverse	TGACCACTTTTGGGcGTGGGGGCGCAG
