# Supplementary material for: CDK7/12/13 inhibition targets an oscillating leukemia stem cell network and synergizes with venetoclax in acute myeloid leukemia
Source: EMBO Mol Med. 2022 Mar 7;14(4):e14990. doi: 10.15252/emmm.202114990 (PMC8988201; doi:10.15252/emmm.202114990)
Supplement: Supplementary file 14 [file EMMM-14-e14990-s006.pdf]

EMBO Molecular Medicine

CDK7/12/13 inhibition targets an oscillating LSC network and synergizes with venetoclax in AML

Lixiazi He, Christian Arnold, Judith Thoma, Christian Rohde, Maksim Kholmatov, Swati Garg, Cheng-Chih Hsiao, Linda Viol, Kaiqing Zhang, Rui Sun, Christina Schmidt, Maike Janssen, Tara MacRae, Karin Huber, Christian Thiede, Josee Hebert, Guy Sauvageau, Julia Spratte, Herbert Fluhr, Gabriela Aust, Carsten Müller-Tidow, Christof Niehrs, Gislene Pereira, Jörg Hamann, Motomu Tanaka, Judith Zaugg, Caroline Pabst
DOI: 10.15252/emmm.202114990

Corresponding author(s): Caroline Pabst (pabst0404@gmail.com)

Review Timeline:

Submission Date:	14th Aug 21
Editorial Decision:	22nd Sep 21
Revision Received:	13th Jan 22
Editorial Decision:	26th Jan 22
Revision Received:	6th Feb 22
Accepted:	7th Feb 22

Editor: Jingyi Hou

Transaction Report:

(Note: With the exception of the correction of typographical or spelling errors that could be a source of ambiguity, letters and reports are not edited. Depending on transfer agreements, referee reports obtained elsewhere may or may not be included in this compilation. Referee reports are anonymous unless the Referee chooses to sign their reports.)

22nd Sep 2021

Dear Dr. Pabst,

Thank you for submitting your work to EMBO Molecular Medicine. We have now heard back from two of the three referees who agreed to evaluate your manuscript. Unfortunately, after a series of reminders, we did not obtain a report from Referee #3. In the interest of time, we have decided to proceed with these two reports. As you will see below, the referees acknowledge the potential interest and relevance of the study. However, they also raise a series of concerns about your work, which should be convincingly addressed in a major revision of the present manuscript.

I think that the referees' recommendations are rather clear, so there is no need to reiterate the points listed below. Importantly, Referee #2's main concern (point #3) should be carefully addressed. All other issues raised by the referees need to be satisfactorily addressed as well.

We would welcome the submission of a revised version within three months for further consideration. Please note that EMBO Molecular Medicine strongly supports a single round of revision. As acceptance or rejection of the manuscript will depend on another round of review, your responses should be as complete as possible.

EMBO Molecular Medicine has a "scooping protection" policy, whereby similar findings that are published by others during review or revision are not a criterion for rejection. Should you decide to submit a revised version, I do ask that you get in touch after three months if you have not completed it to update us on the status.

We are aware that many laboratories cannot function at full efficiency during the current COVID-19/SARS-CoV-2 pandemic and have therefore extended our "scooping protection policy" to cover the period required for a full revision to address the experimental issues. Please let me know should you need additional time, and also if you see a paper with related content published elsewhere.

Please read below for important editorial formatting and consult our author's guidelines for proper formatting of your revised article for EMBO Molecular Medicine.

I look forward to receiving your revised manuscript.

Use this link to login to the manuscript system and submit your revision: https://embomolmed.msubmit.net/cgi-bin/main.plex

Kind regards,
Jingyi

Jingyi Hou
Editor
EMBO Molecular Medicine

*****

When submitting your revised manuscript, please carefully review the instructions that follow below. We perform an initial quality control of all revised manuscripts before re-review; failure to include requested items will delay the evaluation of your revision.

We require:

1) A .docx formatted version of the manuscript text (including legends for main figures, EV figures and tables). Please make sure that the changes are highlighted to be clearly visible.

2) Individual production quality figure files as .eps, .tif, .jpg (one file per figure). For guidance, download the 'Figure Guide PDF' (https://www.embopress.org/page/journal/17574684/authorguide#figureformat).

3) A .docx formatted letter INCLUDING the reviewers' reports and your detailed point-by-point responses to their comments. As part of the EMBO Press transparent editorial process, the point-by-point response is part of the Review Process File (RPF), which will be published alongside your paper.

4) A complete author checklist, which you can download from our author guidelines (https://www.embopress.org/page/journal/17574684/authorguide#submissionofrevisions). Please insert information in the checklist that is also reflected in the manuscript. The completed author checklist will also be part of the RPF.

5) Please note that all corresponding authors are required to supply an ORCID ID for their name upon submission of a revised manuscript.

6) It is mandatory to include a 'Data Availability' section after the Materials and Methods. Before submitting your revision, primary datasets produced in this study need to be deposited in an appropriate public database, and the accession numbers and database listed under 'Data Availability'. Please remember to provide a reviewer password if the datasets are not yet public (see https://www.embopress.org/page/journal/17574684/authorguide#dataavailability).

In case you have no data that requires deposition in a public database, please state so in this section. Note that the Data Availability Section is restricted to new primary data that are part of this study.

7) For data quantification: please specify the name of the statistical test used to generate error bars and P values, the number (n) of independent experiments (specify technical or biological replicates) underlying each data point and the test used to calculate p-values in each figure legend. The figure legends should contain a basic description of n, P and the test applied. Graphs must include a description of the bars and the error bars (s.d., s.e.m.).

8) We would also encourage you to include the source data for figure panels that show essential data. Numerical data should be provided as individual .xls or .csv files (including a tab describing the data). For blots or microscopy, uncropped images should be submitted (using a zip archive if multiple images need to be supplied for one panel). Additional information on source data and instruction on how to label the files are available at

.

9) Our journal encourages inclusion of *data citations in the reference list* to directly cite datasets that were re-used and obtained from public databases. Data citations in the article text are distinct from normal bibliographical citations and should directly link to the database records from which the data can be accessed. In the main text, data citations are formatted as follows: "Data ref: Smith et al, 2001" or "Data ref: NCBI Sequence Read Archive PRJNA342805, 2017". In the Reference list, data citations must be labeled with "[DATASET]". A data reference must provide the database name, accession number/identifiers and a resolvable link to the landing page from which the data can be accessed at the end of the reference. Further instructions are available at .

10) We replaced Supplementary Information with Expanded View (EV) Figures and Tables that are collapsible/expandable online. A maximum of 5 EV Figures can be typeset. EV Figures should be cited as 'Figure EV1, Figure EV2" etc... in the text and their respective legends should be included in the main text after the legends of regular figures.

- For the figures that you do NOT wish to display as Expanded View figures, they should be bundled together with their legends in a single PDF file called *Appendix*, which should start with a short Table of Content. Appendix figures should be referred to in the main text as: "Appendix Figure S1, Appendix Figure S2" etc.

- Additional Tables/Datasets should be labeled and referred to as Table EV1, Dataset EV1, etc. Legends have to be provided in a separate tab in case of .xls files. Alternatively, the legend can be supplied as a separate text file (README) and zipped together with the Table/Dataset file.

See detailed instructions here:

.

11) The paper explained: EMBO Molecular Medicine articles are accompanied by a summary of the articles to emphasize the major findings in the paper and their medical implications for the non-specialist reader. Please provide a draft summary of your article highlighting

- the medical issue you are addressing,

- the results obtained and

- their clinical impact.

This may be edited to ensure that readers understand the significance and context of the research. Please refer to any of our

published articles for an example.

12) For more information: There is space at the end of each article to list relevant web links for further consultation by our readers. Could you identify some relevant ones and provide such information as well? Some examples are patient associations, relevant databases, OMIM/proteins/genes links, author's websites, etc...

13) Author contributions: the contribution of every author must be detailed in a separate section (before the acknowledgments).

14) A Conflict of Interest statement should be provided in the main text.

15) Every published paper now includes a 'Synopsis' to further enhance discoverability. Synopses are displayed on the journal webpage and are freely accessible to all readers. They include a short stand first (maximum of 300 characters, including space) as well as 2-5 one-sentences bullet points that summarizes the paper. Please write the bullet points to summarize the key NEW findings. They should be designed to be complementary to the abstract - i.e. not repeat the same text. We encourage inclusion of key acronyms and quantitative information (maximum of 30 words / bullet point). Please use the passive voice. Please attach these in a separate file or send them by email, we will incorporate them accordingly.

Please also suggest a striking image or visual abstract to illustrate your article as a PNG file 550 px wide x 300-600 px high.

EMBO Molecular Medicine has a "scooping protection" policy, whereby similar findings that are published by others during review or revision are not a criterion for rejection. Should you decide to submit a revised version, I do ask that you get in touch after three months if you have not completed it, to update us on the status.

***** Reviewer's comments *****

Referee #1 (Comments on Novelty/Model System for Author):

The experimental models are good and the experiments are well controlled

Referee #1 (Remarks for Author):

This manuscript "CDK7/12/13 inhibition targets an oscillating LSC network and synergizes with venetoclax in AML" describes how GPR56 expressing cells comprise leukemia stem cell (LSC)-enriched compartment, and how GPR56 co-activates Wnt and Hh/EMT signaling to maintain LSCs. In addition, the manuscript proposes combination therapy with CDK7/12/13 inhibition and venetoclax to target this cell population. Since the existence of LSCs is one of the reasons for chemotherapy resistance and disease relapse and LSC-targeting therapy is an urgent necessity, this problem is of high importance. Additional experiments are advised to prove the mechanism of regulation of the two LSC compartments, and to clarify the importance of GRP56 signaling in the proposed therapeutic strategy.

Major comments:

1. In Figure 1e-f and Supplemental Figure 1e (p.6-7), ATAC-seq data from GRP56hi vs GRP56low AML patient samples and RNA-seq data from shGRP56 KD CD34+ cord blood cells are aligned together. This is somewhat confusing. It would be better to show RNA-seq data from the same AML patient cohort, and separately from shRNA modified cells.
2. The mechanism of the transition between two LSC compartments, CD34+GRP56+ slow LSCs and CD34-GRP56 fast LSCs, is shown in Figure 4i. It would be more convincing to show the regulation of these two populations in sorted cells upon experimental modulation of Wnt and Hh/EMT signaling.
3. In Figure 5 and 6, THZ1, CDK7/12/13 inhibitor, is used for targeting GRP56+ LSC compartments. By virtue of inhibiting Mediator functions across many genes, CDK7 inhibition can have pleiotropic effects on gene expression. If GRP56 is required for the anti-leukemia efficacy of THZ1, then enforced Mediator-independent expression of GRP56 should confer resistance. Mutants of GRP56 can serve as positive and negative controls.
4. It would be also informative to examine whether any differences exist in the sensitivity to THZ1 with or without venetoclax treatment between GRP56 high and GRP56 low AML cells.
5. What is the anti-leukemia efficacy of the combination therapy as opposed to the monotherapy with THZ1 in vivo? Can this combination completely delete GRP56+ populations?

Minor comments:

- AML affects both young and elderly people... (line 60)
- In Figure 1B, does {greater than or equal to}15 mean 15 {less than or equal to} AML < 30? and dose {greater than or equal to}1

mean 1 {less than or equal to} AML < 15?
- In Figure 2b-d, it seems that shGRP56 transduced CB CD34+ cells (GFP+ cells) were not sorted before transplantation. In that case, it may be informative to show the cell populations separately, GFP+ and GFP- cells, among human CD45 cells.
- In Figure 2a and Supplements Figure 2b, delta GFP% could be affected by the baseline transduction efficiency. It might be better to show fold changes?
- In Supplemental Figure 2c, do these graphs mean that most engrafted leukemia cells are GFP negative in both shLuc and shGRP56 condition?
- The reduced adhesion and deformation capacity in GRP56 KD K562 cells are shown in Figure 2d-e (p.8). Is there any correlation to LSC properties?
- In Figure 3: some of the experiments were done by using HEK293T or RPE cells, not AML cells...Reduced ciliation in sgGRP56 RPE-1 cells is shown, through SMO suppression. What is the effect of SMO regulation by GRP56 in AML cells?
- In Figure 6, reduced MCL-1 expression is nominated as an advantage in the combination therapy. Is MCL-1 regulated by GRP56? Or is it an independent factor to the resistance to the therapy?

Referee #2 (Comments on Novelty/Model System for Author):

State of the art molecular techniques

Referee #2 (Remarks for Author):

This manuscript hypothesizes that AML (stem cells) may be able to escape therapy by oscillating between two states, a GRP56highCD34+ and a GRP56lowCD34-.

GRP56 interacts in the different cell states with different signalling pathways at receptor level, specifically with WNT signalling via lpr6 co-receptor and Hedgehog signalling via SMO. The authors claim that activation of WNT signalling shifts the balance between the two cell states towards the GPP56highCD34low phenotype.

The conclusion of this paper is that GRPhigh leukemic stem cells need to be targeted upstream of GRP signalling by targeting Rho (via inhibition of CDK7) and the Wnt pathway (by inhibition of CDK12/13). As CDK12/13 inhibition also suppresses MCL-1 there is synergy between CDK12/13 and BCL2 inhibition (Venetoclax).

I am intrigued by this model but I think there are still some open ends that need to be addressed.

Key points:

> I found the manuscript very difficult to read and needed to make my own drawings to understand the interaction between the different pathway. There are also way too many abbreviations. More data should go in the supplements, also to make the figures less loaded with data.

> An overall figure (and not just tiny subfigures) explaining the proposed interaction of GPR56 with the other pathways and where the many inhibitors hit these pathways as well as a figure explaining the model of the oscillating stem cell states would be essential to make this work comprehensible for the nonspecialist.

> My main concern is that the effect of inhibiting/activating Wnt signalling on the GRP56highCD34+ population is only shown in own patient-derived sample (AML661) and although one sees a loss/increase of this population I struggled to see that there is a shift from GRP56highCD34+ to GRP56highCD34- cells of vice versa. Most of the data are inferential rather than providing an actual proof for the oscillation between the different stem cell states.

Referee #1 (Comments on Novelty/Model System for Author):

The experimental models are good and the experiments are well controlled

Referee #1 (Remarks for Author):

This manuscript "CDK7/12/13 inhibition targets an oscillating LSC network and synergizes with venetoclax in AML" describes how GPR56 expressing cells comprise leukemia stem cell (LSC)-enriched compartment, and how GPR56 co-activates Wnt and Hh/EMT signaling to maintain LSCs. In addition, the manuscript proposes combination therapy with CDK7/12/13 inhibition and venetoclax to target this cell population. Since the existence of LSCs is one of the reasons for chemotherapy resistance and disease relapse and LSC-targeting therapy is an urgent necessity, this problem is of high importance. Additional experiments are advised to prove the mechanism of regulation of the two LSC compartments, and to clarify the importance of GRP56 signaling in the proposed therapeutic strategy.

Major comments:

1. In Figure 1e-f and Supplemental Figure 1e (p.6-7), ATAC-seq data from GRP56hi vs GRP56low AML patient samples and RNA-seq data from shGRP56 KD CD34+ cord blood cells are aligned together. This is somewhat confusing. It would be better to show RNA-seq data from the same AML patient cohort, and separately from shRNA modified cells.

We thank referee#1 for this valuable comment, which considerably improved Figure 1. RNA-seq data from 9 GPR56^{high} and 11 GPR56^{low} AML samples were available from the Montreal

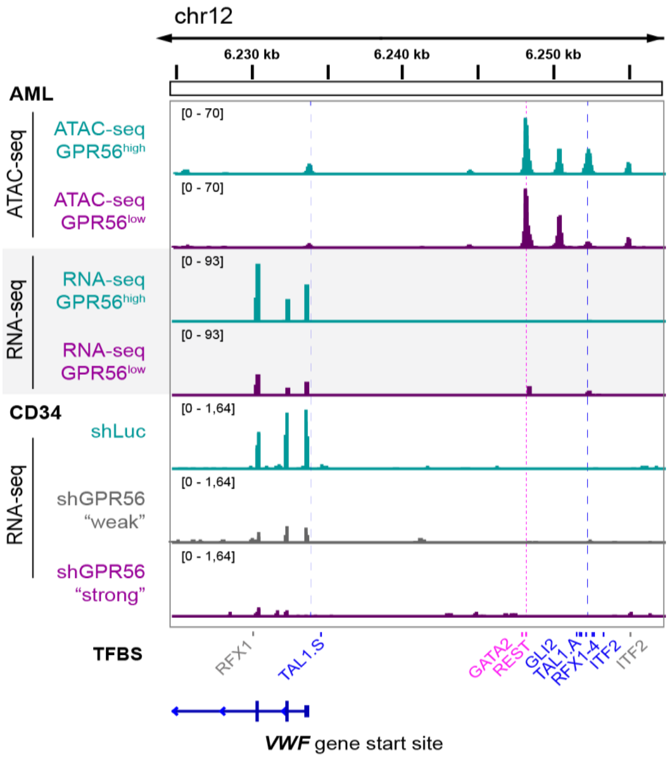

Fig. R1. IGV plot visualizing differential chromatin accessibility upstream of the *VWF* gene (ATAC-seq peaks upper track), higher RNA expression in AML with high vs low GPR56 expression (middle track), and reduced RNA expression in CD34+ cells upon GPR56 knockdown (KD, level dependent). While the GATA2 and REST binding sites are located at a similarly accessible region (pink), the differential peaks contain binding sites for TAL1, RFX1-4, and ITF2/TCF4, which were also significantly enriched in GPR56high AML in the global genome-wide *diffTF* analysis (see **rev_Fig 1F**).

cohort (**indicated in revised Dataset EV1**). We were thus able to add a track for averaged RNA-seq reads below the ATAC-seq data in the IGV plot in **rev_Fig 1E** and **Fig. R1** below. We brought the *VWF* gene from original Suppl. Fig. 1 to the main Figure and removed the example for *CD9*, as the differential ATAC-seq peaks upstream of *VWF* contained binding motifs of many TFs, which were also significantly enriched in our global genome-wide *diffTF* analysis (**rev_Fig 1F**.). Moreover, differences in RNA expression in the two AML groups was stronger for *VWF*. We kept the IGV track from CD34 RNA-seq data below the AML data to illustrate how *VWF* gene expression changes upon GPR56 knockdown.
We moved some FACS plots from **rev_Fig 1D** to **Appendix Fig S1A** and removed the two subfigures Fig. S1D and S1F of the original Supplemental Fig. 1 including corresponding text passages to reduce the figure load and simplify the text, as suggested by referee#2.

2. The mechanism of the transition between two LSC compartments, CD34+GRP56+ slow LSCs and CD34-GRP56 fast LSCs, is shown in Figure 4i. It would be more convincing to show the regulation of these two populations in sorted cells upon experimental modulation of Wnt and Hh/EMT signaling.

We are grateful for this comment, as the suggested experiments provided substantial additional information that also addressed the most critical point raised by referee#2 (see referee#2 point 3). We sorted CD34+GPR56+ and CD34-GPR56+ fractions from two different primary AML samples. The sorted fractions were treated independently with either the Wnt agonist CHIR99021, or combinations of the Wnt inhibitor PRI-724, the Hedgehog-agonist SAG, and TGFbeta as the key protein associated with epithelial-to-mesenchymal transition (EMT) in solid cancers. With this setup we were able to demonstrate
a) enhanced differentiation of purified CD34+GPR56+ to CD34-GPR56+ cells with the Wnt agonist CHIR99021 compared control conditions (**Fig. R2, rev_Fig 4C**)
b) *in vitro* generation of CD34+GPR56+ from CD34-GPR56+ sorted cells
To induce de-differentiation of CD34-GPR56+ to CD34+GPR56+ cells, we used the Wnt

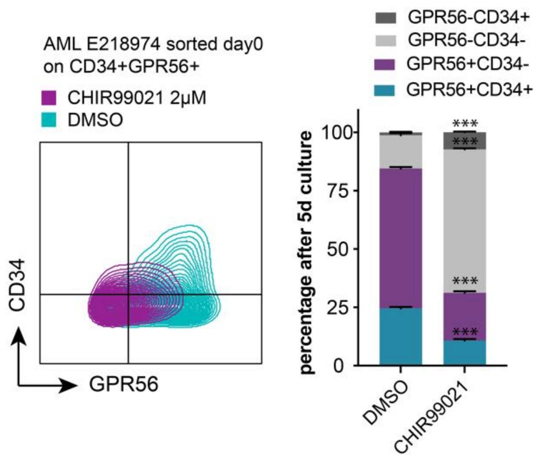

Fig. R2. Contour FACS plots (left) and summary bar graph (right) showing CD34 and GPR56 expression after 5-day culture of purified CD34+GPR56+ cells from AML sample E218974 with CHIR99021 or vehicle DMSO, n=3.

inhibitor PRI-724, the Hh agonist SAG, and recombinant TGFb alone and in combination and added these to CD34-GPR56+ cells purified from two different primary human AML samples (**Fig. R3, rev_Fig 4D-E, Appendix Fig S4F**) Thus, we were able to show that transition from CD34-GPR56+ to CD34+GPR56+ was most efficient with the combination of all three molecules. This was specifically striking, as there is a natural tendency of CD34+GPR56+ to

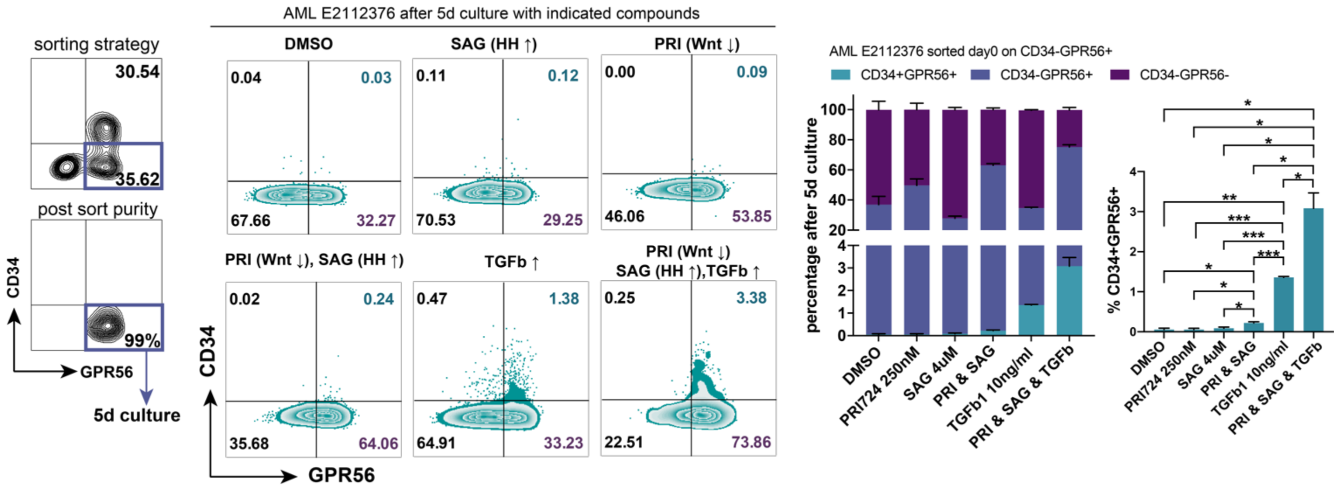

Fig. R3. *Left:* FACS contour plots of AML E2112376 before (top) and after (bottom) sorting of CD34-GPR56+ cells. *Middle:* FACS plots after 5-day treatment of the purified CD34-GPR56+ cells with the indicated compounds or combinations. *Right*: Quantification of percentages of CD34 and GPR56 expressing cells after 5-day treatment with indicated compounds and combinations. N = 3, pairwise t-tests, BH-adjusted p-values.

differentiate to CD34-GPR56+ cells *in vitro* (Pabst *et al*, 2014).

There was a clear synergism between PRI-724, SAG, and TGFb to generate CD34+GPR56+ from CD34-GPR56+ cells, as the single substances had only marginal effects on their own.

c) delayed differentiation of CD34+GPR56+ to CD34-GPR56+ sorted cells

Treatment of the CD34+GPR56+ sorted cells with the three substances significantly prevented the spontaneous differentiation towards the CD34-GPR56- status that occurs in standard culture conditions (**Fig. R4, Appendix Fig S4G**). Note that post-sort purity was 99% for the two fractions at the start of the culture.

Together, these sorting experiments corroborated the suggested mechanism that Wnt inhibition together with Hh/TGFb activation enables regeneration of the CD34+GPR56+ compartment from the CD34- fraction, while Wnt agonism promotes the shift from the CD34+GPR56+ towards the CD34-GPR56- status. Furthermore, these experiments showed that our suggested mechanism is also observed in primary AML samples from different patients, which have never been passaged in mice. We added these novel data to **rev_Fig 4D-E and Appendix Fig S4F-G** and added text to methods and results accordingly.

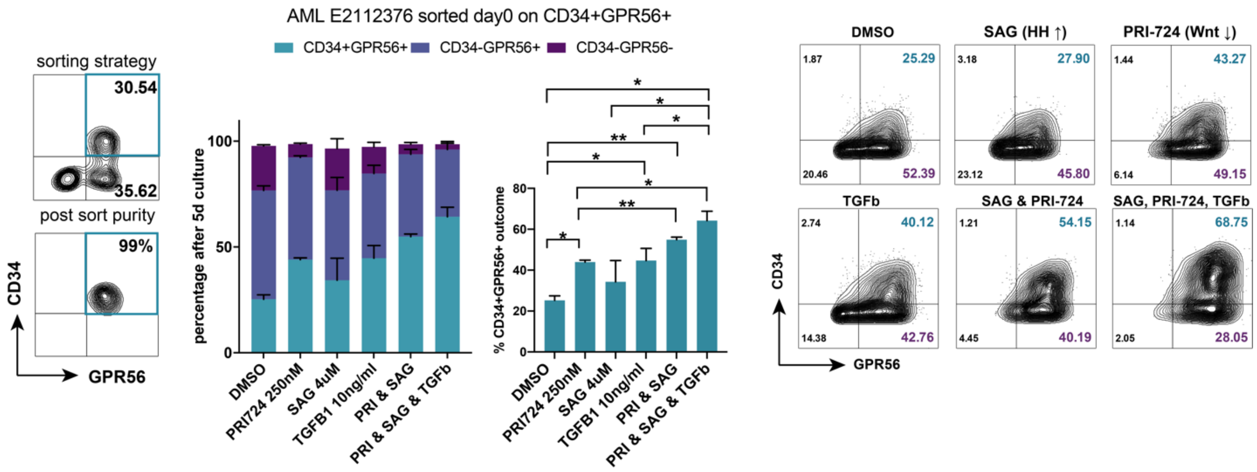

Fig. R4. *Left*: FACS contour plots of AML E2112376 showing the sorting strategy for in vitro treatment before (top) and after (bottom) sorting of CD34+GPR56+ cells. *Middle*: Quantification of percentages of CD34 and GPR56 expressing cells after 5-day treatment with SAG, PRI-724, and TGFb alone or with the indicated combinations. Statistical analysis is visualized only for CD34+GPR56+ output cells. For complete statistical group comparisons see dataset EV11. *Right*: representative FACS plots showing CD34 and GPR56 expression after 5-day treatment with the indicated compounds or combinations. N = 3, pairwise t-tests, BH-adjusted p-values.

3. In Figure 5 and 6, THZ1, CDK7/12/13 inhibitor, is used for targeting GRP56+ LSC compartments. By virtue of inhibiting Mediator functions across many genes, CDK7 inhibition can have pleiotropic effects on gene expression. If GRP56 is required for the anti-leukemia efficacy of THZ1, then enforced Mediator-independent expression of GRP56 should confer resistance. Mutants of GRP56 can serve as positive and negative controls.

We fully agree with the referee that THZ1 has pleiotropic effects even beyond CDK7 inhibition (e.g. CDK12/13 inhibition, suppression of *MYC* and *MCL1*). Specifically for MCL1, we showed before that the more specific CDK7i (YKL-5-124) caused little to no MCL1 suppression in HEL and HL60 cells, respectively (**Appendix Fig S6B-C**). We added novel data showing that GPR56 knockdown also does not suppress MCL1 protein levels or rather increases them (**Fig. R5, Appendix Fig S6D**). As MCL1 suppression by THZ1 will always have effects on cell viability, it cannot be expected that GPR56 overexpression alone rescues the anti-leukemic activity of THZ1 treatment. Nonetheless, we overexpressed GPR56 by lentiviral transduction in OCI-AML2 and OCI-AML3 (both GPR56 negative cell lines), and we did not see differences in IC50 for THZ1, which might also be due to the fact

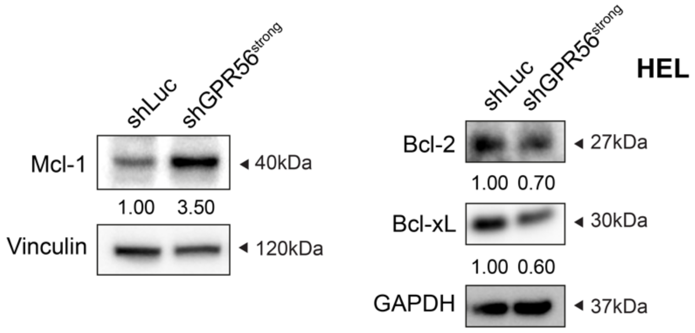

Fig. R5. Western Blot showing protein expression for Mcl-1, Vinculin, Bcl-2, Bcl-xL and GAPDh in HEL cells after GPR56 KD versus shLuc control.

that these are immortalized cell lines growing independently of GPR56. We were not able to achieve high protein overexpression with lentivirus in GPR56 negative primary AML samples. This might be due to the fact that GPR56 negative AML samples often do not proliferate or do not even survive *in vitro*. We added the Western Blot and commented on it in **Appendix Fig S6D**.

4. It would be also informative to examine whether any differences exist in the sensitivity to THZ1 with or without venetoclax treatment between GRP56 high and GRP56 low AML cells.

We thank again the referee for this valuable suggestion. We determined CD34/GPR56 FACS profiles for 8 primary AML samples with normal karyotype for better homogeneity. We then correlated the percentage of CD34+GPR56+ surface expression with the IC50s for THZ1 in presence and absence of venetoclax. We found that samples responded better to THZ1, the greater the CD34+GPR56+ fraction. This observation indicates that AML samples with high CD34+GPR56+ fraction are more dependent on pathways affected THZ1. Addition of venetoclax flattened the regression curve, but the correlation was still significant further supporting that the compounds do not hit the same pathways. These results are also in line

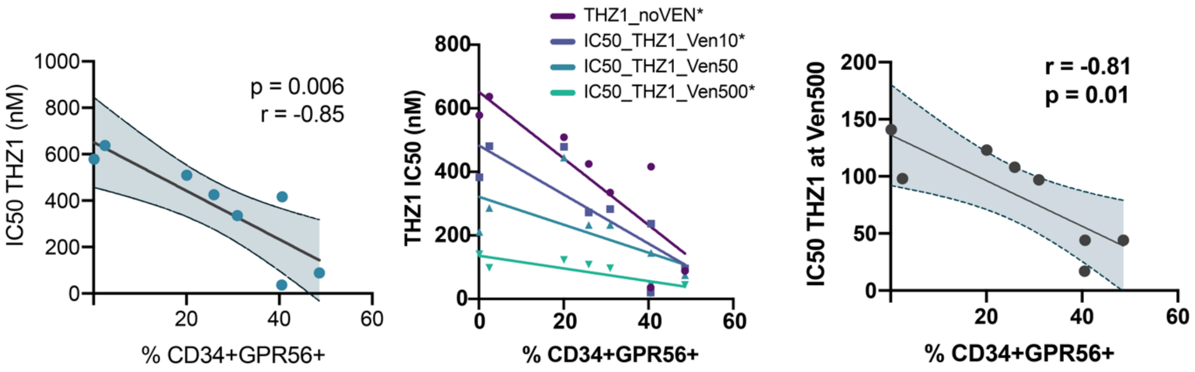

Fig. R6. *Left:* significant anti-correlation (Pearson) between the percentage of CD34+GPR56+ cells in an AML sample and the corresponding IC50 for THZ1 shows that AML samples with a high CD34+GPR56+ fraction grow more dependently on pathways hit by THZ1. *Middle*: Addition of increasing doses of venetoclax flatten the regression curves. Asterisks in the legend indicate significant correlation. *Right*: significant anti-correlation between the percentage of CD34+GPR56+ cells in 8 AML samples and the IC50s for THZ1 in presence of 500nM venetoclax (Pearson).

with the old and new in vivo drug treatment experiment (**see below under point 5**), in which we found that THZ1 and the more specific CDK7i, CT7001, suppressed CD34+GPR56+ more than the other cells (**Fig. R6, rev_Fig 5E, Appendix Fig S6E**).

5. What is the anti-leukemia efficacy of the combination therapy as opposed to the monotherapy with THZ1 in vivo? Can this combination completely delete GRP56+ populations?

This was an important question given that our synergism experiments were only performed *in vitro*. At the same time, drug combination treatment in preclinical models is highly challenging and ideally uses optimized compounds and treatment regimens. Given the time constraint for

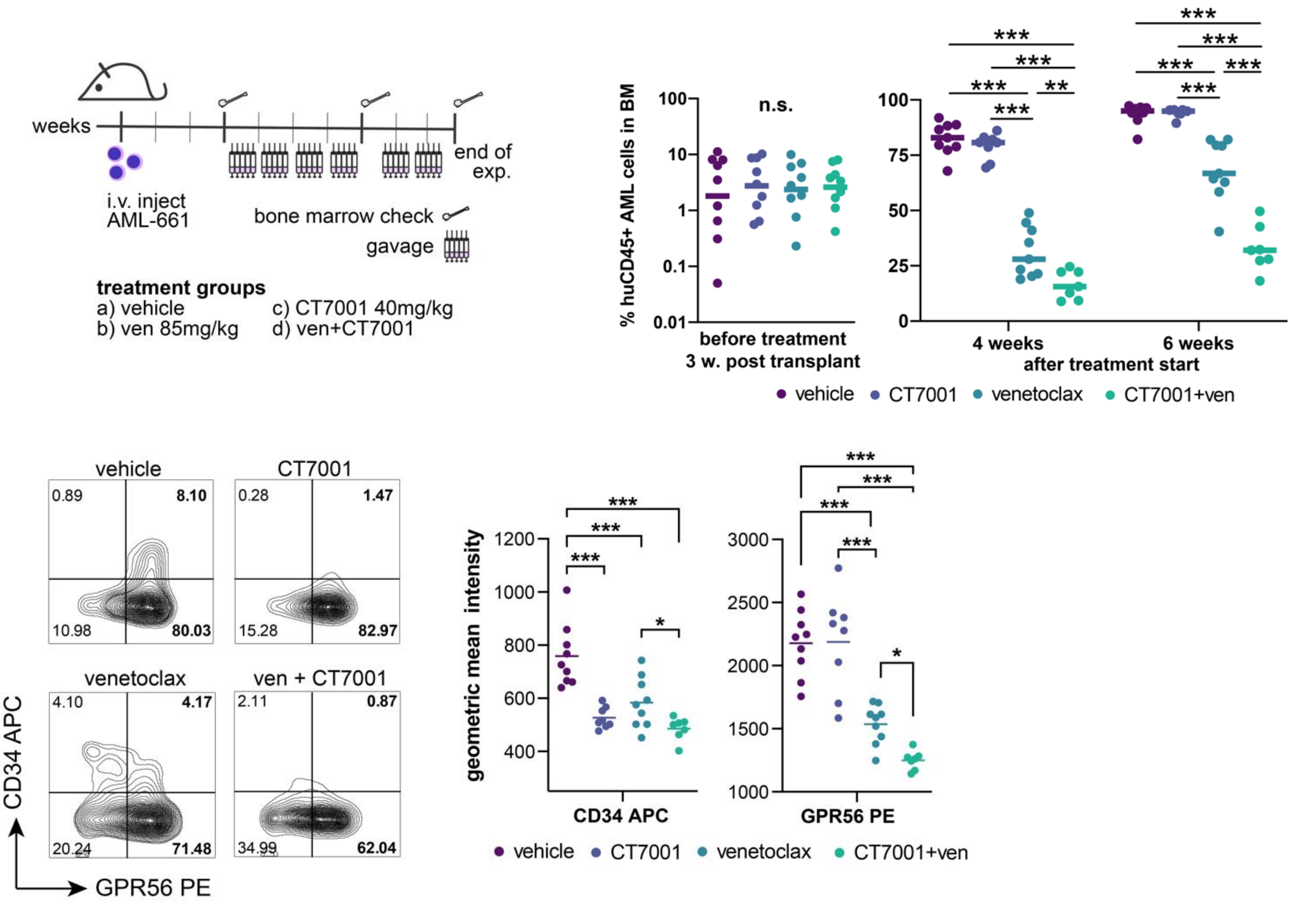

Fig. R7. *Upper left*: setup of the in vivo combined drug treatment experiment. NSG mice were injected with 10^5 AML 661 cells. Three weeks post injection bone marrow (BM) was analyzed for human leukemic engraftment by BM aspiration. Treatment with vehicle, CT7001, VEN or the combination of CT7001 and VEN was started in the following week as indicated. BM was analyzed again after the end of the 4-week and 6-week treatment periods. Unpaired t-test. *Upper right*: Overall human leukemic engraftment in mice before and at the end of the 4- and 6-week treatment period. Individual mice and mean engraftment are shown. Unpaired t-test. *Lower left:* representative FACS plots showing CD34 and GPR56 expression in engraftment AML cells after the 4-week treatment period. *Lower right:* comparison of the geometric mean intensity of CD34 APC (left) and GPR56 PE (right) in the four treatment groups. The mean intensity reflects the number of molecules on the surface of a cell.

the revision we relied on published data for the dosage and regimen of venetoclax (85mg/kg). As Venetoclax has to be applied by gavage, we searched for a CDK7 inhibitor that could a) also be applied by gavage to reduce the burden for the mice (THZ1 has to be applied 2x daily by i.p. injection), and b) had a higher chance to be used in the clinic than THZ1. We finally chose the orally applicable CDK7i CT7001 (Samuraciclib), which is already being tested in clinical trials for advanced solid malignancies (ClinicalTrials.gov Identifiers: NCT03363893 and NCT04802759). We also chose a more aggressive PDX sample for this in vivo experiment (PDX AML-661) to address the concern raised by referee#2 that the in vivo experiment with THZ1 was only performed with one PDX (04H112). We soon noticed

that the chosen dose of CT7001 (80mg/kg), which was even below the published maximum dose applied in NSG mice was too toxic (Clark *et al*, 2017). We therefore reduced the dose by half (40mg/kg), while maintaining venetoclax at 85mg/kg. This relatively strict dose reduction might be the cause, why CT7001 showed only little effect on overall leukemia engraftment when applied alone. At the same time, this low dose allowed us to unequivocally confirm synergism with venetoclax, as the drug combination always yielded stronger reduction of the leukemia burden than obtained with the hypothetical sum of effects achieved with each compound alone (**Fig. R7, rev_Fig 6C-F**). Although overall engraftment was not reduced, there was a significant reduction in the CD34+GPR56+ compartment with CT7001 alone similar to what we had observed before with THZ1 as monotherapy. These results are in line with the anti-correlation between IC50s for THZ1 and the percentage of CD34+GPR56+ cells in an AML sample (**Fig. R6**).

Minor comments:

- AML affects both young and elderly people... (line 60)

We completely agree with the referee and changed this on page 2, line 62.

- In Figure 1B, does {greater than or equal to}15 mean 15 {less than or equal to} AML < 30? and dose {greater than or equal to}1 mean 1 {less than or equal to} AML < 15? (make it clearer)

We realized that this labeling was confusing: greater than 15 includes also greater than 30, greater than 1 includes also greater than 15 or 30. It shows that the conserved peaks (shared by at least 30 samples) are more located in promoter regions, while when we are not restrictive at all, i.e. we include basically all ATAC-seq peaks detected in this study (found in at least one sample) the fraction of peaks located at intergenic and intronic regions increases. We rephrased the labeling accordingly.

- In Figure 2b-d, it seems that shGRP56 transduced CB CD34+ cells (GFP+ cells) were not sorted before transplantation.

This is correct. Sorting primary infected cells bears the risk of damaging the cells through shear stress (drop in viability in the following hours post sorting) and usually causes a considerable loss of cells. We therefore decided to not sort the cells. Moreover, the engraftment of non-transduced cells serves as a powerful technical control: when a knockdown condition has an engraftment disadvantage, the successful engraftment of non-transduced cells confirms that there were no technical issues such as injection failures or contaminations. To make the conditions still comparable, we pre-tested gene transfer with the different virus batches to make sure that gene transfer was highly similar: as shown in **rev_Fig 1D and Fig. R8**, the percentage of GFP was 40-50% (no significant difference) in all three conditions (data in this figure were taken from the CD34+ *in vivo* experiment). We added a statement on this **on page 8, line 197**.

- In Figure 2a and Supplements Figure 2b, delta GFP% could be affected by the baseline transduction efficiency. It might be better to show fold changes?

We agree with the referee that delta GFP% might be misleading when the starting gene transfer is not very similar in different conditions.

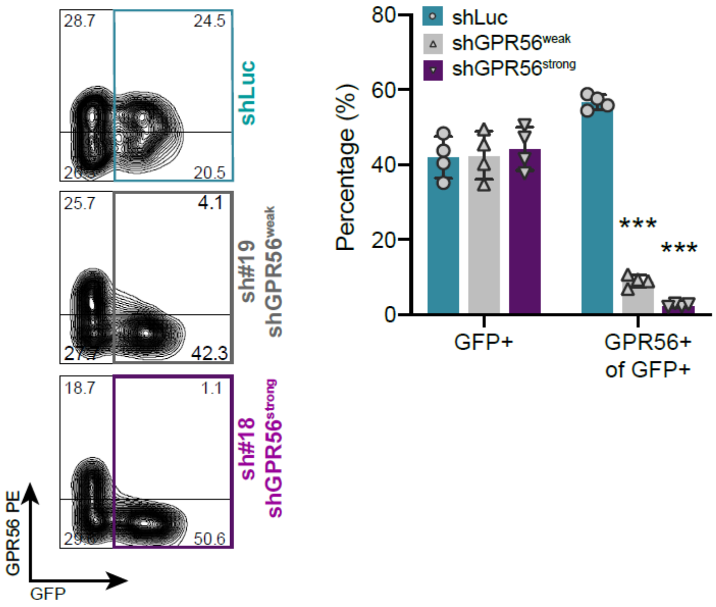

Fig. R8. Knockdown efficiency of two shRNAs against *GPR56* (shGPR56^{weak} and shGPR56^{strong}) versus shLuc as negative control measured on protein level by flow cytometry in CD34^{+} CB cells. Shown are representative FACS plots (left) and the percentage of GPR56^{+} cells of transduced GFP^{+} cells (right panel). Note that the overall percentage of successfully transduced GFP+ cells is highly similar in all three conditions (40-50%). These cells were also used for the *in vivo* experiment.

The CD34+ CB experiment was analyzed in FACS tubes, so that only percentages and not cell counts were assessed (**rev_Fig 2A**). However, the same transduced cells were subsequently sorted directly into methyl cellulose using a precisely determined number of 250 GFP+ cells per dish. As can be seen in **rev_Fig 2B**, the number of colonies generated from the exact same number of starting GFP+ cells was highly reduced upon GPR56 KD.

The experiment with the eight AML cell lines was performed in an HTS-FACS format, which also registers cell counts. We were therefore able to calculate the fold-changes of GFP+ cells normalized to day2. This analysis revealed similar results compared with the original analysis. In particular, it became even more evident that Kasumi cells, which harbor a t(8;21) that is associated with absence of GPR56 expression in primary human AML, were not affected by GPR56 KD (**Fig. R9, Appendix Fig S2B**). In the cell lines with mutations associated with very high GPR56 expression in primary human AML (FLT3-ITD (MV4-11)

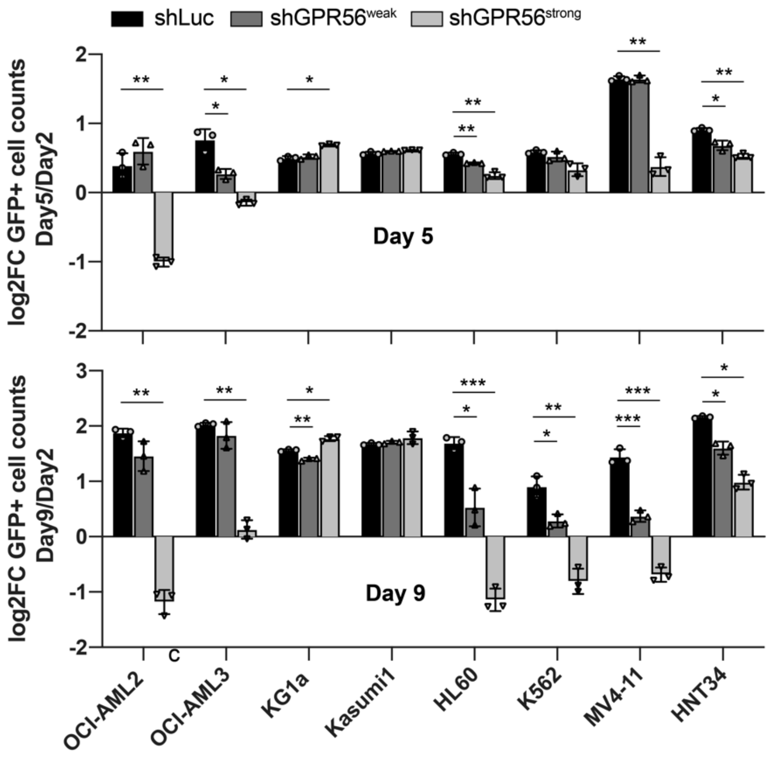

Fig. R9. Knockdown of GPR56 in 8 leukemia cell lines with shGPR56^{weak} and shGPR56^{strong} versus shLuc control. Fold-change of the GFP^{+} cell counts on day 5 (top) and day 9 (bottom) compared with day 2 are shown. Three replicate wells were monitored by HTS-FACS per condition. Multiple t-tests, ns: not significant, * $p<0.05$, ** $p<0.005$, *** $p<0.0005$.

and EVI1 (HNT34)), the negative impact of GPR56 KD became also more evident. We thank the reviewer for this valuable suggestion and replaced the old by the new figure.

- In Supplemental Figure 2c, do these graphs mean that most engrafted leukemia cells are GFP negative in both shLuc and shGRP56 condition?

We realized that the axis label in Suppl. Fig. 2c was misleading. By indicating “% AML cells with GFP/AM negative”, we meant the percentage of overall human leukemic engraftment comprising GFP or Ametrine (AM) positive **and** negative cells. As mentioned above, the engraftment of non-transduced cells is helpful in experiments, where the transduced cells have a strong disadvantage in engraftment as seen for GPR56 knockdown cells. The very

good engraftment of non-transduced GFP/Ametrine negative cells proves that there were no technical issues during injection in the GPR56 knockdown conditions. The gene transfer before injection is shown in **rev_Fig 2E** and **Dataset EV9**. We changed the axis label in **Appendix Fig S2C** accordingly.

- The reduced adhesion and deformation capacity in GRP56 KD K562 cells are shown in Figure 2d-e (p.8). Is there any correlation to LSC properties?

We agree with the reviewer that the connection to LSCs was not clearly formulated in the previous version. In the revised manuscript, we emphasized that the adhesion assays were established and validated to reflect the adhesion properties of primary human hematopoietic stem and leukemia blasts (Burk *et al*, 2015) (**page 9, lines 226-227**). The reason for choosing the K562 cell line for these tests was that the GPR56 knockdown hampered viability and expansion of primary human cells so rapidly that the number and quality of transduced cells was not sufficient for performing these experiments including all replicates. Furthermore, the hampered engraftment caused by GPR56 suppression, led us hypothesize that GPR56 suppression should induce an impairment in cytoadhesion capacity to the bone marrow niche. We verified this hypothesis by using two biophysical techniques: (i) the label-free determination of tight contact area from live cell microinterferometry images and (ii) the quantitative assessment of mechanical strength of cytoadhesion by using the self-developed, high throughput assay utilizing pressure waves. We found that GPR56 suppression resulted in significant decreases in (i) the contact area by a factor of 1.9 ($p = 1.0 \times 10^{-10}$, $N > 70$) and (ii) the critical pressure for cell detachment by a factor of 1.2 ($p < 0.05$, $N > 2000$). Moreover, by tracking the periphery of cells undergoing active deformation, we found that GPR56 suppression significantly suppressed the active deformation, which can be attributed to the impaired actin dynamics and hence RhoA activity. To improve clarity, we moved the results from the tight adhesion area from supplemental information to the main figure **rev_Fig 2F** and added text as mentioned above.

- In Figure 3: some of the experiments were done by using HEK293T or RPE cells, not AML cells...Reduced ciliation in sgGRP56 RPE-1 cells is shown, through SMO suppression. What is the effect of SMO regulation by GRP56 in AML cells?

This is a an important question. However, the primary cilium is difficult to visualize in suspension cells, because it forms a rudimentary short structure in these cells rather than an elongated "line" as seen in adhesion cells when using standard fluorescence imaging. From published work we know that inhibition of SMO with the SMO inhibitor PF-04449913 (PF-913) impairs AML development *in vivo* by shifting quiescent cells from G0 back to the cell cycle (Fukushima *et al*, 2016). We used the RPE-1 cell line for technical reasons to be able

to visualize the subcellular compartment where the Hh pathway takes place. In addition, the fact that GPR56 knockdown suppresses *SMO* on mRNA level also in RPE-1 cells provides evidence that GPR56 affects the Hedgehog/SMO axis independent of the cellular context.

- In Figure 6, reduced MCL-1 expression is nominated as an advantage in the combination therapy. Is MCL-1 regulated by GRP56? Or is it an independent factor to the resistance to the therapy?

This is a pivotal and interesting question. To answer this, we performed additional Western blots after GPR56 knockdown in the AML cell line HEL. We found increased Mcl-1 and slightly decreased Bcl-2 and Bcl-xL levels upon GPR56 knockdown (**Fig R.5, Appendix Fig S6D**). Given the strong disadvantage and apoptosis induction upon GPR56 knockdown, we cannot rule out that the upregulation of Mcl-1 is a compensatory mechanism of the cells.

At the same time, this experiment clearly shows that the Mcl-1 suppressive activity of THZ1 is most likely independent of GPR56. In support of this, we showed before that the CDK12/13 inhibitor THZ531 also strongly suppressed Mcl-1, while the more specific CDK7i YKL-5-124 had little to no effect. These results suggest that the strong Mcl1 suppressive activity of THZ1 might rather come from its CDK12/13 inhibitory activity. These results also explain why GPR56 overexpression is not sufficient to rescue the effect of THZ1 on AML cell proliferation (**see also answer to point 3**).

Referee #2 (Comments on Novelty/Model System for Author):

State of the art molecular techniques

Referee #2 (Remarks for Author):

This manuscript hypothesizes that AML (stem cells) may be able to escape therapy by oscillating between two states, a GRP56highCD34+ and a GRP56lowCD34-. GRP56 interacts in the different cell states with different signalling pathways at receptor level, specifically with WNT signalling via lpr6 co-receptor and Hedgehog signalling via SMO. The authors claim that activation of WNT signalling shifts the balance between the two cell states towards the GPP56highCD34low phenotype.

The conclusion of this paper is that GRPhigh leukemic stem cells need to be targeted upstream of GPR signalling by targeting Rho (via inhibition of CDK7) and the Wnt pathway (by inhibition of CDK12/13). As CDK12/13 inhibition also suppresses MCL-1 there is synergy between CDK12/13 and BCL2 inhibition (Venetoclax).

I am intrigued by this model but I think there are still some open ends that need to be addressed.

Key points:

> I found the manuscript very difficult to read and needed to make my own drawings to understand the interaction between the different pathway. There are also way too many abbreviations. More data should go in the supplements, also to make the figures less loaded with data.

We thank the referee for this comment on how to improve readability and clarity of our manuscript. To address these concerns, we took the following measures:

- in **rev_Fig 1D** we moved the 2nd part of FACS plots to Appendix Fig S1A
- we completely removed previous supplemental Fig. S1D and S1F and the corresponding text, as they were not essential to the message
- we show only one IGV example (VWF) in the main **rev_Fig1E**
- we completely revised Figures 4-6 and corresponding Appendix Figures S4-S6. These contain the following changes:
    - less subfigures, e.g. **previous Fig. 4a, b, c, f, h were moved to Appendix Fig S4**, less gene examples are shown in rev_Fig4A compared to previous Fig 4d-e)
    - less but larger cartoons to explain the suggested mechanisms (**rev_Fig 4B, rev_Fig 4F, rev_Fig 6G**)
    - we completely removed previous Fig. 6g-I (aspect on Rho inhibitors) to create space for the new *in vivo* experiment and also to reduce the different classes of inhibitors used. Now there is a clear focus on CDK7 inhibitors. This also simplified the model in **rev_Fig 6G**
    - we removed the YKL-5-124 results from Fig 6A, but kept the data in Dataset EV12 to reduce the figure load

> An overall figure (and not just tiny subfigures) explaining the proposed interaction of GPR56 with the other pathways and where the many inhibitors hit these pathways as well as a figure explaining the model of the oscillating stem cell states would be essential to make this work comprehensible for the nonspecialist.

We thank the referee for this suggestion on how to improve the cartoons to visualize where the compounds used interact with the GPR56-regulated pathways. We completely removed the tiny subfigures in Figure 4 and instead provided a novel cartoon in **rev_Fig 4B (Fig. R10)**, which visualizes in a simplified way that GPR56 enhances in parallel pathways and genes that are differentially active / expressed in the CD34+GPR56+ versus the CD34-GPR56+ compartments and indicated where and how the compounds used in Figure 4 affect these pathways. We also indicated with arrows in **rev_Fig 4D** and **Appendix Fig S4G** in which direction the indicated compounds affect Wnt and Hh pathways.

We added another cartoon in **rev_Fig 4F** (**Fig. R11**), which simplifies the suggested mechanism of reciprocal inhibition between TGFb/Hh and Wnt pathways, which should

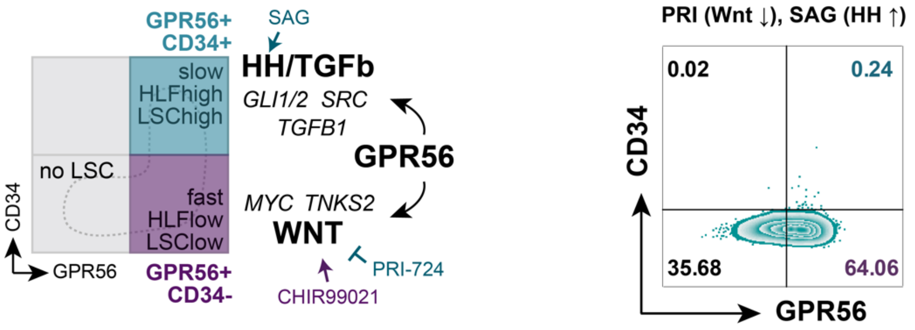

Fig. R10. *Left:* Cartoon visualizing that GPR56 enhances genes and pathways differentially active in the CD34$^+$GPR56$^+$ fraction, which is characterized by slow cell cycle progression, high LSC frequency, and high expression of the stemness gene *HLF* versus the CD34$^-$GPR56$^+$ cells, which are more differentiated (lower LSC frequency), cycle faster, and have little *HLF* expression. Arrows and blocked arrows indicate activation or inhibition by the indicated small molecules, respectively, which were used in subsequent experiments.
Right: one example plot from rev_Fig 4D showing how we used arrows to indicate that PRI-724 suppresses Wnt, while SAG activates HH.

results in reciprocal transition between the compartments (oscillation).

We also modified the final cartoon in **rev_Fig 6G** (**Fig. R11**) to visualized in a more straight-

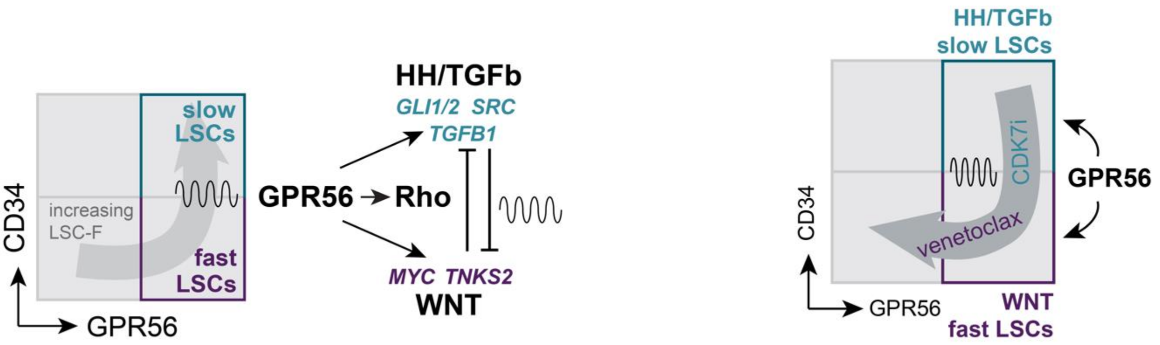

Fig. R11. *Left:* Cartoon (from rev_Fig 4F) visualizing the proposed mechanism by which both GPR56$^+$ LSC enriched compartments are maintained: GPR56 enhances pathways, which reciprocally inhibit each other and are differentially active in the two fractions. This should result in a constant transition between the compartments and thus prevent exhaustion of the two populations. *Right:* Cartoon (from rev_Fig 6G) visualizing how CDK7i and VEN synergize to suppress both GPR56+ compartments in AML.

forward way how the drug combination of CDK7i with venetoclax synergizes in suppressing both GPR56+ compartments.

> My main concern is that the effect of inhibiting/activating Wnt signalling on the GRP56highCD34+ population is only shown in own patient-derived sample (AML661) and although one sees a loss/increase of this population I struggled to see that there is a shift from GRP56highCD34+ to GRP56highCD34- cells of vice versa. Most of the data are inferential rather than providing an actual proof for the oscillation between the different stem cell states.

We agree with the referee that it was important to show that the proposed mechanism was not specific to one AML sample, but could be reproduced and observed also in other samples. We also fully agree that clear evidence for reciprocal transition, in particular the transition from the CD34 negative to the CD34 positive compartment was lacking.

We addressed these concerns in the following ways:

We performed several sorting experiments (see also answer to referee#1 point 2) using several different primary human AML samples from which we sorted the CD34/GPR56 +/+ and -/+ fractions and subsequently exposed them to compounds modulating Hh, Wnt, and TGFb.

a) We showed already in the original version of the manuscript that the Wnt/Ahr agonist CHIR99021 strongly accelerates this differentiation process in bulk AML-491 cells, which was also observed when adding Wnt3a to the media (**now in Appendix Fig S4B-C**). We

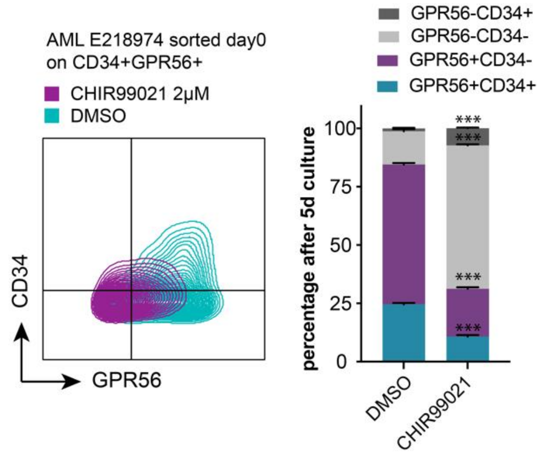

Fig. R12. Contour FACS plots (left) and summary bar graph (right) showing CD34 and GPR56 expression after 5-day culture of purified CD34+GPR56+ cells from AML sample E218974 with CHIR99021 or vehicle DMSO, n=3.

now exposed the purified CD34+GPR56+ cells from AML E218974 to CHIR99021 and observed similar effects as observed with bulk AML-491 (**rev_Fig 4C, Fig. R12**).

b) To provide evidence for the reverse process, we used the Wnt inhibitor PRI-724, the Hh agonist SAG, and recombinant TGFb alone and in combination and added these to either purified CD34+GPR56+ or CD34-GPR56+ cells from two different primary human AML samples (see also answer to referee#1 point 2). We show that the triple combination was

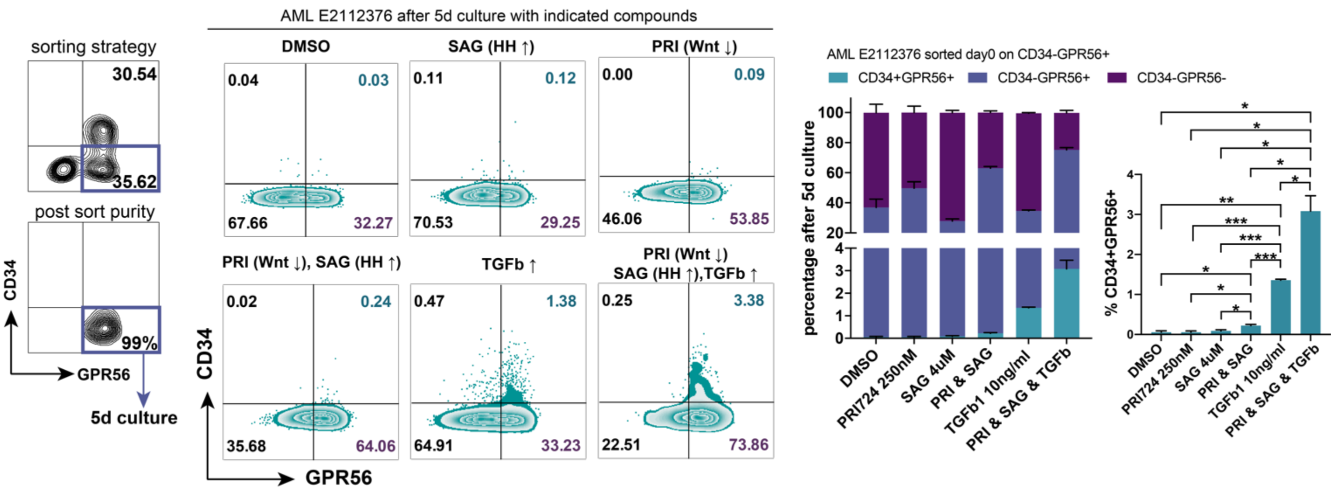

Fig. R13. *Left:* FACS contour plots of AML E2112376 before (top) and after (bottom) sorting of CD34-GPR56+ cells. *Middle:* FACS plots after 5-day treatment of the purified CD34-GPR56+ cells with the indicated compounds or combinations. *Right*: Quantification of percentages of CD34 and GPR56 expressing cells after 5-day treatment with indicated compounds and combinations. N = 3, pairwise t-tests, BH-adjusted p-values.

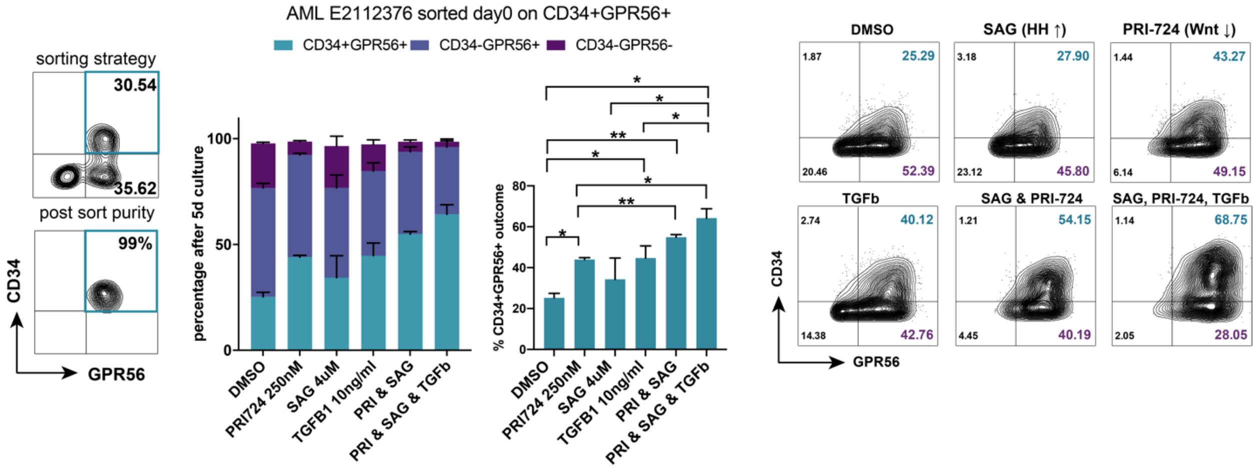

Fig. R14. *Left*: FACS contour plots of AML E2112376 showing the sorting strategy for in vitro treatment before (top) and after (bottom) sorting of CD34+GPR56+ cells. *Middle*: Quantification of percentages of CD34 and GPR56 expressing cells after 5-day treatment with SAG, PRI-724, and TGFb alone or with the indicated combinations. Statistical analysis is visualized only for CD34+GPR56+ output cells. For complete statistical group comparisons see dataset EV11. *Right*: representative FACS plots showing CD34 and GPR56 expression after 5-day treatment with the indicated compounds or combinations. N = 3, pairwise t-tests, BH-adjusted p-values.

most efficient in preventing the differentiation of CD34+GPR56+ to CD34-GPR56- and double negative cells (**Fig. R13, Appendix Fig S4G**). Most importantly, we were able to **re-generate CD34+GPR56+ from CD34-GPR56+ cells** using the combination of the three molecules in these two independent experiments (**Fig. R14, rev_Fig 4D-E,** see **Appendix Fig S4F** for AML E2113590). This was specifically striking, as there is a natural tendency of

CD34+GPR56+ to differentiate to CD34-GPR56+ cells *in vitro*, which can partially be delayed but not reverted by optimizing culture conditions (Pabst *et al*, 2014).

Together, these novel *in vitro* sorting results demonstrate that Wnt inhibition together with Hh and TGFb pathway activation enable the transition from the more differentiated CD34-GPR56+ to the more immature CD34+GPR56+ state. These results complement our *in vivo* experiment from Figure 5, where we showed already before that the CD34+GPR56+ compartment was replenished in mice after drug withdrawal, in which THZ1 had completely suppressed this fraction at an earlier time point (**now rev_Fig 5E**).

c) We performed another *in vivo* drug treatment experiment to answer question 5 by

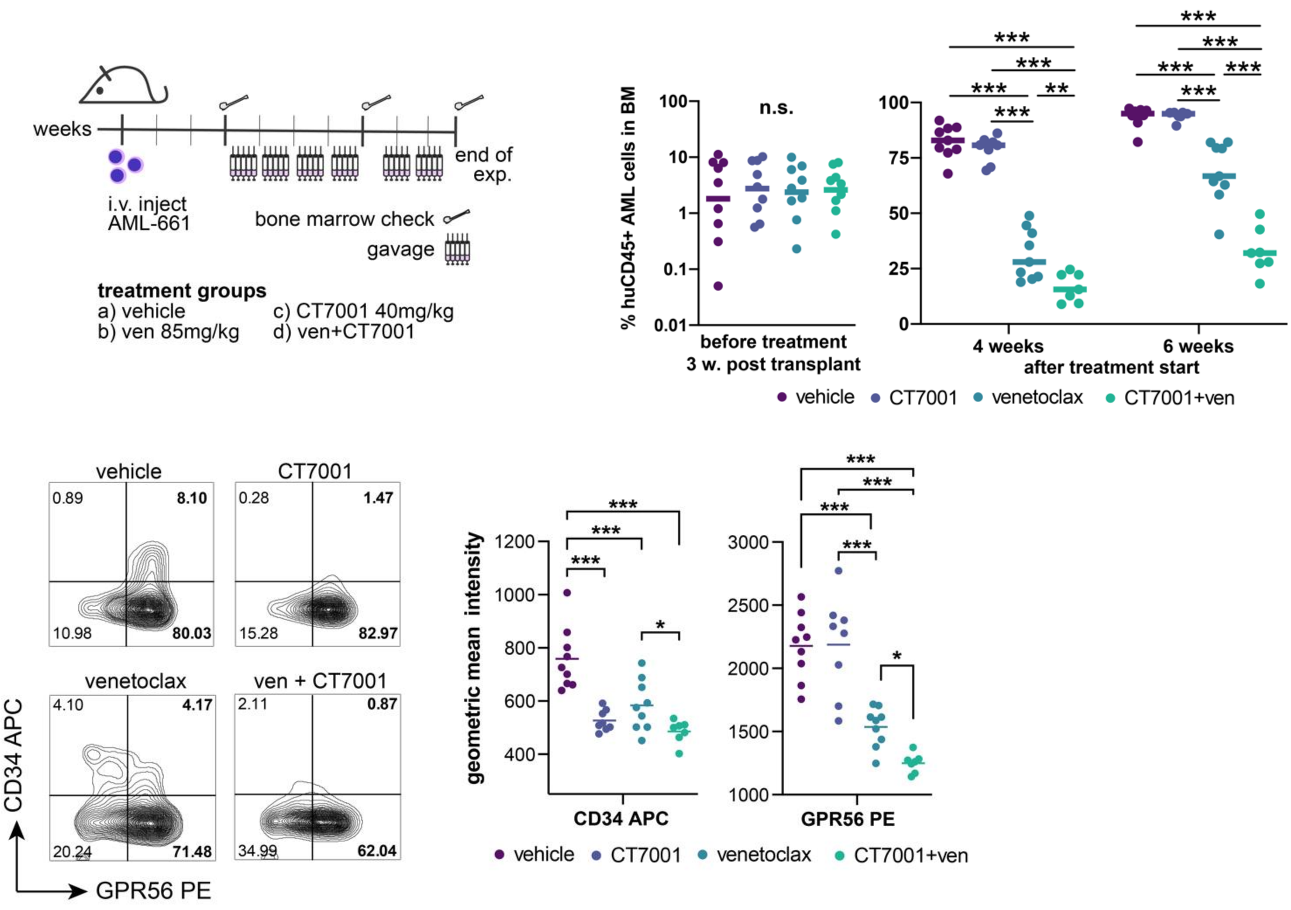

Fig. R15. *Upper left*: setup of the in vivo combined drug treatment experiment. NSG mice were injected with 10^5 AML 661 cells. Three weeks post injection bone marrow (BM) was analyzed for human leukemic engraftment by BM aspiration. Treatment with vehicle, CT7001, VEN or the combination of CT7001 and VEN was started in the following week as indicated. BM was analyzed again after the end of the 4-week and 6-week treatment periods. Unpaired t-test. *Upper right*: Overall human leukemic engraftment in mice before and at the end of the 4- and 6-week treatment period. Individual mice and mean engraftment are shown. Unpaired t-test. *Lower left:* representative FACS plots showing CD34 and GPR56 expression in engraftment AML cells after the 4-week treatment period. *Lower right:* comparison of the geometric mean intensity of CD34 APC (left) and GPR56 PE (right) in the four treatment groups. The mean intensity reflects the number of molecules on the surface of a cell.

referee#1. In this novel drug experiment we used the highly aggressive **PDX-AML sample (AML-661, 2nd relapse, Fig. R15, rev_Fig 6C-F**), while in the previous *in vivo* drug treatment experiment, in which we used THZ1 as monotherapy, we had used the *de novo* AML **PDX**

AML-04H112. We were able to provide *in vivo* evidence of synergism between CDK7i and venetoclax *in vivo* in this additional AML sample. Three more primary human AML samples were also used in **rev_Fig4 and Appendix Fig S4F** for functional tests (E218974, E2112376, E2113590), which were also used for the synergism experiment in **rev_Fig 6A**.

References

Burk AS, Monzel C, Yoshikawa HY, Wuchter P, Saffrich R, Eckstein V, Tanaka M & Ho AD (2015) Quantifying adhesion mechanisms and dynamics of human hematopoietic stem and progenitor cells. *Sci. Rep.* **5:** 9370

Clark K, Ainscow E, Peall A, Thomson S, Leishman A, Elaine S, Ali S, Coombes R, Barrett A & Bahl A (2017) CT7001, a Novel Orally Bio-Available CDK7 Inhibitor, Is Highly Active in in-Vitro and in-Vivo Models of AML. *Blood*

Fukushima N, Minami Y, Kakiuchi S, Kuwatsuka Y, Hayakawa F, Jamieson C, Kiyoi H & Naoe T (2016) Small-molecule Hedgehog inhibitor attenuates the leukemia-initiation potential of acute myeloid leukemia cells. *Cancer Sci.* **107:** 1422–1429

Pabst C, Krosl J, Fares I, Boucher G, Ruel R, Marinier A, Lemieux S, Hébert J & Sauvageau G (2014) Identification of small molecules that support human leukemia stem cell activity ex vivo. *Nat. Methods* **11:** 436–442

26th Jan 2022

Dear Dr. Pabst,

Thank you for the submission of your revised manuscript to EMBO Molecular Medicine. We have now received the enclosed report from the two referees who were asked to re-assess it. As you will see, the referees are now overall supportive, and I am pleased to inform you that we will be able to accept your manuscript pending the following amendments:

1. Please address the remaining minor concerns of Referee #2, especially Point #2, to make the manuscript more accessible to the general audience of EMBO Molecular Medicine. Please reply to Point #1; experimental analysis in this regard is not mandatory for the acceptance of the manuscript.

On a more editorial level, please do the following:

I look forward to reading a new revised version of your manuscript as soon as possible.

Kind regards,
Jingyi

Jingyi Hou
Editor
EMBO Molecular Medicine

*** Instructions to submit your revised manuscript ***

*** PLEASE NOTE *** As part of the EMBO Publications transparent editorial process initiative (see our Editorial at https://www.embopress.org/doi/pdf/10.1002/emmm.201000094), EMBO Molecular Medicine will publish online a Review Process File to accompany accepted manuscripts.

In the event of acceptance, this file will be published in conjunction with your paper and will include the anonymous referee reports, your point-by-point response and all pertinent correspondence relating to the manuscript. If you do NOT want this file to be published, please inform the editorial office at contact@embomolmed.org.

To submit your manuscript, please follow this link:

https://embomolmed.msubmit.net/cgi-bin/main.plex

When submitting your revised manuscript, please include:

1) a .docx formatted version of the manuscript text (including Figure legends and tables)

2) Separate figure files*

3) supplemental information as Expanded View and/or Appendix. Please carefully check the authors guidelines for formatting Expanded view and Appendix figures and tables at https://www.embopress.org/page/journal/17574684/authorguide#expandedview

4) a letter INCLUDING the reviewer's reports and your detailed responses to their comments (as Word file).

5) The paper explained: EMBO Molecular Medicine articles are accompanied by a summary of the articles to emphasize the major findings in the paper and their medical implications for the non-specialist reader. Please provide a draft summary of your article highlighting
- the medical issue you are addressing,
- the results obtained and
- their clinical impact.
This may be edited to ensure that readers understand the significance and context of the research.
Please refer to any of our published articles for an example.

6) For more information: There is space at the end of each article to list relevant web links for further consultation by our readers. Could you identify some relevant ones and provide such information as well? Some examples are patient associations, relevant databases, OMIM/proteins/genes links, author's websites, etc...

7) Author contributions: the contribution of every author must be detailed in a separate section.

8) EMBO Molecular Medicine now requires a complete author checklist (https://www.embopress.org/page/journal/17574684/authorguide) to be submitted with all revised manuscripts. Please use the checklist as guideline for the sort of information we need WITHIN the manuscript. The checklist should only be filled with page numbers were the information can be found. This is particularly important for animal reporting, antibody dilutions (missing) and exact values and n that should be indicted instead of a range.

9) Every published paper now includes a 'Synopsis' to further enhance discoverability. Synopses are displayed on the journal webpage and are freely accessible to all readers. They include a short stand first (maximum of 300 characters, including space) as well as 2-5 one sentence bullet points that summarise the paper. Please write the bullet points to summarise the key NEW findings. They should be designed to be complementary to the abstract - i.e. not repeat the same text. We encourage inclusion of key acronyms and quantitative information (maximum of 30 words / bullet point). Please use the passive voice. Please attach these in a separate file or send them by email, we will incorporate them accordingly.

You are also welcome to suggest a striking image or visual abstract to illustrate your article. If you do please provide a jpeg file 550 px-wide x 400-px high.

10) A Conflict of Interest statement should be provided in the main text

11) Please note that we now mandate that all corresponding authors list an ORCID digital identifier. This takes <90 seconds to complete. We encourage all authors to supply an ORCID identifier, which will be linked to their name for unambiguous name identification.

Currently, our records indicate that the ORCID for your account is 0000-0001-9716-5909.

Please click the link below to modify this ORCID:
Link Not Available

12) The system will prompt you to fill in your funding and payment information. This will allow Wiley to send you a quote for the article processing charge (APC) in case of acceptance. This quote takes into account any reduction or fee waivers that you may be eligible for. Authors do not need to pay any fees before their manuscript is accepted and transferred to our publisher.

*Additional important information regarding Figures

Each figure should be given in a separate file and should have the following resolution:
Graphs 800-1,200 DPI
Photos 400-800 DPI
Colour (only CMYK) 300-400 DPI"

Figures are not edited by the production team. All lettering should be the same size and style; figure panels should be indicated by capital letters (A, B, C etc). Gridlines are not allowed except for log plots. Figures should be numbered in the order of their appearance in the text with Arabic numerals. Each Figure must have a separate legend and a caption is needed for each panel.

*Additional important information regarding figures and illustrations can be found at https://bit.ly/EMBOPressFigurePreparationGuideline. See also figure legend preparation guidelines: https://www.embopress.org/page/journal/17574684/authorguide#figureformat

The system will prompt you to fill in your funding and payment information. This will allow Wiley to send you a quote for the article processing charge (APC) in case of acceptance. This quote takes into account any reduction or fee waivers that you may be eligible for. Authors do not need to pay any fees before their manuscript is accepted and transferred to our publisher.

***** Reviewer's comments *****

Referee #1 (Remarks for Author):

The revised manuscript is substantially improved and I thank the authors for their careful attention to the reviews.

Referee #2 (Remarks for Author):

The manuscript has been significantly improved. In particular, there are now more data showing that the slowcycling CD34highGPR56high LSC population can be regenerated from CD34lowGPR56high cells. There is solid evidence that GPR56 plays a key role in maintaining leukemic stem cells. There is also ample evidence how GPR56 regulates Hh and Wnt signallinging and affects EMT gene expression. And finally there are good pre-clinical data on how GPR56 may be therapeutically targeted via CDK7 inhibtion.

Minor points:

> I still wonder whether it would have been useful to transplant highly purified CD34negativeGPR56high AML blasts at limiting

dilution onto immunodeficient mice to demonstrate plasticity of the GPR56 compartment and reconstitution with CD34highGPR5high LSC.

> I am slightly dissappointed that the authors have not picked up on the advice to make the manuscript more readable for the non-specialist. There are still way to many abbreviations and overloaded figures that will be difficult to read.

> I also still think that a proper "graphical summary" would make the paper easier accessible for a broader audience.

However, in general an important and thorough study that warrents publication.

EMM-2021-14990 "CDK7/12/13 inhibition targets an oscillating LSC network and synergizes with venetoclax in AML"

Point-by-point answers to the reviewers

Referee #1 (Remarks for Author):

The revised manuscript is substantially improved and I thank the authors for their careful attention to the reviews.

We thank Referee #1 for the favorable comments on the revised version.

Referee #2 (Remarks for Author):

The manuscript has been significantly improved. In particular, there are now more data showing that the slowcycling CD34highGPR56high LSC population can be regenerated from CD34lowGPR56high cells. There is solid evidence that GPR56 plays a key role in maintaining leukemic stem cells. There is also ample evidence how GPR56 regulates Hh and Wnt signallinging and affects EMT gene expression. And finally there are good pre-clinical data on how GPR56 may be therapeutically targeted via CDK7 inhibtion.

Minor points:

> I still wonder whether it would have been useful to transplant highly purified CD34negativeGPR56high AML blasts at limiting dilution onto immunodeficient mice to demonstrate plasticity of the GPR56 compartment and reconstitution with CD34highGPR5high LSC.

We would like to thank Referee #2 for the particular interest in this topic. Such sorting experiments have been performed and published before (Pabst et al., Blood 2016). The output of GPR56 and CD34 pos/neg cells from the purified fractions was shown only for the *in vitro* experiment and revealed that in some samples a low number of CD34+ cells was generated from GPR56+CD34- cells during 7 days *in vitro* in optimized culture conditions. For the *in vivo* experiments with sorted fractions we had only analyzed the overall leukemic engraftment at that time, but we had already noticed that the CD34+ percentage in some of the engrafted mice was similar between mice injected with CD34-GPR56+ and CD34+GPR56+ cells (one such example is shown in **Fig. R2_01**). We did not understand the mechanism behind these observations and did not follow up on these at that time, as the focus of this earlier study was the differences in leukemia stem cell frequencies in the sorted fractions. It took many years of experiments contained in this study to finally understand the mechanism underlying these observations.

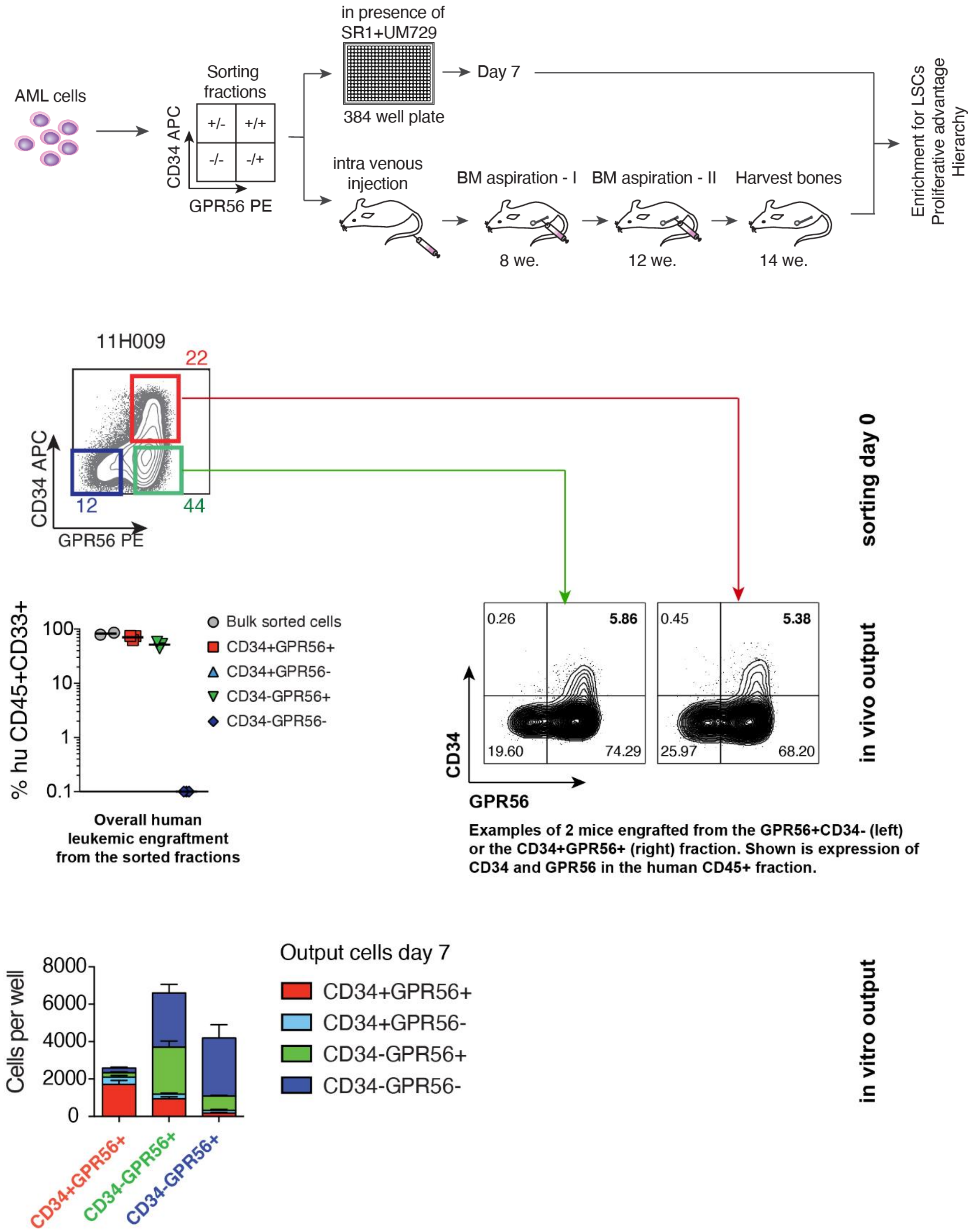

Fig._R2_01. These are modified subfigures from Pabst et al., Blood 2016 including a new subfigure in the middle right.
Top panel: see Pabst, Blood 2016, Fig. 2A: experimental setup of sorting experiments.
Bottom panel: sorting strategy for AML 11H009. Middle: overall leukemic engraftment (left) and CD34/GPR56 profiles from two mice engrafted from CD34+GPR56+ (red) or CD34-GPR56+ (green) cells. Bottom: Output of CD34/GPR56 pos/neg cells from sorted fractions 7 days after in vitro culture.

> I am slightly dissappointed that the authors have not picked up on the advice to make the manuscript more readable for the non-specialist. There are still way to many abbreviations and overloaded figures that will be difficult to read.

We are sorry that we have not managed to meet the referee´s expectations concerning the text editing. Some more precise examples of what we could have changed might have clarified our misunderstandings. We previously replaced the abbreviation “KD” by

"suppression" to make the reading more fluent in the revised version. We also removed a big part of the computational analysis contained in Appendix Figure S1 and corresponding text passages, as these were quite difficult to understand for non-computational experts. Furthermore, we simplified the cartoons and added more cartoons to highlight the conclusions in Figures 4 and 6.
As these modification were not sufficient, we further simplified the manuscript in the following way:

- in Fig. 3A: we simplified the cartoon to highlight the essential: introduction of the 2 reporter assays: one to detect Wnt, one to detect Rho via Ga12/13, as these 2 assays are mentioned many times throughout the figure and text
- Fig. 3B: we indicated directly in the figure that the full length is ligand-activated, while the truncated GPR56 is constitutively active and therefore needs no ligand for inducing signaling in the reporter assays
- Fig. 3: we moved the two sub-figures on LRP6 to Appendix Figure S3, as this might be information for the Wnt expert only
- we replaced the abbreviation VEN by venetoclax
- we replaced the abbreviation dox by doxycycline

We hope that with these additional modifications we further facilitated reading for non-specialists.

> I also still think that a proper "graphical summary" would make the paper easier accessible for a broader audience.
We thank Referee #2 for this comment. A graphical abstract was part of the revised version. We paste it here as **Fig._R2_02**.

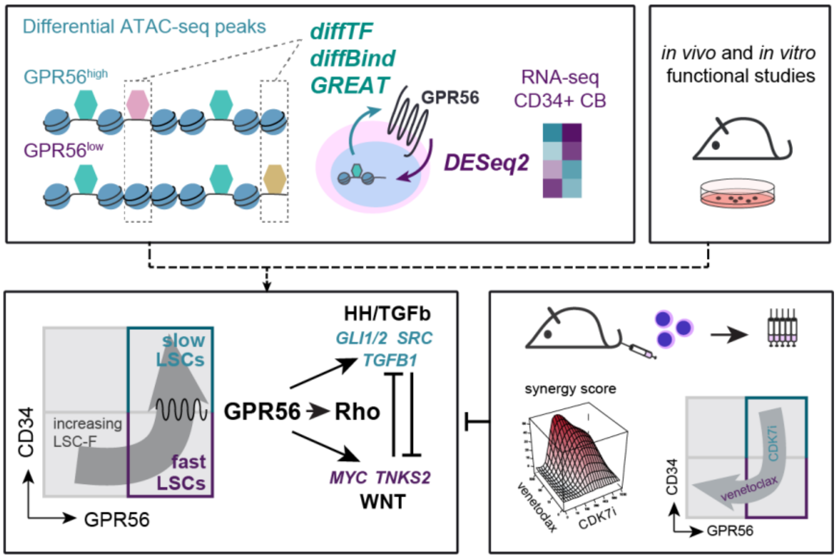

Fig._R2_02. Graphical abstract.
CDK7/12/13 inhibition targets an oscillating LSC network and synergizes with venetoclax in AML
RNA- and ATAC-seq profiling combined with functional *in vitro* and *in vivo* studies unravel the multi-faceted roles of GPR56, a surface marker associated with high leukemia stem cell (LSC) burden and poor prognosis in acute myeloid leukemia (AML).

- ATAC-seq profiling of 35 primary AML specimens links high GPR56 expression to Wnt and Hh signaling.
- GPR56 is required for *in vitro* and *in vivo* expansion of primary human AML cells.
- GPR56 enhances besides RhoA also TGFB, Hedgehog, and Wnt pathway activities, which inhibit each other to coordinate reciprocal transition between the GPR56+CD34+ and GPR56+CD34- compartments to sustain the LSC pool.
- CDK7 inhibitors synergize with the Bcl-2 inhibitor venetoclax to suppress both GPR56^{+} LSC-enriched compartments *in vitro* and *in vivo.*

However, in general an important and thorough study that warrents publication.
We appreciate the referee´s overall supportive opinion concerning publication of our study.

7th Feb 2022

Dear Dr. Pabst,

We are pleased to inform you that your manuscript is accepted for publication and is now being sent to our publisher to be included in the next available issue of EMBO Molecular Medicine.

We would like to remind you that as part of the EMBO Publications transparent editorial process initiative, EMBO Molecular Medicine will publish a Review Process File online to accompany accepted manuscripts. If you do NOT want the file to be published or would like to exclude figures, please immediately inform the editorial office via e-mail.

Please read below for additional IMPORTANT information regarding your article, its publication and the production process.

Congratulations on your interesting work,

Kind regards,
Jingyi

Jingyi Hou
Editor
EMBO Molecular Medicine

Follow us on Twitter @EmboMolMed
Sign up for eTOCs at embopress.org/alertsfeeds

*** *** *** IMPORTANT INFORMATION *** *** ***

SPEED OF PUBLICATION
The journal aims for rapid publication of papers, using the advance online publication "Early View" to expedite the process: A properly copy-edited and formatted version will be published as "Early View" after the proofs have been corrected. Please help the Editors and publisher avoid delays by providing e-mail address(es), telephone and fax numbers at which author(s) can be contacted.

Should you be planning a Press Release on your article, please get in contact with embomolmed@wiley.com as early as possible, in order to coordinate publication and release dates.

LICENSE AND PAYMENT:

All articles published in EMBO Molecular Medicine are fully open access: immediately and freely available to read, download and share.

EMBO Molecular Medicine charges an article processing charge (APC) to cover the publication costs. You, as the corresponding author for this manuscript, should have already received a quote with the article processing fee separately. Please let us know in case this quote has not been received.

Once your article is at Wiley for editorial production you will receive an email from Wiley's Author Services system, which will ask you to log in and will present you with the publication license form for completion. Within the same system the publication fee can be paid by credit card, an invoice, pro forma invoice or purchase order can be requested.

Payment of the publication charge and the signed Open Access Agreement form must be received before the article can be published online.

PROOFS

You will receive the proofs by e-mail approximately 2 weeks after all relevant files have been sent o our Production Office. Please return them within 48 hours and if there should be any problems, please contact the production office at embopressproduction@wiley.com.

Please inform us if there is likely to be any difficulty in reaching you at the above address at that time. Failure to meet our deadlines may result in a delay of publication.

All further communications concerning your paper proofs should quote reference number EMM-2021-14990-V3 and be directed to the production office at embopressproduction@wiley.com.

Thank you,

Jingyi Hou
Editor
EMBO Molecular Medicine

EMBO PRESS

YOU MUST COMPLETE ALL CELLS WITH A PINK BACKGROUND ↓

PLEASE NOTE THAT THIS CHECKLIST WILL BE PUBLISHED ALONGSIDE YOUR PAPER

Corresponding Author Name: Caroline Pabst
Journal Submitted to: EMBO Molecular Medicine
Manuscript Number: EMM-2021-14990

USEFUL LINKS FOR COMPLETING THIS FORM

http://www.antibodypedia.com
http://1degreebio.org
http://www.equator-network.org/reporting-guidelines/improving-bioscience-research-repor
http://grants.nih.gov/grants/olaw/olaw.htm
http://www.mrc.ac.uk/Ourresearch/Ethicsresearchguidance/Useofanimals/index.htm
http://ClinicalTrials.gov
http://www.consort-statement.org
http://www.consort-statement.org/checklists/view/32-consort/66-title
http://www.equator-network.org/reporting-guidelines/reporting-recommendations-for-tumo
http://datadryad.org
http://figshare.com
http://www.ncbi.nlm.nih.gov/gap
http://www.ebi.ac.uk/ega
http://biomodels.net/
http://biomodels.net/miriam/
http://jjj.biochem.sun.ac.za
https://osp.od.nih.gov/biosafety-biosecurity-and-emerging-biotechnology/
http://www.selectagents.gov/

Reporting Checklist For Life Sciences Articles (Rev. June 2017)

This checklist is used to ensure good reporting standards and to improve the reproducibility of published results. These guidelines are consistent with the Principles and Guidelines for Reporting Preclinical Research issued by the NIH in 2014. Please follow the journal's authorship guidelines in preparing your manuscript.

A- Figures
1. Data
The data shown in figures should satisfy the following conditions:

- the data were obtained and processed according to the field's best practice and are presented to reflect the results of the experiments in an accurate and unbiased manner.
- figure panels include only data points, measurements or observations that can be compared to each other in a scientifically meaningful way.
- graphs include clearly labeled error bars for independent experiments and sample sizes. Unless justified, error bars should not be shown for technical replicates.
- if n< 5, the individual data points from each experiment should be plotted and any statistical test employed should be justified
- Source Data should be included to report the data underlying graphs. Please follow the guidelines set out in the author ship guidelines on Data Presentation.

2. Captions

Each figure caption should contain the following information, for each panel where they are relevant:

- a specification of the experimental system investigated (eg cell line, species name).
- the assay(s) and method(s) used to carry out the reported observations and measurements
- an explicit mention of the biological and chemical entity(ies) that are being measured.
- an explicit mention of the biological and chemical entity(ies) that are altered/varied/perturbed in a controlled manner.

- the exact sample size (n) for each experimental group/condition, given as a number, not a range;
- a description of the sample collection allowing the reader to understand whether the samples represent technical or biological replicates (including how many animals, litters, cultures, etc.).
- a statement of how many times the experiment shown was independently replicated in the laboratory.
- definitions of statistical methods and measures:
  - common tests, such as t-test (please specify whether paired vs. unpaired), simple χ2 tests, Wilcoxon and Mann-Whitney tests, can be unambiguously identified by name only, but more complex techniques should be described in the methods section;
  - are tests one-sided or two-sided?
  - are there adjustments for multiple comparisons?
  - exact statistical test results, e.g., P values = x but not P values < x;
  - definition of 'center values' as median or average;
  - definition of error bars as s.d. or s.e.m.

Any descriptions too long for the figure legend should be included in the methods section and/or with the source data.

In the pink boxes below, please ensure that the answers to the following questions are reported in the manuscript itself. Every question should be answered. If the question is not relevant to your research, please write NA (non applicable). We encourage you to include a specific subsection in the methods section for statistics, reagents, animal models and human subjects.

B- Statistics and general methods

Please fill out these boxes ↓ (Do not worry if you cannot see all your text once you press return)

1.a. How was the sample size chosen to ensure adequate power to detect a pre-specified effect size?	For in vivo mouse cohort groups an a priori analysis was performed with the program G*Power (http://www.gpower.hhu.de). For in vitro studies we relied on experience from previous studies.
1.b. For animal studies, include a statement about sample size estimate even if no statistical methods were used.	see 1a. A priori analysis using G*Power software.
2. Describe inclusion/exclusion criteria if samples or animals were excluded from the analysis. Were the criteria pre-established?	Animals which became sick and had to be euthanized for non-leukemia related reasons (e.g. within the days following irradiation or animals which did not wake up after anaesthesia) were excluded from the analysis.
3. Were any steps taken to minimize the effects of subjective bias when allocating animals/samples to treatment (e.g. randomization procedure)? If yes, please describe.	We determined engraftment levels in all mice prior to treatment start and ensured that all treatment groups had comparable engraftment levels before treatment start. Animal staff taking care of the mice did not know about the expected outcome of the experiment.
For animal studies, include a statement about randomization even if no randomization was used.	see above: mice were allocated to treatment groups in a way that there were no differences in the distribution of engraftment levels between treatment groups prior to treatment start.
4.a. Were any steps taken to minimize the effects of subjective bias during group allocation or/and when assessing results (e.g. blinding of the investigator)? If yes please describe.	The experimentator who applied the drugs had to freshly prepare the drugs and therefore was aware of the content of the syringes. The animal caretakers who evaluated the clinical status and signs of disease were blind to the type of treatment.
4.b. For animal studies, include a statement about blinding even if no blinding was done	see above: animal care takers who assessed signs of disease were blind to the treatment. The person who injected the substances freshly prepared each syringe and was therefore aware of the content.
5. For every figure, are statistical tests justified as appropriate?	Yes. We applied t-tests only when normal distribution was tested for or after log-transformation. Otherwise non-parametric tests were used.
Do the data meet the assumptions of the tests (e.g., normal distribution)? Describe any methods used to assess it.	We used the program graph pad prism v.9.0.1. Normality testing is performed using Kolmogorov-Smirnov test.
Is there an estimate of variation within each group of data?	Yes, when means are shown and not the individual replicates, standard deviation is shown in bar graphs.

Is the variance similar between the groups that are being statistically compared?	In multiple testing analyses (2-way ANOVA) no similar variances were assumed, which is recommended by Graph Pad Prism software.

C- Reagents

6. To show that antibodies were profiled for use in the system under study (assay and species), provide a citation, catalog number and/or clone number, supplementary information or reference to an antibody validation profile. e.g., Antibodypedia (see link list at top right), 1DegreeBio (see link list at top right).	Catalog numbers and company names are indicated for each antibody used in this study.
7. Identify the source of cell lines and report if they were recently authenticated (e.g., by STR profiling) and tested for mycoplasma contamination.	Cell lines were freshly purchased as indicated in methods from DSMZ. Mycoplasma contamination is regularly checked and no contamination was detected throughout the study.

* for all hyperlinks, please see the table at the top right of the document

D- Animal Models

8. Report species, strain, gender, age of animals and genetic modification status where applicable. Please detail housing and husbandry conditions and the source of animals.	NOD.Cg-PrkdcscidIl2rgtm1Wjl (NSG) and NOD.Rag1-; ycnull-SGM3 (NRGS) mice were purchased from Jackson Laboratories. NOD.Cg-KitW-41J Prkdcscid Il2rgtm1Wjl/WaskJ (NSGW41) mice were kindly provided by Dr. Claudia Waskow. Female and male mice used in our study were bred and housed in specific pathogen-free animal facilities at the German Cancer Research Center (DKFZ), Heidelberg
9. For experiments involving live vertebrates, include a statement of compliance with ethical regulations and identify the committee(s) approving the experiments.	All animal experiments were approved by offcial comittees (Regierungspräsidium Karlsruhe) and performed in accordance with the regulatory guidelines.
10. We recommend consulting the ARRIVE guidelines (see link list at top right) (PLoS Biol. 8(6), e1000412, 2010) to ensure that other relevant aspects of animal studies are adequately reported. See author guidelines, under 'Reporting Guidelines'. See also: NIH (see link list at top right) and MRC (see link list at top right) recommendations. Please confirm compliance.	All animal experiments were performed in accordance with the regulatory guidelines.

E- Human Subjects

11. Identify the committee(s) approving the study protocol.	The study protocol was approved by the Research Ethics Board of the Medical Faculty of Heidelberg University.
12. Include a statement confirming that informed consent was obtained from all subjects and that the experiments conformed to the principles set out in the WMA Declaration of Helsinki and the Department of Health and Human Services Belmont Report.	Cryopreserved AML patient samples and cord blood units were collected after obtaining written informed consent in accordance with the Declaration of Helsinki and provided according to ethically approved protocols by several biobanks.
13. For publication of patient photos, include a statement confirming that consent to publish was obtained.	NA
14. Report any restrictions on the availability (and/or on the use) of human data or samples.	NA
15. Report the clinical trial registration number (at ClinicalTrials.gov or equivalent), where applicable.	NA
16. For phase II and III randomized controlled trials, please refer to the CONSORT flow diagram (see link list at top right) and submit the CONSORT checklist (see link list at top right) with your submission. See author guidelines, under 'Reporting Guidelines'. Please confirm you have submitted this list.	NA
17. For tumor marker prognostic studies, we recommend that you follow the REMARK reporting guidelines (see link list at top right). See author guidelines, under 'Reporting Guidelines'. Please confirm you have followed these guidelines.	NA

F- Data Accessibility

18: Provide a "Data Availability" section at the end of the Materials & Methods, listing the accession codes for data generated in this study and deposited in a public database (e.g. RNA-Seq data: Gene Expression Omnibus GSE39462, Proteomics data: PRIDE PXD000208 etc.) Please refer to our author guidelines for 'Data Deposition'.<br><br>Data deposition in a public repository is mandatory for:<br>a. Protein, DNA and RNA sequences<br>b. Macromolecular structures<br>c. Crystallographic data for small molecules<br>d. Functional genomics data<br>e. Proteomics and molecular interactions	RNA-seq data of GPR56 KD in CD34: GSE150175, ATAC-seq data on primary AML samples: GSE150868.
19. Deposition is strongly recommended for any datasets that are central and integral to the study; please consider the journal's data policy. If no structured public repository exists for a given data type, we encourage the provision of datasets in the manuscript as a Supplementary Document (see author guidelines under 'Expanded View' or in unstructured repositories such as Dryad (see link list at top right) or Figshare (see link list at top right).	Detailed sample characteristics, source data, and analyses are provided in EV Datasets.
20. Access to human clinical and genomic datasets should be provided with as few restrictions as possible while respecting ethical obligations to the patients and relevant medical and legal issues. If practically possible and compatible with the individual consent agreement used in the study, such data should be deposited in one of the major public access-controlled repositories such as dbGAP (see link list at top right) or EGA (see link list at top right).	Clinical sample information is provided in EV datasets.
21. Computational models that are central and integral to a study should be shared without restrictions and provided in a machine-readable form. The relevant accession numbers or links should be provided. When possible, standardized format (SBML, CellML) should be used instead of scripts (e.g. MATLAB). Authors are strongly encouraged to follow the MIRIAM guidelines (see link list at top right) and deposit their model in a public database such as Biomodels (see link list at top right) or JWS Online (see link list at top right). If computer source code is provided with the paper, it should be deposited in a public repository or included in supplementary information.	Computational analyses are explain in high detail in the Appendix. All algorithms applied had been published before and can be looked up for further details.

G- Dual use research of concern

22. Could your study fall under dual use research restrictions? Please check biosecurity documents (see link list at top right) and list of select agents and toxins (APHIS/CDC) (see link list at top right). According to our biosecurity guidelines, provide a statement only if it could.	NA.
